# Developing 1,4-Diethyl-1,2,3,4-tetrahydroquinoxalin-substituted Fluorogens Based on GFP Chromophore for Endoplasmic Reticulum and Lysosome Staining

**DOI:** 10.3390/ijms251910448

**Published:** 2024-09-27

**Authors:** Daniil I. Rudik, Maxim M. Perfilov, Anatolii I. Sokolov, Cheng Chen, Nadezhda S. Baleeva, Ivan N. Myasnyanko, Alexander S. Mishin, Chong Fang, Yulia A. Bogdanova, Mikhail S. Baranov

**Affiliations:** 1 Institute of Bioorganic Chemistry, Russian Academy of Sciences, Miklukho-Maklaya 16/10, Moscow 117997, Russia; rudikdany@gmail.com (D.I.R.);; 2 Institute of Biochemical Technology and Nanotechnology, RUDN University, Miklukho-Maklaya 6, Moscow 117198, Russia; 3 Laboratory of Medicinal Substances Chemistry, Institute of Translational Medicine, Pirogov Russian National Research Medical University, Ostrovitianov 1, Moscow 117997, Russia; 4 Department of Chemistry, Oregon State University, 153 Gilbert Hall, Corvallis, OR 97331, USAchong.fang@oregonstate.edu (C.F.)

**Keywords:** green fluorescent protein (GFP), fluorogen, fluorescence microscopy, endoplasmic reticulum, lysosomes, bioimaging

## Abstract

In the present study, we demonstrated that the introduction of a 1,4-diethyl-1,2,3,4-tetrahydroquinoxalin moiety into the arylidene part of GFP chromophore-derived compounds results in the formation of environment-sensitive fluorogens. The rationally designed and synthesized compounds exhibit remarkable solvent- and pH-dependence in fluorescence intensity. The solvent-dependent variation in fluorescence quantum yield makes it possible to use some of the proposed compounds as polarity sensors suitable for selective endoplasmic reticulum fluorescent labeling in living cells. Moreover, the pH-dependent emission intensity variation of other fluorogens makes them selective fluorescent labels for the lysosomes in living cells.

## 1. Introduction

Fluorescent labeling is one of the most sensitive, selective, and therefore important techniques in biological research [1,2,3,4] and life sciences. Among the fluorescent labeling methods, the use of so-called fluorogens is a trend in modern developments [5]. Fluorogens are small molecules that do not fluoresce in solution; however, they become fluorescent when bound to specific targets. Nucleic acids [6,7,8,9], proteins [10,11,12], or whole cellular organelles [13,14,15,16,17,18,19] of living cells and organisms can be used as such targets for fluorogen-based labeling.

Due to the ease of synthesis and modification [20], as well as intense and diverse colors, arylidene imidazolones occupy an important place in the fluorogen-based labeling of living systems approaches [8,13,14,15,21]. The arylidene fragment of these molecules can readily isomerize, allowing them to nonradiatively release the excitation energy [22]. However, in certain environments provided by biomolecules or specific media, this isomerization pathway can become hindered, which enables the use of such substances as fluorogens [8,13,14,15,21].

**Scheme 1:**
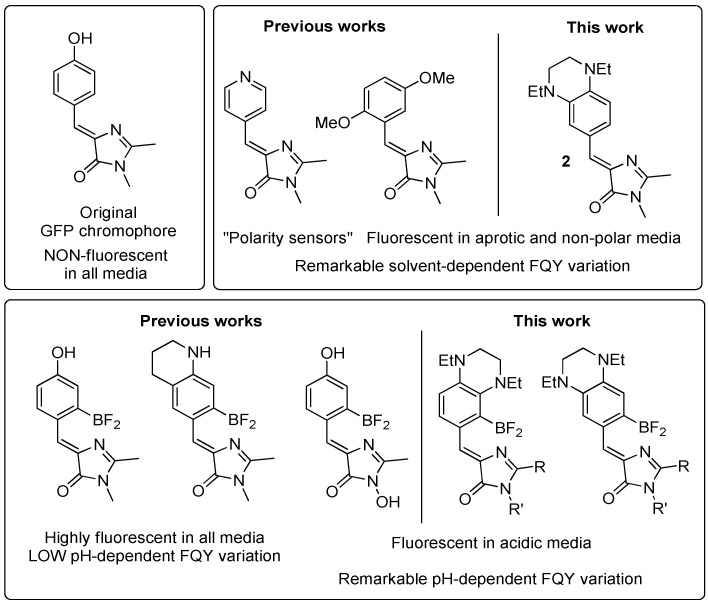
Analogues of GFP chromophore with low or high solvent- and pH-dependent FQY variations.

We and others have previously shown that the presence of various substituents in the arylidene fragment can cause local environment sensitivity in the fluorescence of arylidene imidazolones. Thus, the presence of a pyridine fragment [15], two methoxy groups [19], a trifluoromethyl group [13], and others [14] allows them to be used as “polarity sensors” for the fluorogen-based labeling of individual organelles (Scheme 1).

In recent studies, we have found that similar properties can also be possessed by molecules containing two substituents in the *para-* and *meta-*positions [23,24]. Since the presence of pH-sensitive groups is also of particular interest, in this work, we decided to study arylidene imidazolones containing 1,4-diethyl-1,2,3,4-tetrahydroquinoxalin moieties—see Scheme 1.

The unique combination of fluorogenic and lipophilic properties as well as the pH sensitivity of the proposed substances makes them good candidates for the membrane structures labeling of living cells. In this work, we have found that some of them are suitable for the selective labeling of the endoplasmic reticulum, while the others are suitable for lysosomes staining, which was successfully demonstrated by the fluorescence microscopy of living cells.

The endoplasmic reticulum (ER) is the largest membranous intracellular organelle—a dynamic structure consisting of tubules and sheets distributed throughout the cell and the nuclear envelope [25]. The ER is a major place of protein synthesis, transport, and folding, lipid synthesis, and calcium storage [26,27,28,29]. ER functions or malfunctions are often related to its shape [30]. Therefore, ER imaging could be important for the investigation of diseases and pertinent therapy development. Commercially available dyes for ER labeling have several limitations: some have an excitation maximum in the UV region of the spectra, which is a source of phototoxicity [31]. Other dyes label ER-related proteins instead of the lipid bilayer [32]. However, for precise and detailed ER visualization, the compounds with optimal spectral properties and membrane localization are needed.

Meanwhile, lysosomes are the main degradative compartments of eukaryotic cells [33]. The single bilayer membrane of lysosomes limits lumens rich in hydrolases; mature lysosomes are characterized by an internal pH of around 4.5–5.0 [34]. The hydrolytic enzymes play a crucial role in the decomposition of macromolecules, obtained from different types of endocytosis and autophagy [35,36]. Lysosomes participate in nutrient sensing and cell metabolism, growth, and autophagy regulation [37,38,39]. Different diseases have been linked to lysosomal malfunction including neurodegenerative disorders and lysosomal storage diseases [40,41]. Cargos (dextran, epidermal growth factor, bovine serum albumin) labeled with fluorescent dyes can be used for lysosomal imaging, although some of the available cargos are susceptible to degradation in the lysosome. This method is also time-consuming in some cases [42]. Fluorescence lifetime imaging microscopy is a powerful booming approach to various cell states analysis through endo- and exogenous fluorophores fluorescence lifetimes measurements [43,44,45]. The method allows lysosomal pH evaluation [46,47], although it requires a highly specialized technological base and is rarely used for mere visualization purposes. The simplest and fastest way to visualize lysosomes is to use pH-sensitive dyes that become fluorescent in an acidic environment. However, only a few examples of such fluorogenic dyes selective for lysosomes have been previously discovered [48,49,50].

## 2. Results and Discussion

**Scheme 2:**
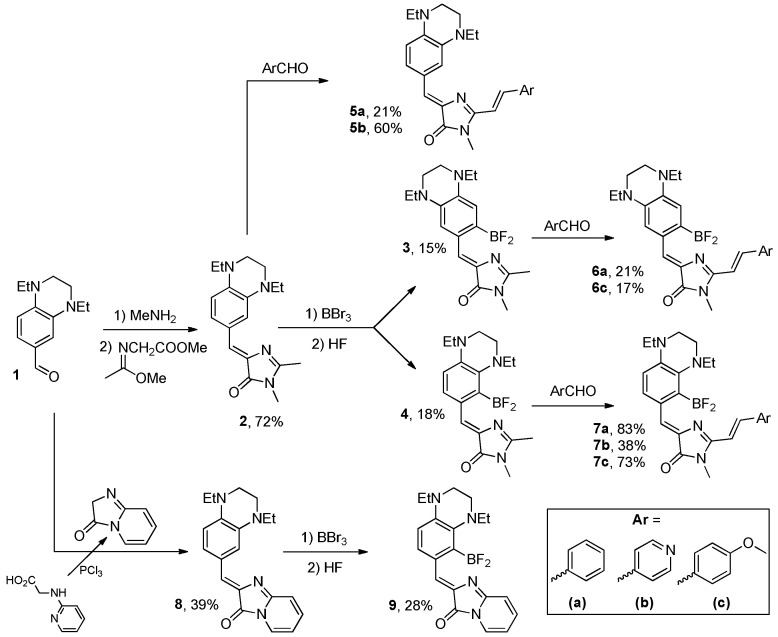
Synthesis of all compounds presented in this work.

We started our work with the synthesis of various arylidene imidazolinones containing the 1,4-diethyl-1,2,3,4-tetrahydroquinoxaline moiety. Using the corresponding aldehyde **1**, we synthesized a series of chromophores containing various substituents in the imidazolone fragment, as well as their conformationally locked analogs with a difluoroboryl group (Scheme 2, Section 3, Supplementary Material Parts S7 and S9).

The synthesis of the parent chromophore **2** was performed using the classical approach with the Schiff base and methyl((1-methoxy)amino)acetate [20]. Next, using this compound, we obtained its borylated derivatives **3** and **4**. For this purpose, we used our previously proposed method based on boron tribromide reaction, which was successfully used for the synthesis of a series of conformationally locked arylidene imidazolones [22,51]. It is interesting to note that, unlike many previous examples, in this case, two isomeric products **3** and **4** were obtained. Using compounds **2**–**4**, we synthesized derivatives **5**–**7** containing a styrene fragment in the second position of the imidazolone ring. It is well known that the introduction of such a group leads to a significant shift in the absorption and emission maxima to the long-wave region. In particular, the chromophore of the well-known fluorescent protein Kaede has a similar structure [52,53]. The introduction of such groups was performed according to the described approach using the reaction of chromophores **2**–**4** with various aromatic aldehydes [13,14,54]. To study the effect of substituents, in addition to unsubstituted benzaldehyde, we used *para*-anise aldehyde with an electron-donating methoxy group and an electron-accepting *para*-pyridine carbaldehyde. Unfortunately, we were unable to synthesize all nine corresponding products, since in many cases the reaction was characterized by very low yield and the formation of many side products. Finally, we synthesized derivative **8** and its conformationally locked analog **9**. The synthesis was performed according to the previously described protocol using pyridinylglycine [19,54]. It is well known that such products are also characterized by a pronounced bathochromic shift of the spectral maxima relative to unsubstituted arylidene imidazolones [13,15,54]. Unlike derivative **2**, the borylation of product **8** resulted in the formation of only one isomer **9**. Thus, in total, we synthesized twelve substances whose structures allow us to study the influence of the 1,4-diethyl-1,2,3,4-tetrahydroquinoxaline moiety on the optical properties of arylidene imidazolones, as well as to study their practical use in the fluorescent labeling of living cells.

Next, we studied the optical/photophysical properties of all the obtained chromophores in various solvents and at various pH levels—Table 1 and Table 2, Supplementary Material Parts S1 and S2.

**Figure 1:**
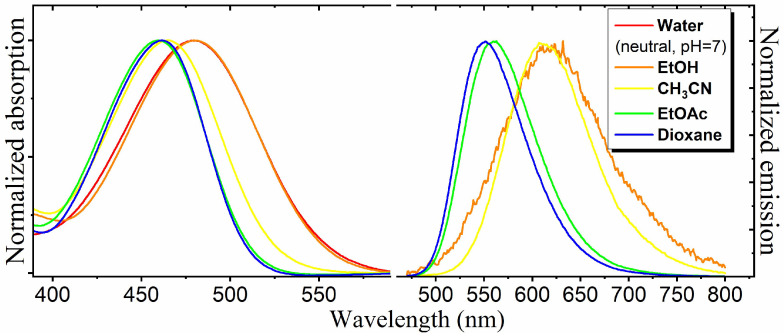
Normalized absorption and emission spectra of compound **2**.

We found that all the created chromophores demonstrate pronounced solvatochromism—in more polar and protonic solutions, we observed a noticeable bathochromic shift of the absorption spectra. However, only chromophore **2** is highly fluorescent in non-polar and aprotic media, and also demonstrates significant solvent-dependent variation in the fluorescence quantum yield (FQY) (Table 1, Figure 1).

**Figure 2:**
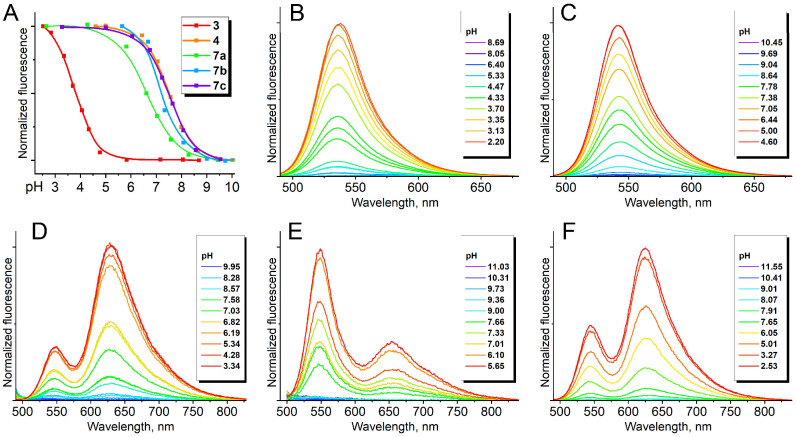
Fluorescence of compounds **3**, **4**, **7a**, **7b**, and **7c** at various pH. (**A**) Normalized fluorescence intensity at various pH for compounds **3** (540 nm), **4** (530 nm), **7a** (630 nm), **7b** (660 nm), and **7c** (640 nm). (**B**–**F**) Emission spectra at various pH for compounds **3** ((**B**), excitation 460 nm), **4** ((**C**), excitation 460 nm), **7a** ((**D**), excitation 480 nm), **7b** ((**E**), excitation 490 nm), and **7c** ((**F**), excitation 480 nm).

Despite the pronounced solvatochromism, styrene derivatives **5** as well as compound **8** were practically non-fluorescent in all media. Moreover, conformationally locked derivatives **6**, **7**, and **9** were also non-fluorescent in various solvents. Similar behavior has already been observed for several conformationally locked arylidene imidazolones and is apparently associated with a change in the character of frontier orbitals [55]. This phenomenon can be caused by the so-called “*meta effect*”, when the fluorescence of such chromophores is quenched because of the presence of strong electron-donating groups in the *meta*-position [24,56]. A study of the behavior of borylated chromophores at various pH levels showed that this effect can be eliminated in acidic conditions. Thus, derivatives **3**, **4**, and **7** demonstrate a significant pH-dependent variation in emission intensity and a fairly high FQY in acidic media—see Table 1 and Table 2 (which highlights with more information on p*K*_a_) and Figure 2 (which shows the change in compounds **3**, **4**, **7a**, **7b**, and **7c** fluorescence spectra at various pH).

We found that the compounds **3**, **4**, **7**, and **9** exhibited a noticeable change in their absorption and emission spectra at different pH (Supplementary Material Part S2, where the absorption (Figures S2.1–S2.6) and emission (Figures S2.13–S2.18) spectra of discussed compounds at various pH as well as titration curves (Figures S2.7–S2.12 and S2.19–S2.24) are presented). For compounds **4**, **7**, and **9,** the p*K*_a_ of these processes are in the neutral region of about 7–8, which corresponds to the p*K*_a_ of various dialkylanilines and obviously relates to the process of protonation of the nitrogen atom in the arylidene fragment. It can be assumed that such protonation occurs in the *meta-*position, since the nitrogen atom located in the *para-*position is subject to the electron-acceptor influence of the imidazolone fragment. Moreover, a lower p*K*_a_ in the region of 2–4 was previously established for such conformationally locked derivatives with an amine group in the *para-*position [51]. However, the isomeric compound **3** was characterized by a slightly lower p*K*_a_, which is probably explained by the different influence of the difluoroboryl group, which in these compounds is in the *ortho-*(compounds **4**, **7**, and **9**) or *para-*(compound **3**) positions relative to the protonated nitrogen atom. For compound **7b,** another pH-dependent transition of absorption spectra was revealed, which is associated with the pyridine fragment protonation (Supplementary Material Part S2).

**Scheme 3:**
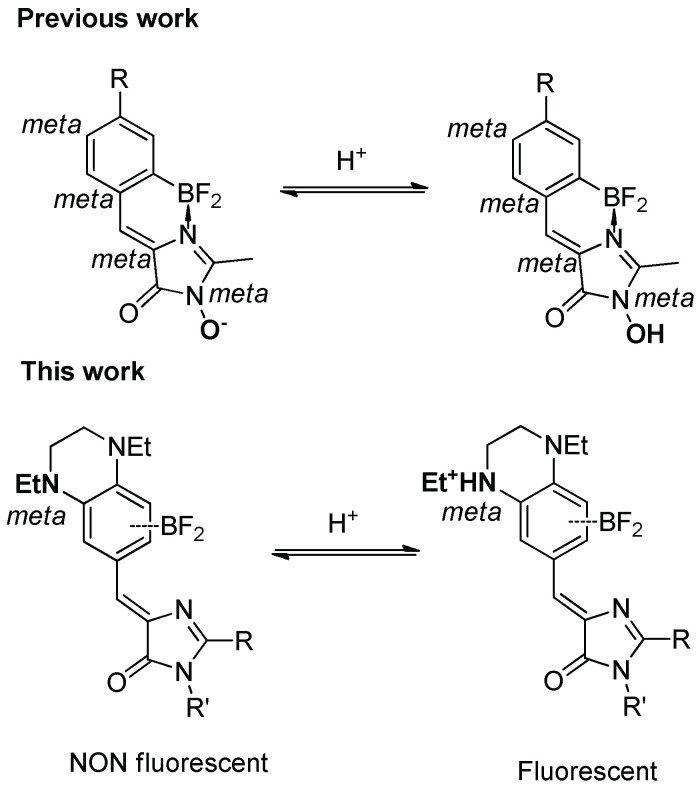
The nature of pH-dependent FQY variations in *meta*-substituted conformationally locked arylidene imidazolones presented in this work and proposed earlier [56].

We assume that protonation of the *meta*-substituent occurring in an acidic condition reduces its electron-donating strength and thereby neutralizes the “*meta-effect*” and leads to the fluorescence appearance. A similar phenomenon was observed earlier for N-hydroxylated derivatives, which can be considered as formal *meta*-substituted arylidene imidazolones [56]. For such derivatives, the deprotonation of the hydroxyl group leads to the appearance of a strongly electron-donating fragment, which also quenches the fluorescence—see Scheme 3.

It is interesting to note that we did not find any deviations between the p*K*_a_ calculated from the absorption and emission spectra. This suggests the absence of the excited state proton transfer phenomenon, which was previously observed for a wide variety of conformationally locked arylidene imidazolones [22,51].

In addition to fluorescence in solutions, an important characteristic of various chromophores is the ability to fluoresce in the solid state [57,58,59,60]. Unlike previously created polarity sensors based on arylidene imidazoles, for which the solid-state fluorescence was clearly visible [13], none of the compounds proposed in this work demonstrate such a phenomenon.

Revealed pH-dependent FQY variations allows the use of such compounds, not only as “polarity sensors”, but also as pH sensors, which are potentially suitable for living systems labeling. In this regard, at the next stage, we studied the behavior of all the proposed chromophores in HeLa Kyoto cell cultures—see Supplementary Material Part S3. Two different types of highlighted morphological structures were observed.

**Figure 3:**
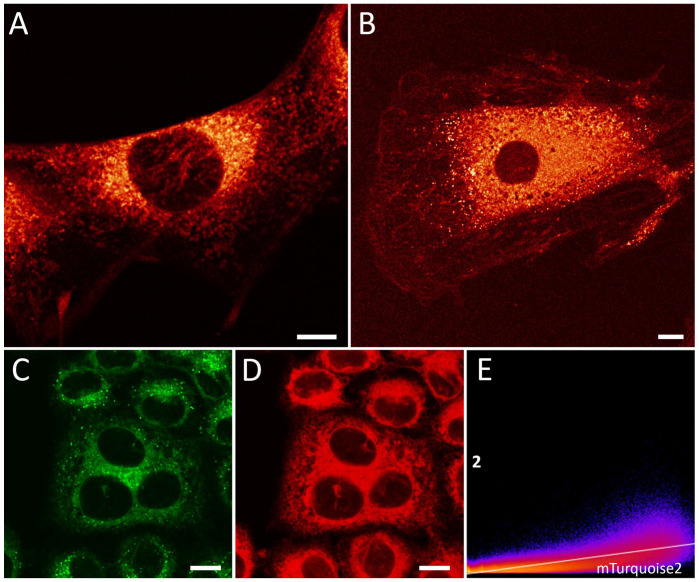
Live-cell imaging with chromophore **2** using confocal microscopy. ER-staining of (**A**) 3T3 NiH and (**B**) h9c2 cells by 5 μM of **2** (added from 1 mM DMSO stock solution, incubation 1 min). Images were pseudo-colored using with Fiji Lookup Tables “NanoJ-Orange” grading. (**C**–**E**) Co-localization analysis of 5 μM of chromophore **2** in HeLa Kyoto cells compared to 1 μM ER-tracker Red (Invitrogen, added from 1 mM stock in DMSO, incubation 5 min). Pearson co-localization analysis followed by Costes randomized test resulted in 0.70. (**E**) 2D intensity histogram of fluorescence from green (**2**, shown in green pseudo-color) and red (ER-tracker Red, shown in red pseudo-color) channels. Scale bars are 10 μm (**A**–**D**).

First, we revealed that the presence of the unlocked compounds **2**, **5a**, **5b**, and **8** in the imaging media led to the development of the fluorescent signal associated mostly with ER structures (Figure 3). Since compound **2** demonstrates the highest FQY variation between a polar and non-polar environment, we used it for more detailed studies with various cell lines. Therein, using this chromophore, we showed ER staining in 3T3 NiH and h9c2 cells (Figure 3A,B), similar to ER staining in HeLa Kyoto cells: the clearly distinguishable tubular net system with higher density in the perinuclear region. Staining with compound **2** showed a reasonable co-localization level with commercially available ER-tracker Red (Invitrogen, Waltham, MA, USA) in HeLa Kyoto cells: the corrected Pearson coefficient was 0.70 (Figure 3C–E). The relatively average value of the co-localization coefficient can be explained by the additional staining of lipid droplets caused by compound **2** (this is clearly seen in Figure 2C in comparison with Figure 3D); this phenomenon is common for polarity-sensitive fluorogens [61]. A comparative study of the photostability of the staining provided by compound **2** with the widely used fluorescent protein Turquoise 2, which has similar positions of absorbance and emission spectra, showed the significant superiority of compound **2** labeling (see Figure S4.1 in the Supplementary Materials for the comparative plots for photobleaching measurements of compound **2** versus mTurquoise2). We observed a very slight decrease in the fluorescent signal of chromophore **2** during prolonged exposure to intense irradiation under confocal microscopy conditions. Notably, this finding can be explained by the possible exchange of free chromophore in solution with the chromophore in the ER. This exchange provides the constant restoration of fluorescence, which is one of the significant advantages of fluorogenic labeling in contrast to the use of conventional fluorescent labels.

**Figure 4:**
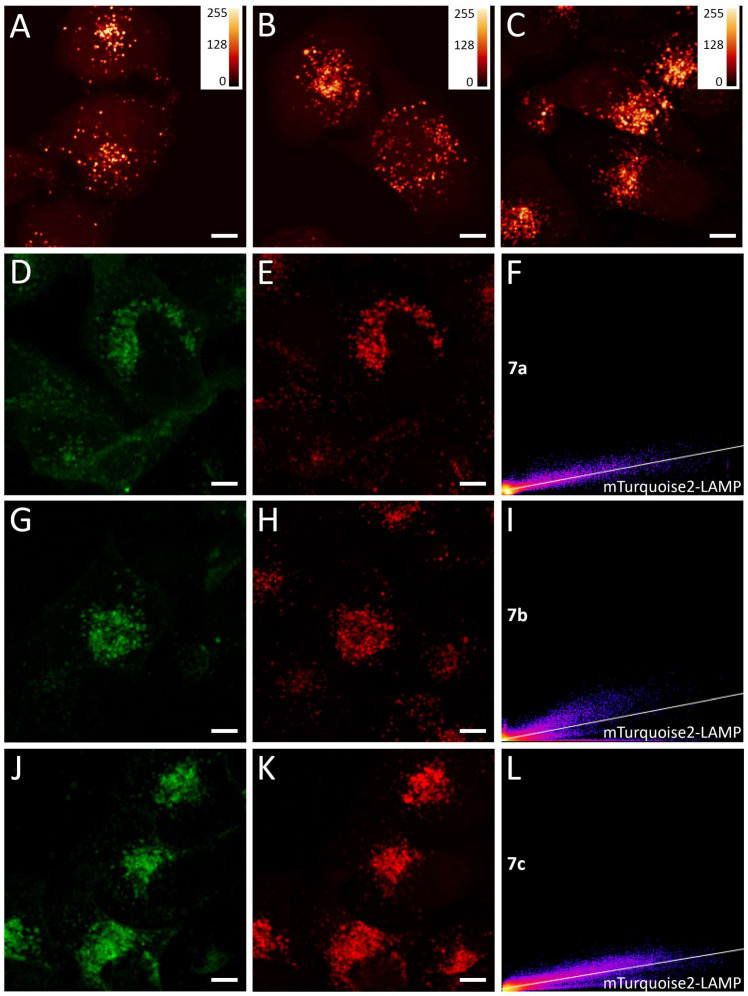
Live-cell imaging with chromophores **7a**, **7b**, and **7c** using confocal microscopy. Lysosomes staining of HeLa Kyoto cells by (**A**) 5 μM of **7a** (added from 10 mM DMSO stock solution, incubation 1 min), (**B**) 1 μM of **7b** (added from 1 mM DMSO stock solution, incubation 1 min), and (**C**) 5 μM of **7c** (added from 10 mM DMSO stock solution, incubation 1 min). Images were pseudo-colored using Fiji Lookup Tables “NanoJ-Orange” grading. (**D**–**F**) Co-localization analysis of 5 μM of **7a** chromophore in HeLa Kyoto cells compared to LAMP-1-mTurquoise2. Pearson co-localization analysis followed by Costes randomized test resulted in 0.86. (F) 2D intensity histogram of fluorescence from green (LAMP-1-mTurquoise2, shown in green pseudo-color) and red (**7a**, shown in red pseudo-color) channels. (**G**–**I**) Co-localization analysis of 1 μM of **7b** chromophore in HeLa Kyoto cells compared to LAMP-1-mTurquoise2. Pearson co-localization analysis followed by Costes randomized test resulted in 0.72. (**I**) 2D intensity histogram of fluorescence from green (LAMP-1-mTurquoise2, shown in green pseudo-color) and red (**7b**, shown in red pseudo-color) channels. (**J**–**L**) Co-localization analysis of 5 μM of **7c** chromophore in HeLa Kyoto cells compared to LAMP-1-mTurquoise2. Pearson co-localization analysis followed by Costes randomized test resulted in 0.87. (**L**) 2D intensity histogram of fluorescence from green (LAMP-1-mTurquoise2, shown in green pseudo-color) and red (**7c**, shown in red pseudo-color) channels. Scale bars are 10 μm (**A**–**E**,**G**,**H**,**J**,**K**).

Second, we found that the conformationally locked compounds **7a**, **7b**, and **7c** stained spherical intracellular structures in the red fluorescence channel (Figure 4). Due to the pronounced pH-sensitivity of the FQY of these compounds, we suggested the lysosomal nature of these stained structures. The lysosomal lumen is characterized by a pH around 4.5–5.0 [34], thus making this cellular compartment optimal for the fluorescence of the protonated form of compounds **7**. To test this idea, we measured the co-localization of **7a**, **7b**, and **7c** with LAMP-1-mTurquoise2. LAMP-1 (lysosome-associated membrane protein 1) is one of the most abundant proteins of the lysosomal membrane, and fluorescent proteins fused to LAMP-1 are commonly used for lysosomes labeling [62]. The co-localization analysis resulted in corrected Pearson coefficient values between 0.72 for compound **7b** and more than 0.85 for compounds **7a** and **7c,** which confirm our hypothesis.

To study the photostability of staining with chromophores **7**, we used another fluorescent protein with a more suitable position of the absorption and emission spectra—mVenus. In contrast to derivative **2**, compounds **7a** and **7b** demonstrated photostability comparable to mVenus, while **7c** staining turned out to be slightly superior in this respect (see Figure S4.2 in the SI for the comparative plots for photobleaching measurements of compounds **7a**, **7b**, and **7c** versus mVenus). Apparently, for these fluorogens, the exchange of chromophore between the free form in solution and in lysosomes occurs more slowly than the bleaching that occurs under microscopic conditions.

It is known that in addition to low pH, lysosomes are also characterized by increased viscosity. In this regard, we studied the behavior of compounds **7a**, **7b**, and **7c** in mixtures of water with glycerol at low pH and found that the increase in viscosity caused by the addition of glycerol also increases their fluorescence intensity (Supplementary Material Part S5, Table S5.1). It can be assumed that this effect also determines the fluorescence of these fluorogens in lysosomes.

Fluorescence lifetime imaging microscopy (FLIM) is a promising technology for the analysis of living systems characteristics, including pH, based on the environment-dependent changes in fluorescence lifetimes of endogenous molecules or specific sensitive dyes. In this regard, we have studied pH-dependent changes in fluorescence lifetimes of compounds **7a**, **7b**, and **7c** using a miniTau spectrophotometer. The investigation of the fluorescence decay curves in water solutions with various pH indeed showed changes in the fluorescence lifetimes corresponding to the pH-dependent transitions of these dyes (see Supplementary Material Part S6, Tables S6.1–S6.3). For all three, at pH below 7, there was a slight decrease in the amplitude-weighted average fluorescence lifetime. However, this effect was quite small (0.2–0.25 ns), which suggests that it may not be used for practical applications. Moreover, the fact that all of the dyes displayed bi-exponential decay curves makes their possible use in FLIM rather sophisticated. A more pronounced effect was observed for compound **7b**, which can undergo double protonation. The doubly protonated form had a lifetime of about 2 ns, whereas the neutral and monoprotonated forms had lifetimes of 0.5 and 0.3 ns, respectively. However, the transition to the doubly protonated form occurs at pH < 4, which limits the potential for using this phenomenon in living systems labeling.

It should be noted that all studied cell-imaging compounds did not cause any visible toxicity throughout the experimental procedure (for up to two hours).

As in all other cases where we have observed selective fluorescent staining of ER using arylene imidazolone-based chromophores (see ref [13] for structural analysis of selective ER dyes), we did not observe any clear correlation between the structure of the chromophore and selectivity in this work. As in previous cases, selectivity appears due to some random yet beneficial combination of the required degree of hydrophobicity and high FQY in non-polar and aprotic media. Similarly, the selective staining of lysosomes was also observed only for chromophores **7a**, **7b**, and **7c**, but not for the entire group of pH-sensitive derivatives. Moreover, fluorogens **3**, **4**, and **9**, which are brighter in acidic media, did not provide such selective labeling. In essence, selectivity is determined not only by pH-sensitive properties, but also by a successful combination with the required degree of lipophilicity, which in this case is determined by the presence of a styrene fragment.

## 3. Materials and Methods

### 3.1. Synthesis

Commercially available reagents (Merck (Darmstadt, Germany), ABCR (Karlsruhe, Germany), BLDPharm (Shanghai, China), Fluorochem (Hadfield, UK), and TCI (Tokyo, Japan)) were used without additional purification.

E. Merck Kieselgel 60 was used for purification via flash column chromatography.

Thin layer chromatography was performed on silica gel 60 F254 plates (Merck, #cat 105554 and #cat 100390). The visualization of TLC was performed by UV light (254 nm).

700 MHz Bruker Avance III NMR, Bruker Avance 800, Bruker Fourier 300 and Bruker AVANCE III 600 MHz (Bruker, Billerica, MA, USA) at 303 K were used for NMR study. The chemical shifts were reported relative to the residual solvents peaks.

The melting points were analyzed using SMP 30 apparatus.

High-resolution mass spectrometry (HRMS) spectra were recorded on an AB Sciex TripleTOF 5600+ instrument (AB Sciex instrument, Framingham, MA, USA) using electrospray ionization (ESI). The measurements were conducted in a positive ion mode (interface capillary voltage—5500 V).

The description and copies of the obtained NMR spectra, the description of HRMS spectra, as well as the appearance and melting points of the obtained compounds, are presented in Supplementary Materials.

#### 3.1.1. 1,4-Diethyl-1,2,3,4-tetrahydroquinoxaline

Quinoxaline (11 g, 85 mmol) was dissolved in dry benzene (250 mL) and the reaction mixture was cooled to 5 °C. Next, sodium borohydride (32.2 g, 0.85 mol) was added over a period of 15 min. The resulting pale-yellow slurry was stirred for 30 min at 5 °C and then glacial acetic acid (130 mL) was added dropwise over 1 h. After maintaining the temperature at 10 °C for 1 h, the mixture was heated to reflux for 6 h. Next, the mixture was cooled to room temperature and water (70 mL) was added dropwise to quench excess sodium borohydride. The mixture was extracted with EtOAc (3 × 100 mL), and its organic layers separated, combined, and dried over Na_2_SO_4_. Solvent was removed in vacuo and the resulting residue was purified via flash chromatography (eluent—mixture of hexane and ethyl acetate, *v/v* 1:1).

#### 3.1.2. 1,4-Diethyl-1,2,3,4-tetrahydroquinoxaline-6-carbaldehyde **1**

1,4-Diethyl-1,2,3,4-tetrahydroquinoxaline (10.5 g, 55 mmol) was dissolved in dry DMF (25 mL) and cooled to 0 °C. POCl_3_ (2.0 mL, 20.1 mmol) was added within 10 min and the mixture was stirred for 30 min at 0 °C. Next, the mixture was heated to 40 °C and stirred for 4 h. The reaction mixture was then cooled to room temperature and poured into ice water. 1 M NaOH solution was added to reach pH = 9. The mixture was extracted with EtOAc (3 × 100 mL). Combined organic layers were washed with brine (3 × 50 mL) and dried over Na_2_SO_4_. Solvent was removed in vacuo and the resulting residue was purified using flash chromatography (eluent—mixture of hexane and ethyl acetate, *v/v* 5:1).

#### 3.1.3. (Z)-5-((1,4-Diethyl-1,2,3,4-tetrahydroquinoxalin-6-yl)methylene)-2,3-dimethyl-3,5-dihydro-4H-imidazol-4-one **2**

1,4-Diethyl-1,2,3,4-tetrahydroquinoxaline-6-carbaldehyde **1** (4.5 g, 20.6 mmol) was dissolved in CHCl_3_ (80 mL), and mixed with 35% methylamine solution (15 mL, 123 mmol in isopropanol) and Na_2_SO_4_ (10 g). The mixture was stirred for 48 h at room temperature and filtered. The solvent was evaporated, and anhydrous methanol (40 mL) and (3.9 g, 26.8 mmol) of methyl((1-methoxy)amino)acetate were then added and the mixture was stirred for 24 h at room temperature. Solvents were removed in vacuo and the resulting residue was purified with column chromatography (eluent—mixture CH_2_Cl_2_: EtOH *v/v* 100:2).

#### 3.1.4. Compounds **5a** and **5b**

(Z)-5-((1,4-diethyl-1,2,3,4-tetrahydroquinoxalin-6-yl)methylene)-2,3-dimethyl-3,5-dihydro-4*H*-imidazol-4-one **2** (156 mg, 0.5 mmol) and the corresponding aldehyde were dissolved in pyridine (5 mL). Piperidine was added (10 μL) and the mixture was heated at 100 °C for 24 h. The reaction mixture was then cooled to room temperature. Solvents were removed in vacuo and the resulting residue was purified with column chromatography (eluent—mixture of CH_2_Cl_2_ and EtOH, *v/v* 100:1).

#### 3.1.5. Compounds **3**, **4**, and **9**

Corresponding imidazolone (9.6 mmol) was dissolved in 1,2-dichloroethane (250 mL); molecular sieves (20 g 3 Å and 20 g 4 Å) and 1 M boron tribromide solution in dichloroethane (48 mmol, 48 mL) were added and the mixture was refluxed for 3 h. The reaction mixture was then cooled to room temperature and filtered; the sieves were washed with EtOH (3 × 50 mL) and EtOAc (2 × 100 mL). Next, the solution of hydrofluoric acid (40% aqueous, 50 mL) was added to combined organic solution and the mixture was stirred at room temperature for 40 min. The resulting mixture was washed with NaHCO_3_ saturated solution (5 × 150 mL) and brine (3 × 150 mL) and dried over Na_2_SO_4_. Solvents were removed in vacuo and the resulting residue was purified with column chromatography (gradient elution: mixture of hexane and EtOAc, *v/v* 75:25 with 1% of AcOH then EtOAc with 1% of AcOH).

#### 3.1.6. Compounds **6a**, **6c** and **7a**, **7b**, **7c**

(Z)-5-((5-(difluoroboraneyl)-1,4-diethyl-1,2,3,4-tetrahydroquinoxalin-6-yl)methylene)-2,3-dimethyl-3,5-dihydro-4*H*-imidazol-4-one or (Z)-5-((7-(difluoroboraneyl)-1,4-diethyl-1,2,3,4-tetrahydroquinoxalin-6-yl)methylene)-2,3-dimethyl-3,5-dihydro-4*H*-imidazol-4-one (72 mg, 0.2 mmol) and corresponding aldehyde (1 mmol) were dissolved in pyridine (5 mL). Piperidine was added (10 μL) and the mixture was heated at 100 °C for 2 h. The reaction mixture was then cooled to room temperature. Solvents were removed in vacuo and the resulting residue was purified with column chromatography (eluent—mixture of CH_2_Cl_2_ and EtOH, *v/v* 100:1).

#### 3.1.7. (Z)-2-((1,4-Diethyl-1,2,3,4-tetrahydroquinoxalin-6-yl)methylene)imidazo[1,2-a]pyridin-3(2H)-One **8**

Pyridin-2-ylglycine hydrochloride (900 mg, 4.8 mmol) was suspended in PCl_3_ (8 mL) in an argon atmosphere and refluxed for 3 h. PCl_3_ was removed in vacuo. Pyridine (10 mL), triethylamine (2 mL), and 1,4-Diethyl-1,2,3,4-tetrahydroquinoxaline-6-carbaldehyde **1** (1.15 g, 5.3 mmol) were added in the argon atmosphere. The resulting mixture was stirred at 90 °C for 18 h. The reaction mixture was then cooled to room temperature. Solvents were removed in vacuo and the resulting residue was diluted with water (50 mL). The mixture was extracted with EtOAc (1 × 100 mL). Combined organic layers were washed with brine (3 × 50 mL) and dried over Na_2_SO_4_. Solvent was removed in vacuo and the resulting residue was purified via flash chromatography (eluent—mixture of hexane and ethyl acetate, *v/v* 1:1).

### 3.2. UV-VIS Absorption and Emission Spectra

Fluorescence excitation and emission spectra were recorded with an Agilent Cary Eclipse fluorescence spectrophotometer (Agilent, Santa Clara, CA, USA). UV-VIS spectra were recorded with a Varian Cary 100 spectrophotometer (Varian, Santa Clara, CA, USA).

The calculation of fluorescence quantum yields was performed according to the protocol described previously [63] with the use of rhodamine 101 (R101), rhodamine 6G (R6G), and fluorescein as standards. The quantum yield was calculated by the following formula:

(1) $$\Phi_{x}=\Phi_{st}\times \frac{F_{x}}{F_{st}}\times \frac{1-10^{-A_{st}}}{1-10^{-A_{X}}}\times \frac{n_{x}^{2}}{n_{st}^{2}}$$

where F is the area under the emission peak, f is the absorption factor (see below), n is the refractive index of the solvent, Φ is the quantum yield, A is absorption at the excitation wavelength, the subscript x corresponds to the novel compounds, and the subscript st corresponds to the standards.

All measurements were performed with 10–30 µM solutions for absorbance and 1–3 µM for fluorescence.

pH titration was performed with an Ohaus Starter 2100 pH meter (Ohaus, Parsippany, NJ, USA). All measurements were performed with 3–5 µM solutions of chromophores in 5 mM PBS with EtOH addition 10% (*v*/*v*) due to the poor solubility of dyes in alkali solutions.

Viscosity dependences of fluorescence quantum yields were investigated in a mixture of 5 mM PBS and glycerol; H_3_PO_4_ or NaOH was added to adjust the pH to the desired value. The pH was monitored with an Ohaus Starter 2100 pH meter.

### 3.3. DNA Cloning

mTurquoise2 fused to lysosome-targeting protein LAMP1, ER-localized mTurquoise2 fused to a combination of N-terminal prolactin and C-terminal KDEL signal sequences, and mVenus fused to vimentin were cloned using the Golden Gate assembly, following the MoClo syntax [64,65]. The constructs were put under a CMV promoter and possessed a SV40 poly(A) sequence. Eco31I (BsaI) restriction endonucleases (Thermo Scientific, Waltham, MA, USA) and T4 DNA ligase (Evrogen, Moscow, Russia) were used for the cloning procedure. All level 0 plasmids were available inhouse.

### 3.4. Cell Culture

HeLa Kyoto, h9c2 (rat cardiomyoblasts), and 3T3 NiH fibroblasts cell lines were obtained from established frozen stocks of our laboratory. Cells were seeded into 35 mm glass-bottom dishes (SPL Life Sciences, Pocheon-si, Gyeonggi-do, Republic of Korea) and cultured in Dulbecco’s Modified Essential Medium (DMEM) with 2 mM glutamine and 4.5 g/L glucose (PanEco, Moscow, Russia) supplemented with 10% fetal bovine serum (HyClone, Thermo Scientific, Waltham, MA, USA) and 1% Penicillin + Streptomycin (5000 U/mL + 5000 μg/mL, PanEco) in a humidified atmosphere under 37 °C and 5% CO_2_ for 24 h. After 24 h cells, the were imaged.

### 3.5. Live Cell Widefield Imaging

For imaging, the cells were incubated with 1.0–10 μM of chromophores (added from the 10 mM stock solution in DMSO) in 1 mL of imaging media Hank’s Balanced Salt Solution with calcium and magnesium (PanEco) and were supplemented with 20 mM HEPES (pH 7.1, Sigma-Aldrich, Darmstadt, Germany) at room temperature for 1 min. Primary chromophores’ testing was performed using a widefield BZ-9000 inverted fluorescence microscope (Keyence, Osaka, Japan). The imaging was performed with a 60 × PlanApo 1.40 NA oil objective (Nikon, Melville, NY, USA); a GFP-B filter (Keyence, Ex. 470/40 nm, DM 495 nm, BA 535/50 nm), a TRITC filter (Keyence, Ex. 540/25 nm, DM 565 nm, BA 605/55 nm), and a TxRed filter (Keyence, Ex. 560/40 nm, DM 595 nm, BA 630/60 nm) were used.

### 3.6. Compound **2** Co-Localization Analysis

For co-localization analysis, HeLa Kyoto cells were also incubated in imaging media with 1 μM of ER-tracker Red (added from the 1 mM stock solution in DMSO) and 5 μM of **2** (added from 1 mM DMSO) for 5 min. Confocal images were acquired with a TSC SP2 confocal system (Leica Microsystems, Wetzlar, Germany) installed on an inverted fluorescent microscope Leica DM IRE equipped with an HCX PL APO Lbd.BL 63 × 1.40 oil immersion lens and an argon (458/488 nm) laser. Image acquisition was performed using the 458 nm line of the argon laser (≈6.1 μW), 400 Hz, with the emission of 470–530 nm for mTurquoise2 imaging and 507–631 nm for **2** imaging; the emission was collected with a GaAsP detector. Images were acquired as 1024 × 1024 pixels (60 × 60 μm) 8-bit .lei files, and were processed and analyzed for co-localization using Fiji ImageJ distribution (ver. 1.52n) [66] and NanoJ plug-in (ver. 1.14stable1) [67].

### 3.7. Compound **2** Photostability Study

We performed the photobleaching analysis of **2** in HeLa Kyoto cells with ER-localized mTurquoise2 as a reference. A subset of reference cells was transfected by a mixture of 1.5–2 ng DNA and 5 μL FuGENE HD transfection reagent (Promega, Madison, WI, USA) in 100 μL OptiMEM (PanEco) per dish and imaged in imaging media 24 h after transfection. A second subset of non-transfected HeLa Kyoto cells was incubated in imaging media with 5 μM of **2** (added from 1 mM DMSO) for 1 min. Images of both cell subsets were acquired with a TSC SP2 confocal system based on an inverted fluorescent microscope Leica DM IRE equipped with an HCX PL APO Lbd.BL 63 × 1.40 oil lens and an argon (458/488 nm) laser; emission was collected at 507–631 nm with a GaAsP detector. For bleaching, a 14 × 10^3^ μm^2^ region was scanned at 0.3 fps with 6.1 μW, 400 Hz of the 458 nm line of an argon laser. Images were acquired as 1024 × 1024 pixels 8-bit .lei files, which were processed and analyzed using Fiji.

### 3.8. Compounds **7a**, **7b**, and **7c** Co-Localization Analysis

For the co-localization analysis of **7a**, **7b,** and **7c** in HeLa Kyoto cells, they were transfected with a plasmid-coding LAMP-1-mTurquoise2 through a mixture of 2 ng DNA and 6 μL FuGENE HD transfection reagent (Promega) in 100 μL OptiMEM (PanEco) per dish. Then, 24 h after transfection, the cells were incubated in imaging media with 1 μM of **7b** (added from 1 mM DMSO), 5 μM of **7a** or 5 μM of **7c** (added from 10 mM DMSO) for 5 min. Confocal images were acquired with a TSC SP2 confocal system (Leica Microsystems) installed on an inverted fluorescent microscope Leica DM IRE equipped with an HCX PL APO Lbd.BL 63 × 1.40 oil immersion lens, and argon (458 nm) and HeNe (514 nm) lasers. Image acquisition was performed using the 458 nm line of the argon laser (~6.1 μW), 200 Hz, with the emission 470–510 nm for mTurquoise2 imaging and the 514 nm line (~8.7 μW), 200 Hz, with the emission 525–640 nm for **7a**, **7b,** and **7c** imaging. The emission was collected with a GaAsP detector. Images were acquired as 512 × 512 pixels (60 × 60 μm) 8-bit .lei files and were processed and analyzed for co-localization using Fiji ImageJ distribution (ver. 1.52p). The background signal was subtracted (Process/Subtract background) and the noise was reduced (Process/Noise/Despeckle) in both channels; then, co-localization was estimated using the coloc2 plug-in. Pseudo-colors were used for final images visualization.

### 3.9. Compounds **7a**, **7b**, and **7c** Photostability Study

We performed the photobleaching analysis of **7a**, **7b,** and **7c** in HeLa Kyoto cells with mVenus as a reference. A subset of reference cells was transfected by a mixture of 2 ng DNA and 6 μL FuGENE HD transfection reagent (Promega) in 100 μL OptiMEM (PanEco) per dish and imaged in imaging media 24 h after transfection. A second subset of non-transfected HeLa Kyoto was incubated in imaging media with 5 μM of **7a** or **7c** (added from 10 mM DMSO) or 1 μM of **7b** (added from 1 mM DMSO) for 1 min. Images of both cell subsets were acquired with a TSC SP2 confocal system based on inverted fluorescent microscope Leica DM IRE equipped with an HCX PL APO Lbd.BL 63 × 1.40 oil lens and an HeNe (514 nm) laser. For bleaching, the 14 × 10^3^ μm^2^ region was scanned at 3 fps with (≈8.7 μW), 1000 Hz, of the 514 nm line of an HeNe laser, and emission was collected at 525–640 nm with a GaAsP detector. Images were acquired as 512 × 512 pixels 8-bit .lei files and were processed and analyzed using Fiji.

### 3.10. Fluorescence Lifetime Measurements In Vitro

Compounds **7a**, **7b**, and **7c** were dissolved in water (from 10 mM DMSO solution) with different pH values (3–9) to a final concentration of 10 µM at room temperature. Lifetime measurements were made using a miniTau TCSPC spectrometer (Edinburgh Instruments, Livingston, UK); a 50 ns window divided into 2048 time channels was used. The fluorescence was excited with the PLS/500 picosecond optical pulse generator (Integrated Optical Systems), with a central emission wavelength of 500 nm and a repetition rate of 20 MHz. The photons were counted in the spectral range of 625–700 nm for up to 3000 counts. The data processing and visualization were performed using Fluoracle 2.5.1. suite (Edinburgh Instruments). The fitting of fluorescent decay curves was performed by deconvolution with the instrument response function (IRF-based fit).

## 4. Conclusions

In this work, we propose a new structural motif that brings the environment-sensitive variation of fluorescence quantum yield to arylidene imidazolones. We have shown that the introduction of 1,4-diethyl-1,2,3,4-tetrahydroquinoxaline moieties into the arylidene part of these molecules results in the solvent- or pH-dependent variation of fluorescence intensity.

The solvent-dependence of the fluorescence quantum yield was observed for classical derivatives without additional conformational fixation. The effect was especially pronounced for derivative **2**. As in many previous examples, the maximum fluorescence intensity was observed in non-polar and aprotic media. Such properties allow this derivative to be used as a selective fluorescence dye for the endoplasmic reticulum of living cells. Unlike commercially available ER trackers that fluorescently stain specific proteins in ER, our proposed dye stains the ER exclusively as a “polarity sensor”, with favorable lipophilic properties leading to its accumulation in ER. Due to this appealing property, the proposed dye is mostly free of the corresponding shortcomings of existing trackers, such as incorrect ER labeling due to the absence or low expression of these proteins.

The conformationally locked derivatives were found to be non-fluorescent in almost all conditions except acidic pH. Apparently, this phenomenon is associated with the so-called “*meta-effect*” when the fluorescence of arylidene imidazolones is diminished in the presence of strong electron-donating groups in the *meta-*position of the arylidene part of the molecule. The pH decrease leads to protonation of the *meta*-amino group and the disappearance of this effect. As a result, these molecules are fluorescent at low pH and can be used as pH sensors. The p*K*_a_ of these proposed fluorogens lies in the range of physiological values and they can therefore selectively stain the lysosomes of living cells.

The selectivity of labeling for both types of fluorogens was confirmed by co-localization experiments with known trackers of these organelles. We demonstrated the high photostability of labeling and the low toxicity of the proposed fluorogens in short-term (several hours) experiments. We envision that this work, through combined rational design, organic synthesis, molecular spectroscopy, and live cell imaging, has effectively contributed to the expanding toolset for biomimetic sensors and probes to advance non-invasive and sensitive imaging microscopy across life sciences and engineering fields.

## Figures and Tables

**Table 1:** Optical properties of the newly synthesized chromophores in various solvents.

Compound		H_2_O (Neutral, pH ≈ 6)	H_2_O (Acidic, pH ≈ 3)	EtOH	CH_3_CN	EtOAc	Dioxane
**2**	Absorption max., nm	480	- ^a^	480	464	460	462
	*ε*, (M cm)^−1^	23,500		24,500	25,500	26,000	25,000
	Emission max., nm	- ^b^		≈620	≈610	560	552
	FQY ^e^, %			<1	1.5	12	11
**3**	Absorption max., nm	552	475	568	555	550	552
	*ε*, (M cm)^−1^	16,500	27,000	23,000	22,500	24,500	25,500
	Emission max., nm	- ^b^	539	≈590	≈580	≈570	≈570
	FQY ^e^, %		14	<1	<1	<1	<1
**4**	Absorption max., nm	522	480	516	505	502	505
	*ε*, (M cm)^−1^	46,000	39,500	16,500	17,000	16,500	17,000
	Emission max., nm	- ^b^	547	≈540	≈540	≈550	≈550
	FQY ^e^, %		92	<1	<1	<1	<1
**5a**	Absorption max., nm	593	- ^a^	554	542	536	536
	*ε*, (M cm)^−1^	15,500		30,000	28,000	29,500	28,000
	Emission max., nm	- ^b^				670	660
	FQY ^e^, %					<1	1.8
**5b**	Absorption max., nm	579	- ^a^	580	562	558	559
	*ε*, (M cm)^−1^	14,500		25,000	23,500	22,500	23,000
	Emission max., nm	- ^b^					≈715
	FQY ^e^, %						<1
**6a**	Absorption max., nm	- ^c^		658	648	643	645
	*ε*, (M cm)^−1^			5500	19,500	22,500	22,500
	Emission max., nm	- ^b^					
	FQY ^e^, %						
**6c**	Absorption max., nm	- ^c^	654	642	639	640	- ^c^
	*ε*, (M cm)^−1^		31,500	31,000	33,500	35,000	
	Emission max., nm	- ^b^					
	FQY ^e^, %						
**7a**	Absorption max., nm	591	539	592	584	583	585
	*ε*, (M cm)^−1^	20,000	17,000	35,500	34,000	32,000	32,500
	Emission max., nm	- ^b^	639	- ^b^			
	FQY ^e^, %		2.0				
**7b**	Absorption max., nm	601	558/578 ^d^	609	599	596	599
	*ε*, (M cm)^−1^	28,000	22,500/23,000 ^d^	26,000	25,000	23,500	24,500
	Emission max., nm	- ^b^	662/- ^b,d^	- ^b^			
	FQY ^e^, %		1.0/- ^b,d^				
**7c**	Absorption max., nm	596	546	594	583	580	583
	*ε*, (M cm)^−1^	25,000	17,000	36,000	34,000	32,500	34,000
	Emission max., nm	-^b^	634	- ^b^			
	FQY ^e^, %		2.8				
**8**	Absorption max., nm	586	- ^a^	588	576	575	578
	*ε*, (M cm)^−1^	41,000		48,000	45,000	45,000	47,500
	Emission max., nm	- ^b^				662	643
	FQY ^e^, %					<1	2.0
**9**	Absorption max., nm	608	553	607	596	598	601
	*ε*, (M cm)^−1^	58,000	50,500	94,500	83,000	80,000	84,500
	Emission max., nm	- ^b^	608	- ^b^			
	FQY ^e^, %		27				

^a^—not measured; ^b^—non-fluorescent (FQY < 0.1%); ^c^—not soluble; ^d^—doubly protonated form; ^e^—fluorescence quantum yield.

**Table 2:** Optical properties of proposed chromophores in water at various pH levels.

Compound	Absorption Max., nm of Neutral Form	Absorption Max., nm of Protonated Form	FQY, % in Acidic Media	p*K*_a_ Based on Absorbance Titration Data ^a^	p*K*_a_ Based on Emission Titration Data ^a^
**3**	552	475	14	4.0	3.9
**4**	522	480	92	7.7	7.5
**7a**	591	539	2.0	6.8	7.0
**7b**	601	558/578 ^b^	1.0	7.4/4.2 ^b^	7.0/4.4 ^b^
**7c**	596	546	2.8	7.5	7.5
**9**	608	553	27	7.5	7.1

^a^—5 mM phosphate buffer was used and 10% of EtOH was added due to the low solubility; ^b^—the doubly protonated form.

## Data Availability

Data are contained within the article or Supplementary Materials.

## Supplementary Materials

The following supporting information can be downloaded at https://www.mdpi.com/article/10.3390/ijms251910448/s1.
