# Supplementary material for: Developing 1,4-Diethyl-1,2,3,4-tetrahydroquinoxalin-substituted Fluorogens Based on GFP Chromophore for Endoplasmic Reticulum and Lysosome Staining
Source: Int J Mol Sci. 2024 Sep 27;25(19):10448. doi: 10.3390/ijms251910448 (PMC11477126; doi:10.3390/ijms251910448)
Supplement: Supplementary file 1 [file ijms-25-10448-s001.zip › ijms-3214205-supplementary.pdf]

Contents

**1. Absorption and emission spectra**	**S2**
**2. pH-titration**	**S7**
**3. Fluorescent microscopy**	**S19**
**4. Photobleaching**	**S20**
**5. Relative fluorescence quantum yields in water-glycerol mixtures**	**S21**
**6. Fluorescence lifetime measurements in vitro**	**S22**
**7. Synthesis results**	**S23**
**8. References**	**S30**
**9. Copies of ^{1}H and ^{13}C NMR spectra**	**S31**

1. Absorption and emission spectra

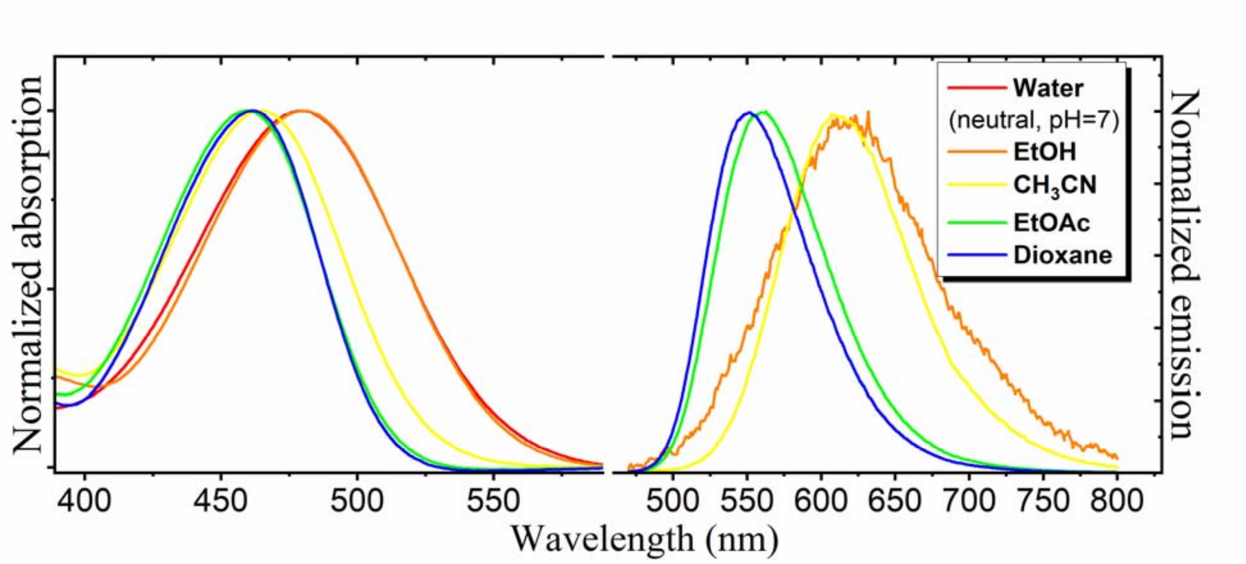

Figure S1.1. Normalized absorption and emission spectra of compound **2**

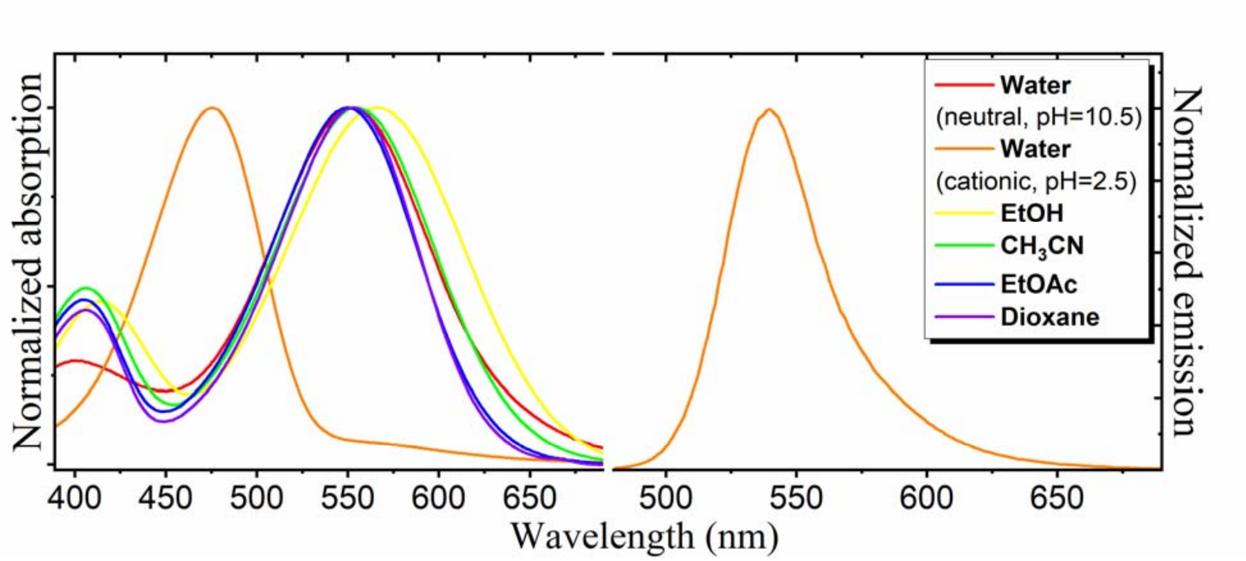

Figure S1.2. Normalized absorption and emission spectra of compound **3**

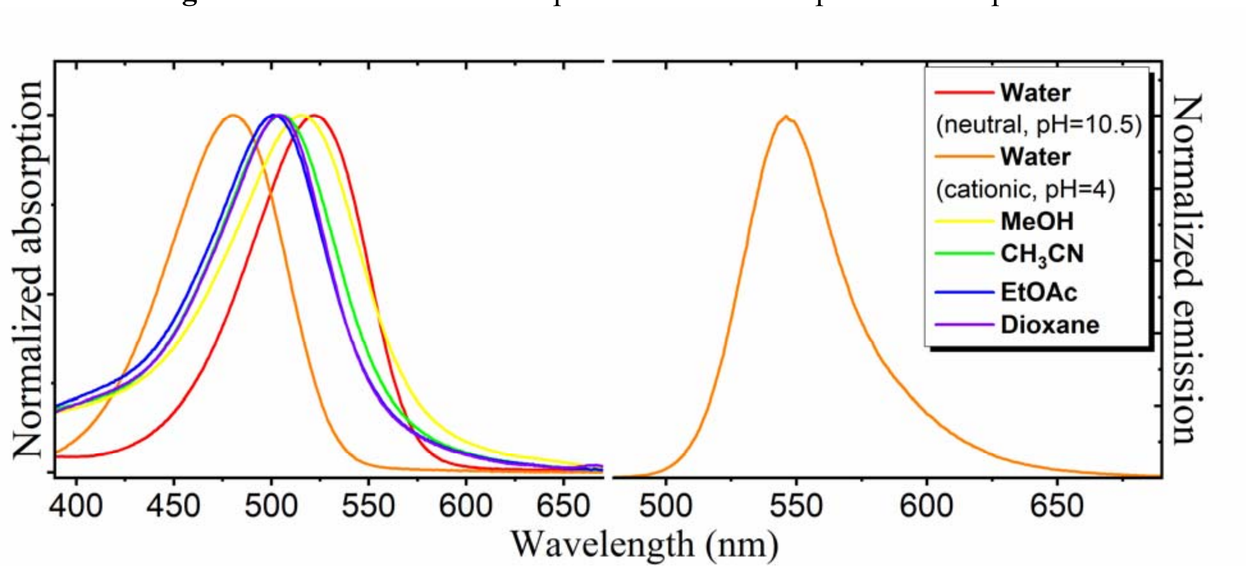

Figure S1.3. Normalized absorption and emission spectra of compound **4**

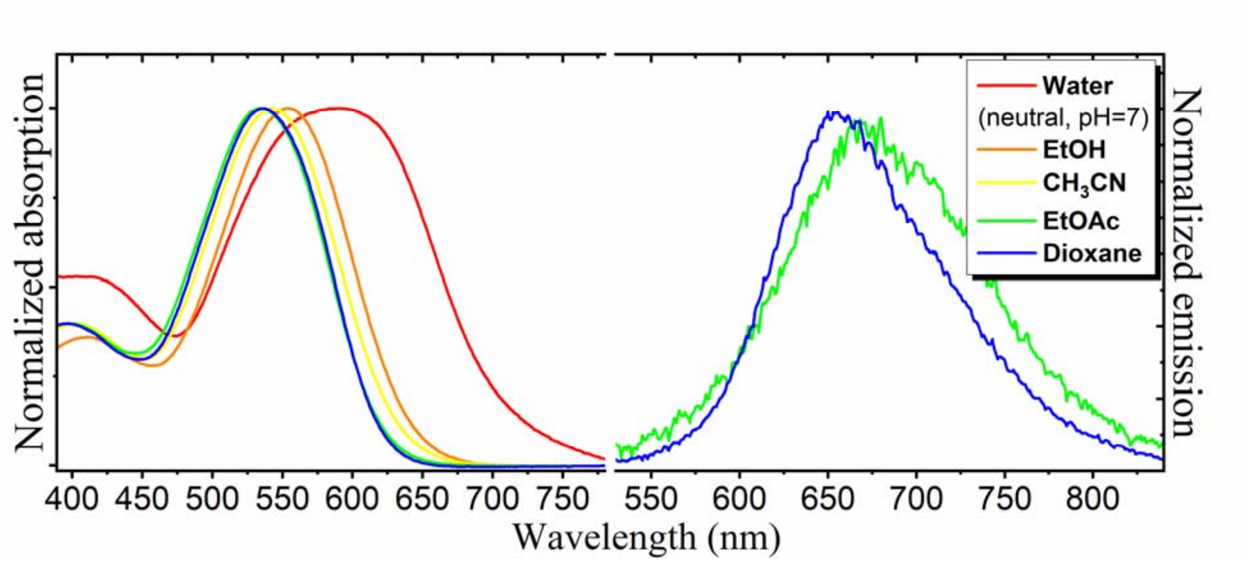

Figure S1.4. Normalized absorption and emission spectra of compound **5a**

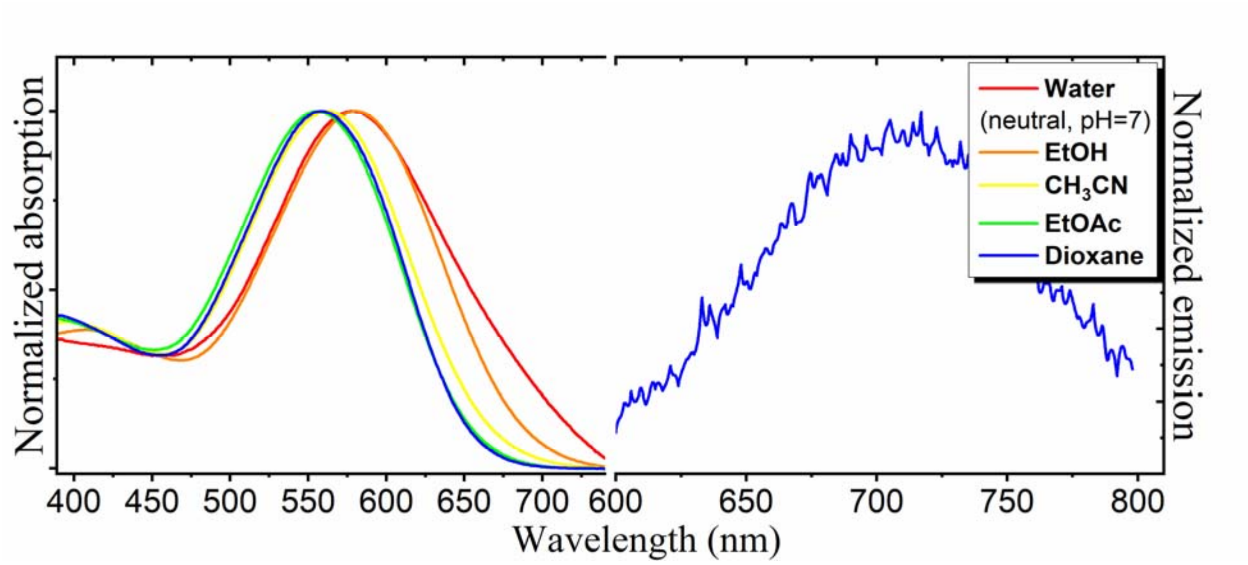

Figure S1.5. Normalized absorption and emission spectra of compound **5b**

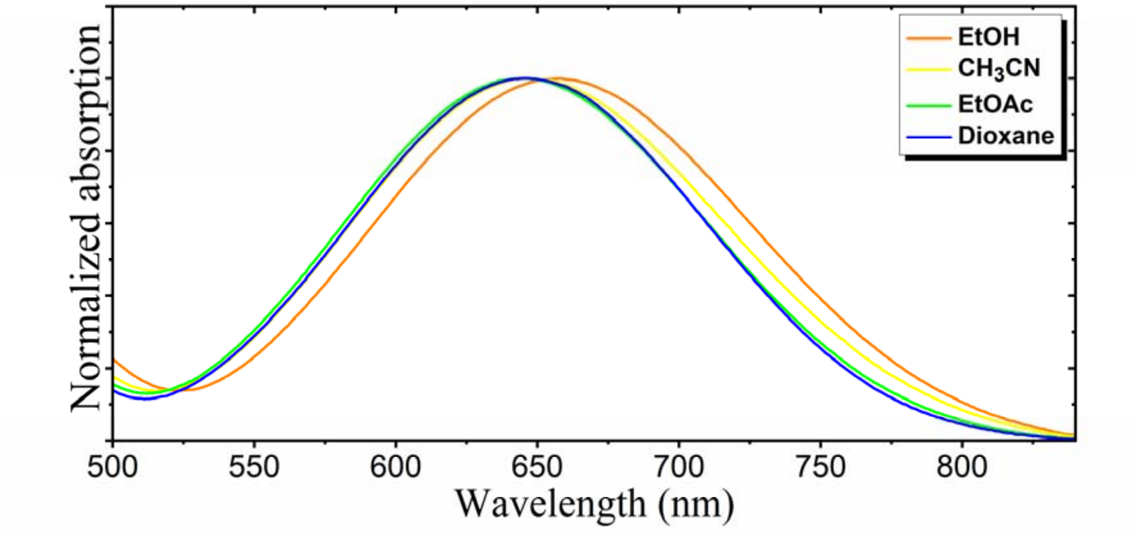

Figure S1.6. Normalized absorption and emission spectra of compound **6a**

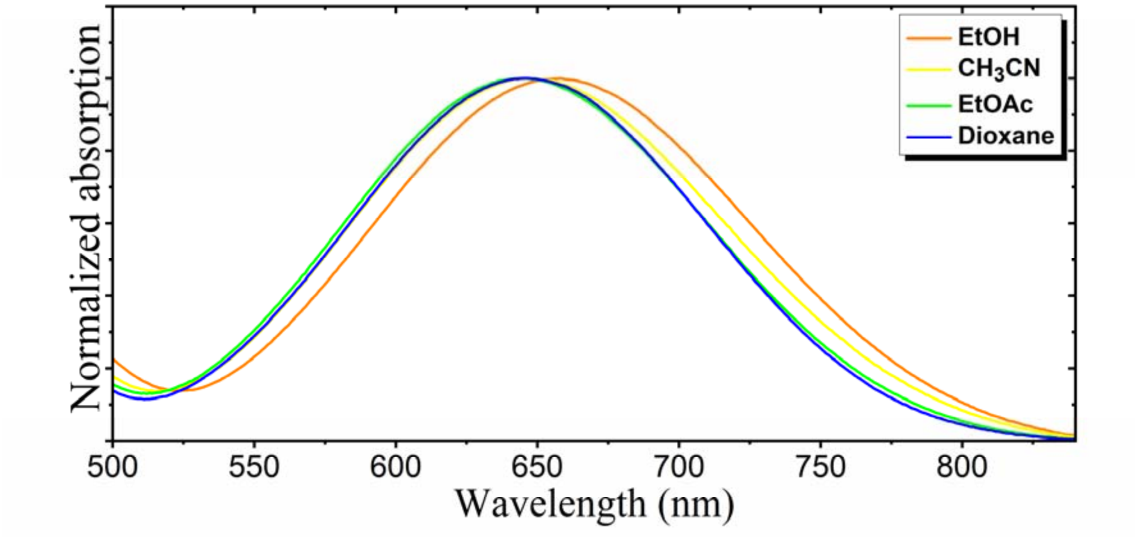

Figure S1.7. Normalized absorption and emission spectra of compound **6c**

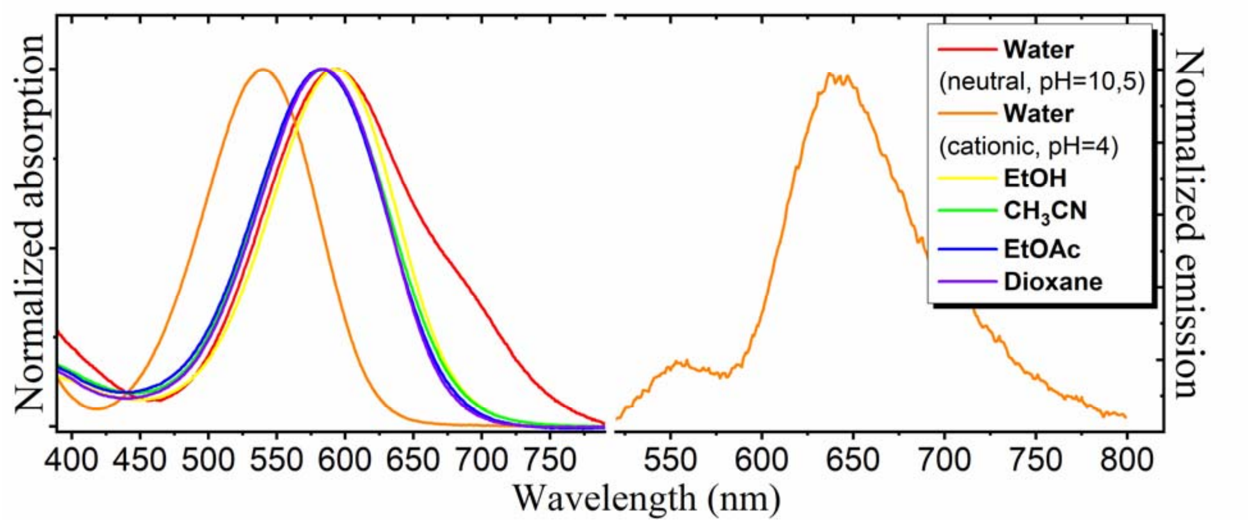

Figure S1.8. Normalized absorption and emission spectra of compound **7a**

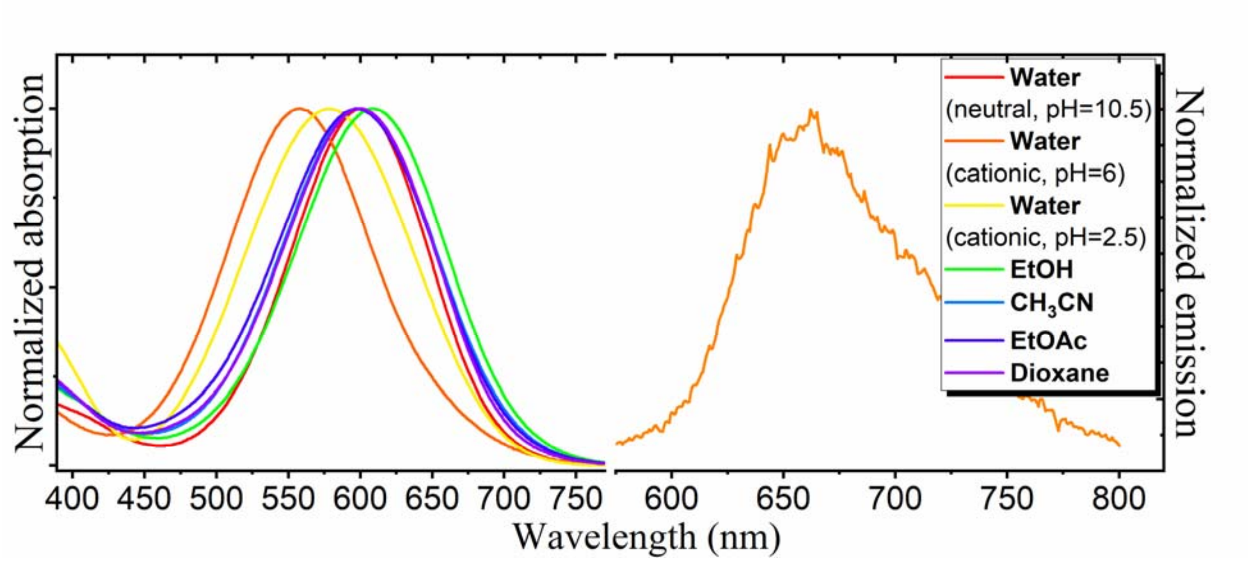

Figure S1.9. Normalized absorption and emission spectra of compound **7b**

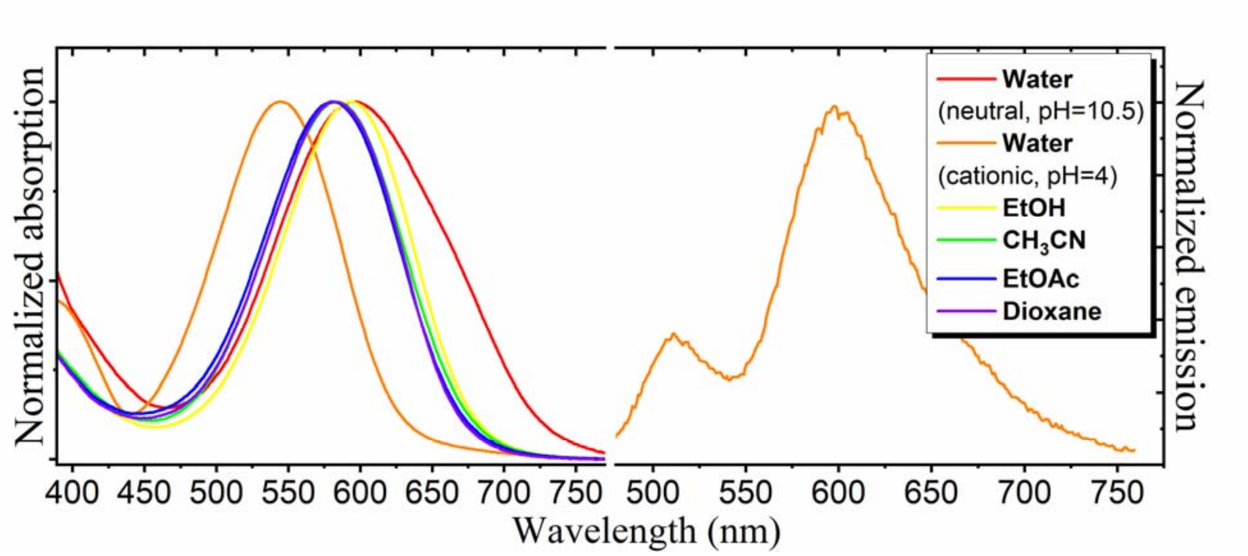

Figure S1.10. Normalized absorption and emission spectra of compound **7c**

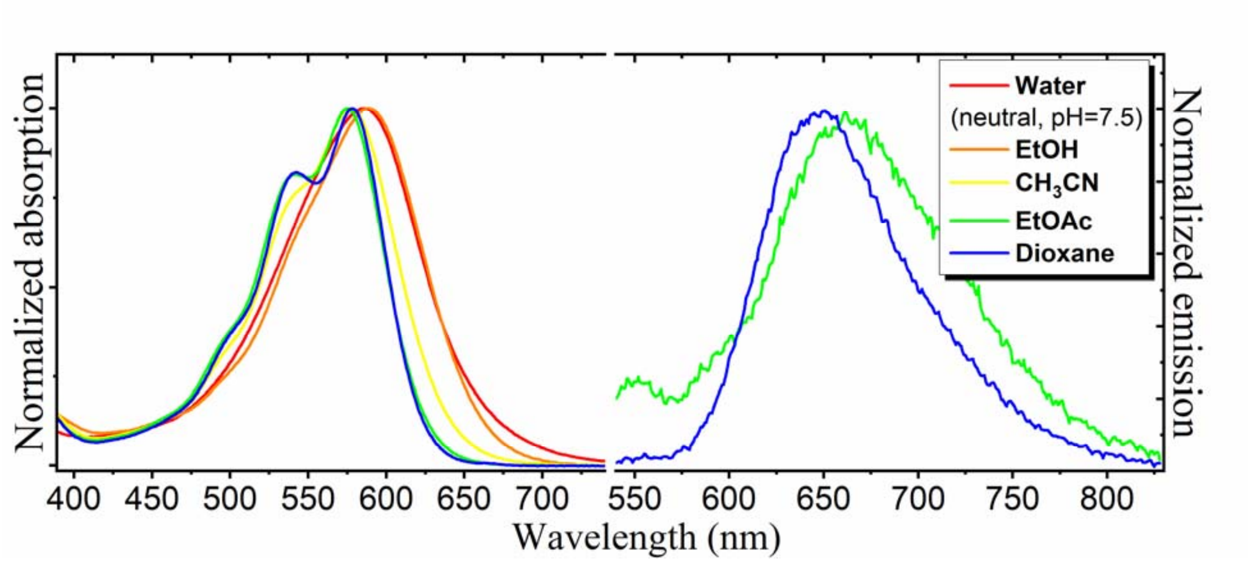

Figure S1.11. Normalized absorption and emission spectra of compound **8**

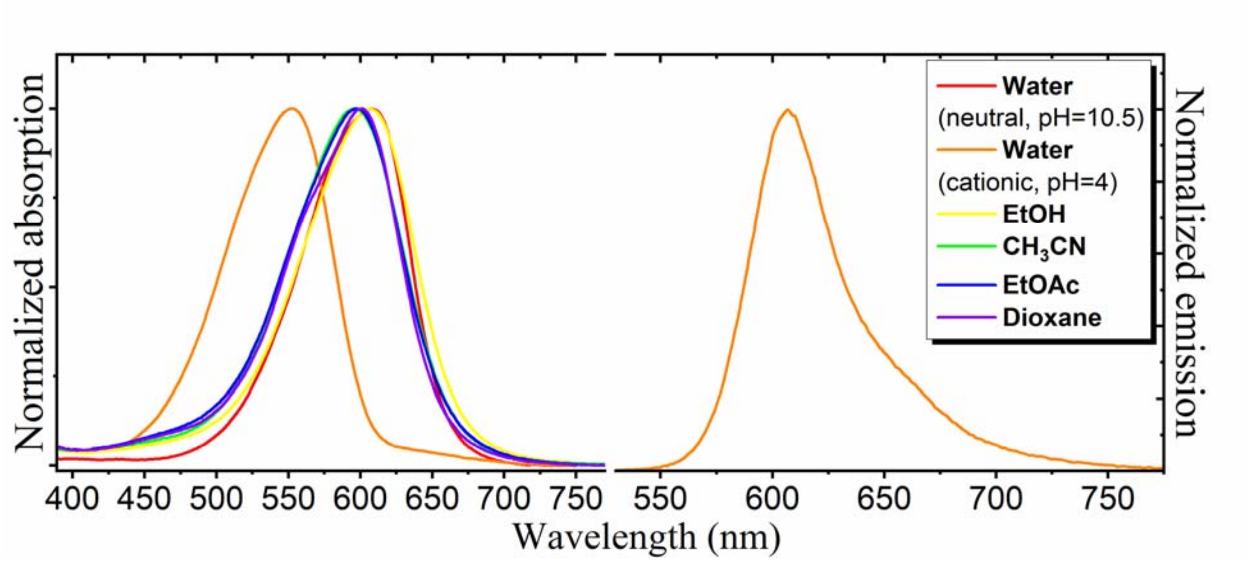

Figure S1.12. Normalized absorption and emission spectra of compound **9**

2. pH-titration

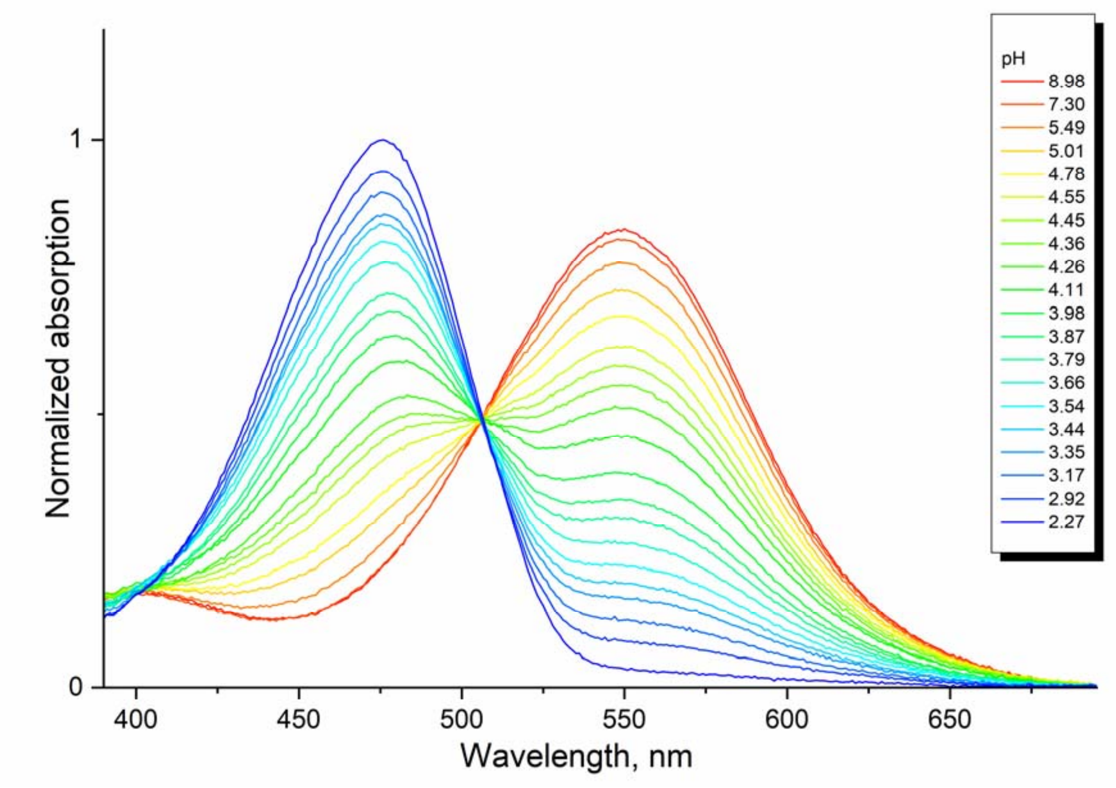

Figure S2.1. Normalized absorption spectra of compound **3** at various pH

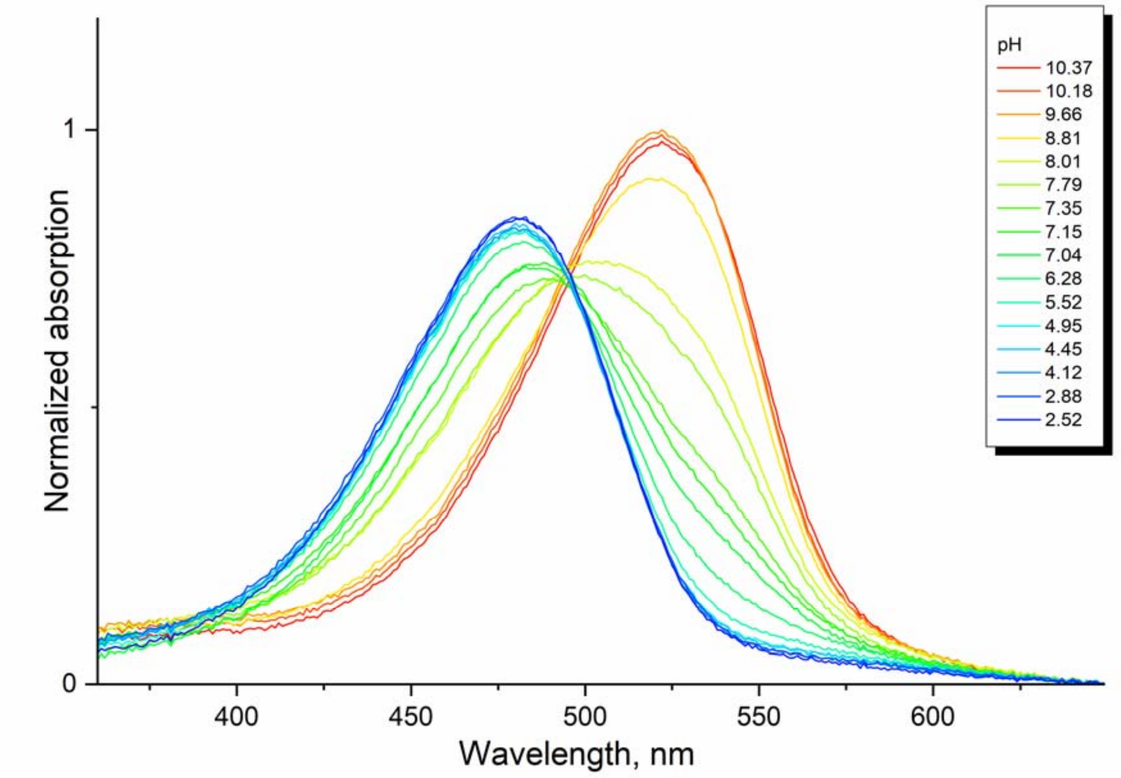

Figure S2.2. Normalized absorption spectra of compound **4** at various pH

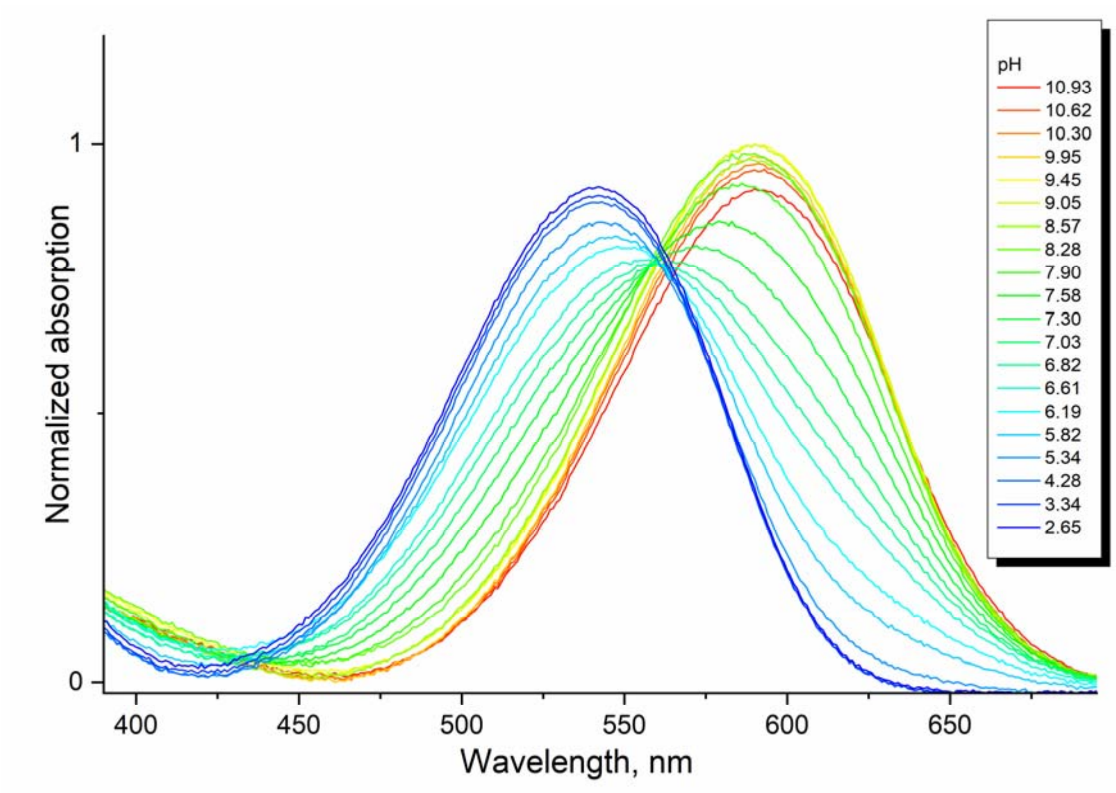

Figure S2.3. Normalized absorption spectra of compound **7a** at various pH

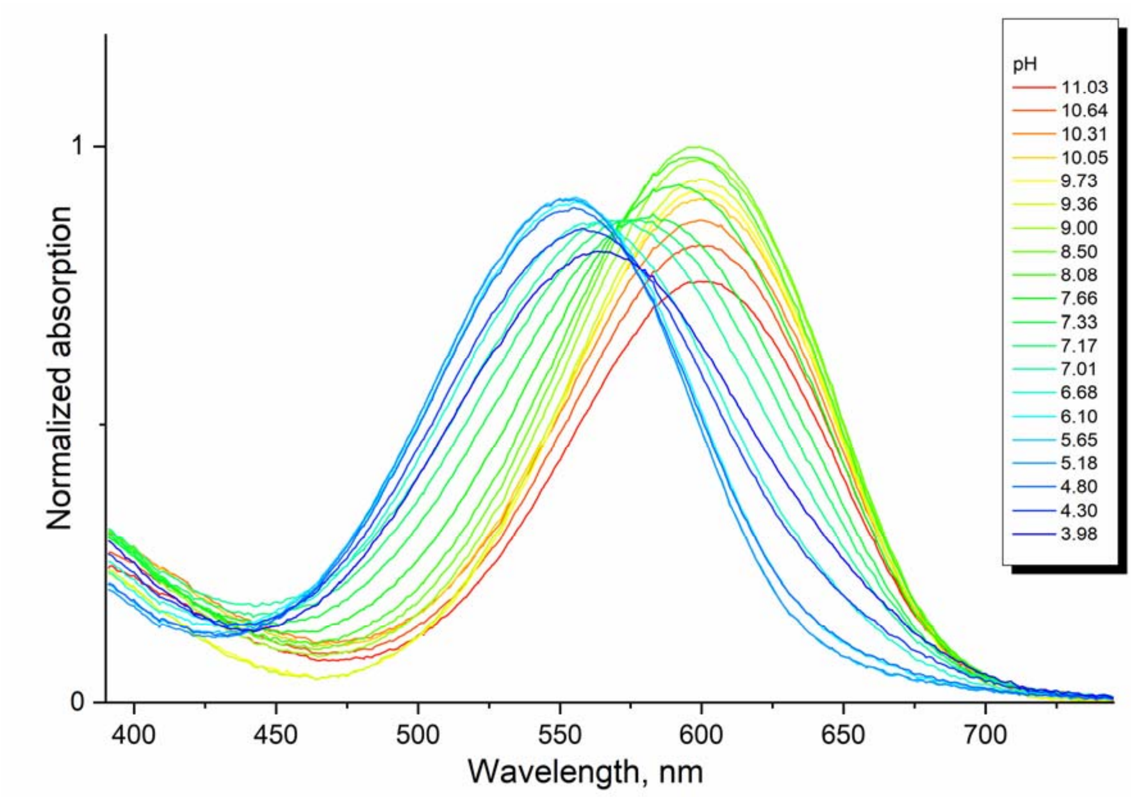

Figure S2.4. Normalized absorption spectra of compound **7b** at various pH

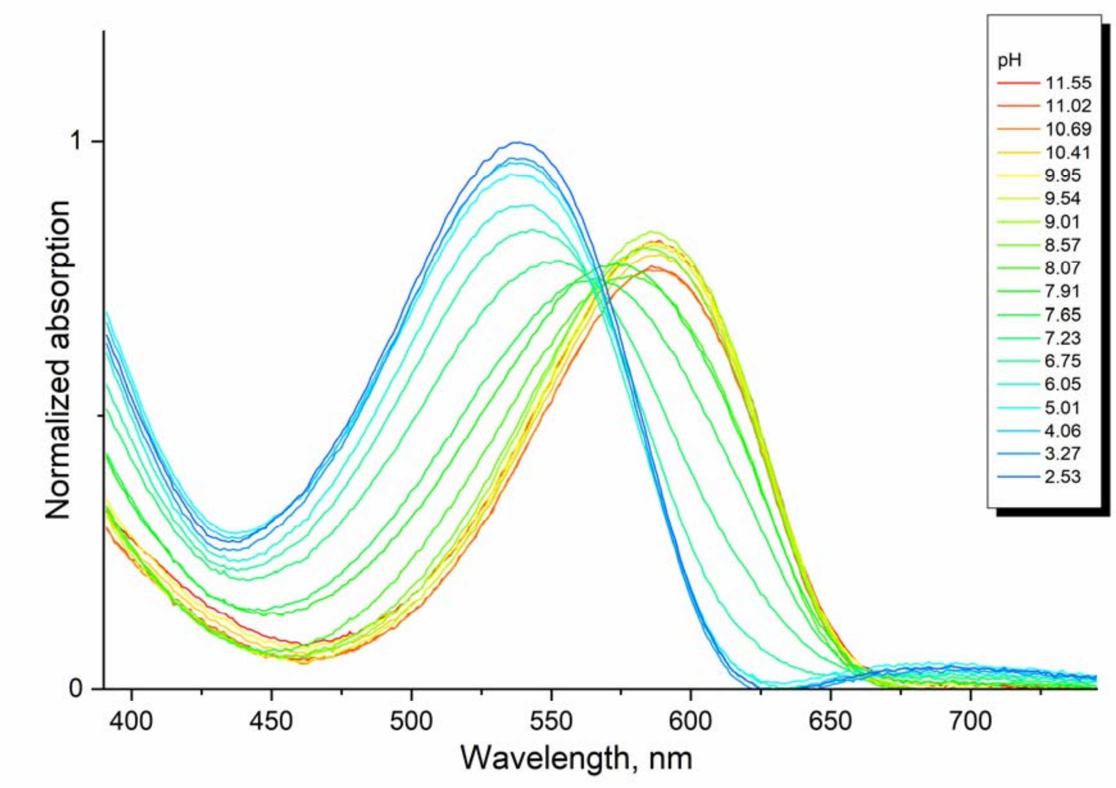

Figure S2.5. Normalized absorption spectra of compound **7c** at various pH

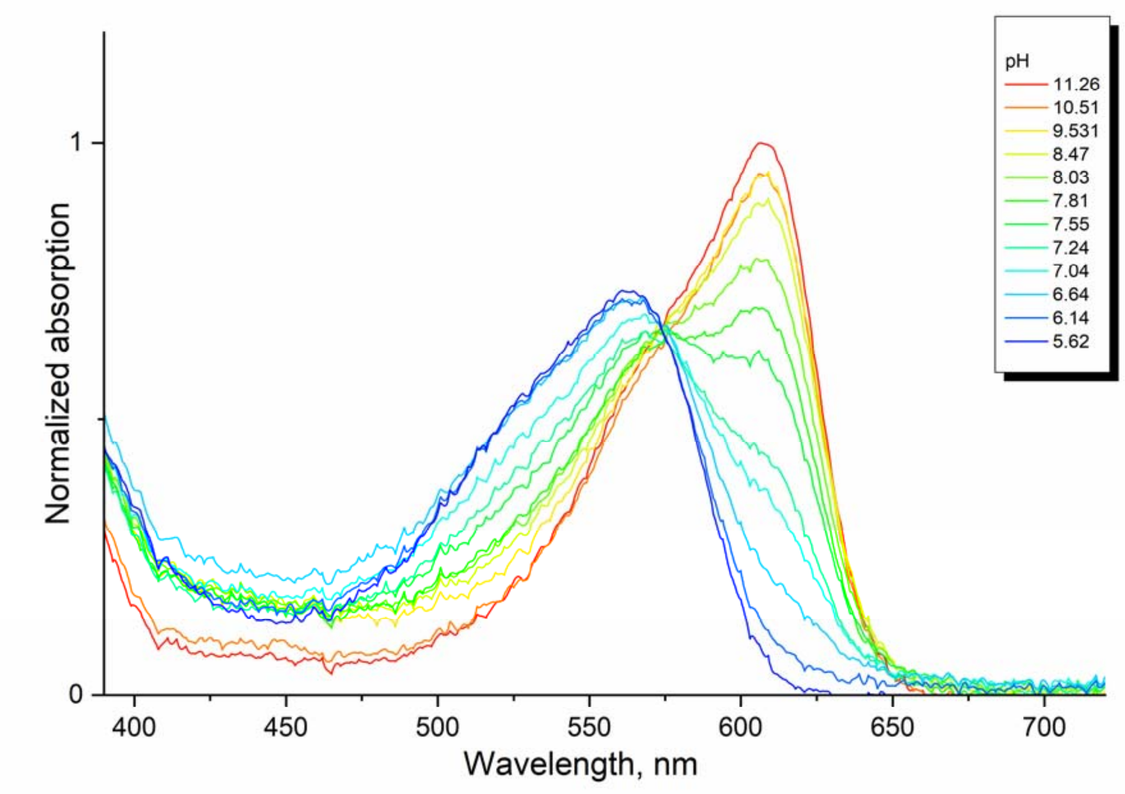

Figure S2.6. Normalized absorption spectra of compound **9** at various pH

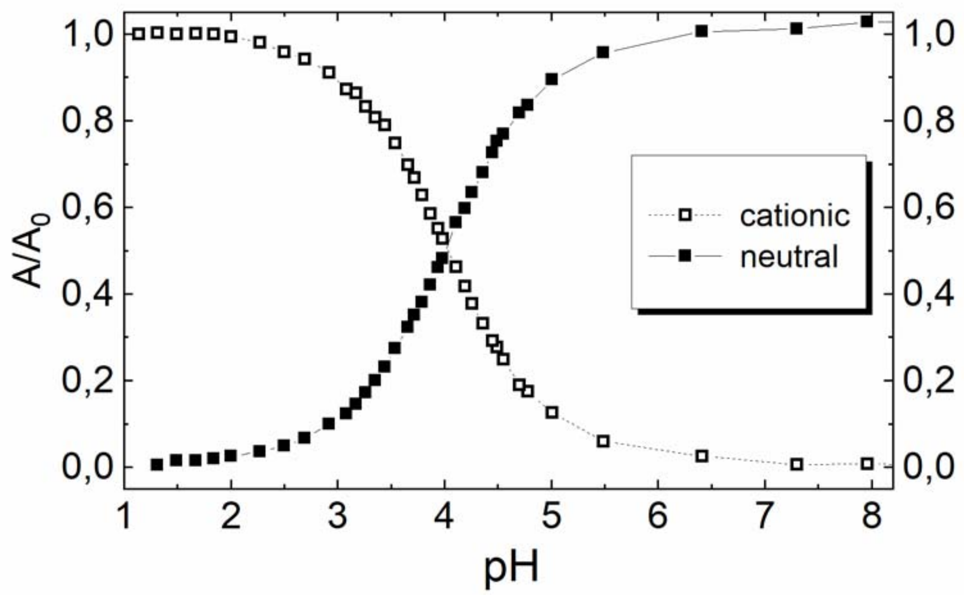

Figure S2.7. Titration curves based on absorption spectra of compound **3** at 460/560 nm

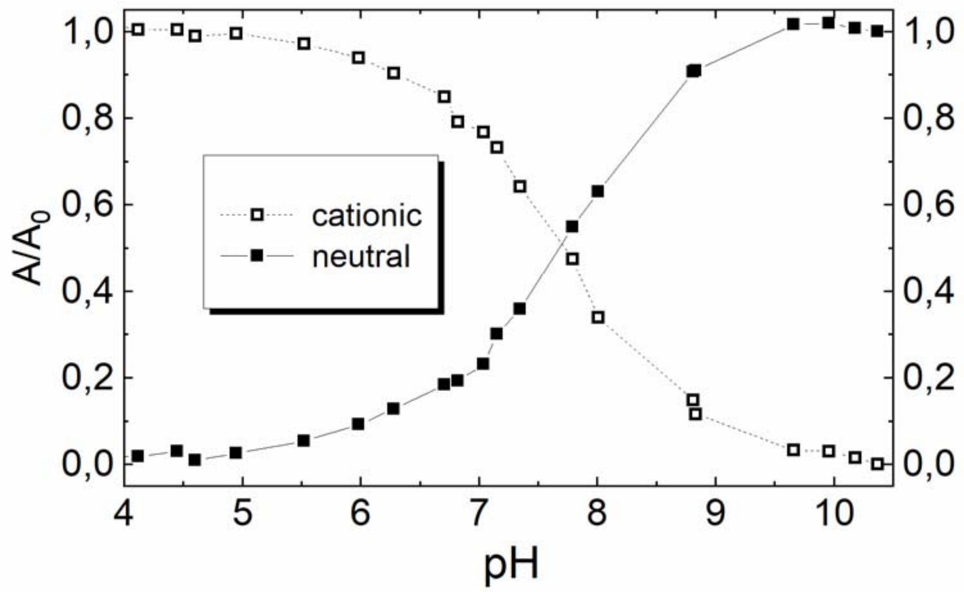

Figure S2.8. Titration curves based on absorption spectra of compound **4** at 460/540 nm

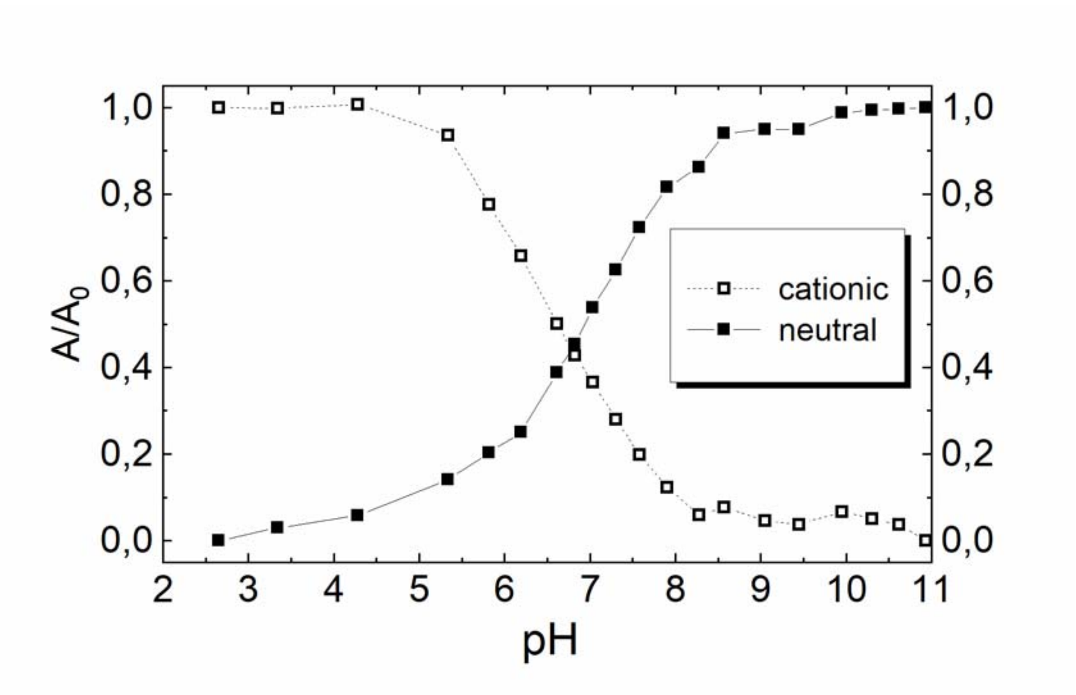

Figure S2.9. Titration curves based on absorption spectra of compound **7a** at 500\650 nm

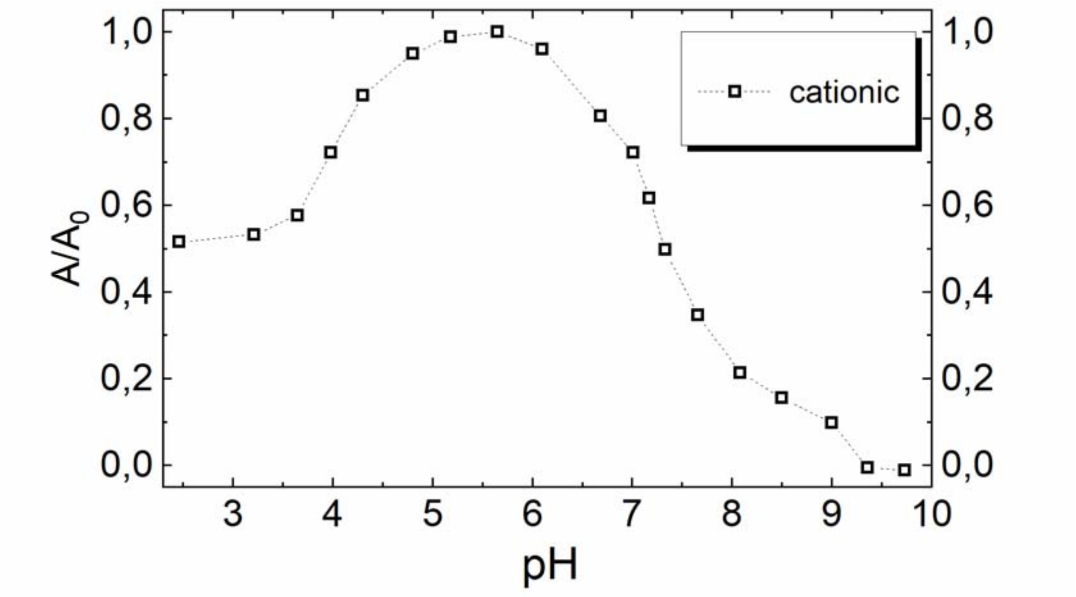

Figure S2.10. Titration curve based on absorption spectra of compound **7b** at 500 nm

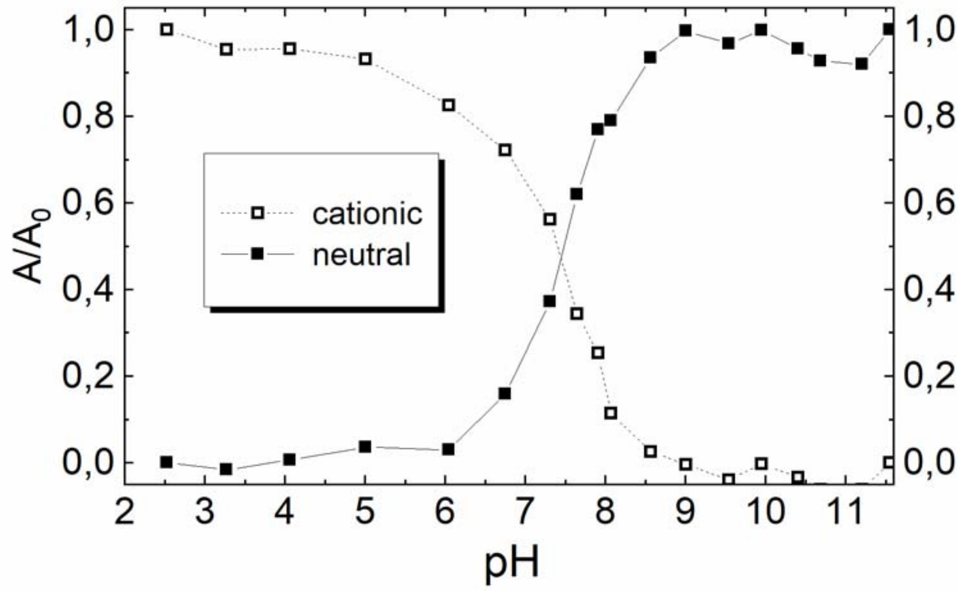

Figure S2.11. Titration curves based on absorption spectra of compound **7c** at 500/625 nm

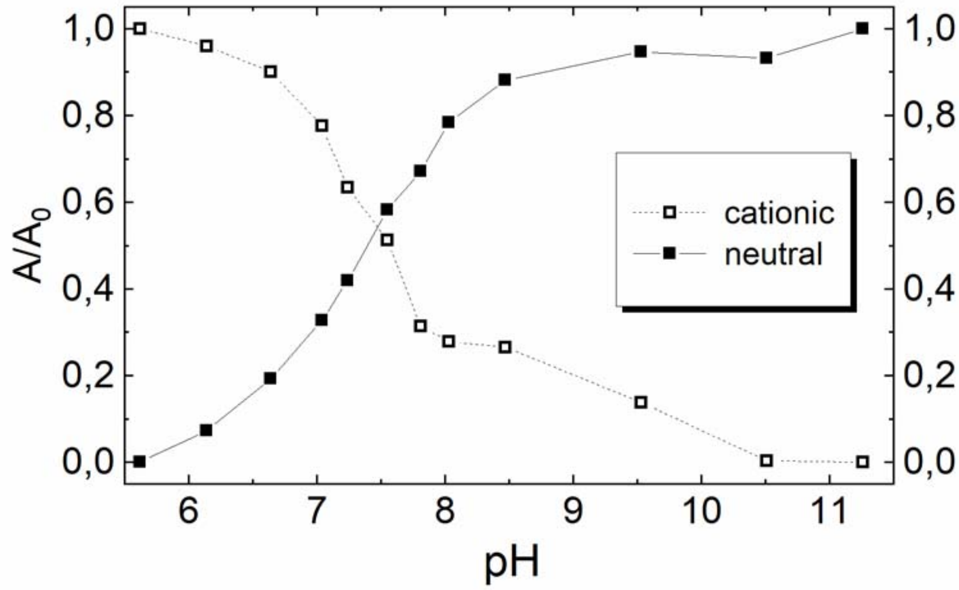

Figure S2.12. Titration curves based on absorption spectra of compound **9** at 520/620 nm

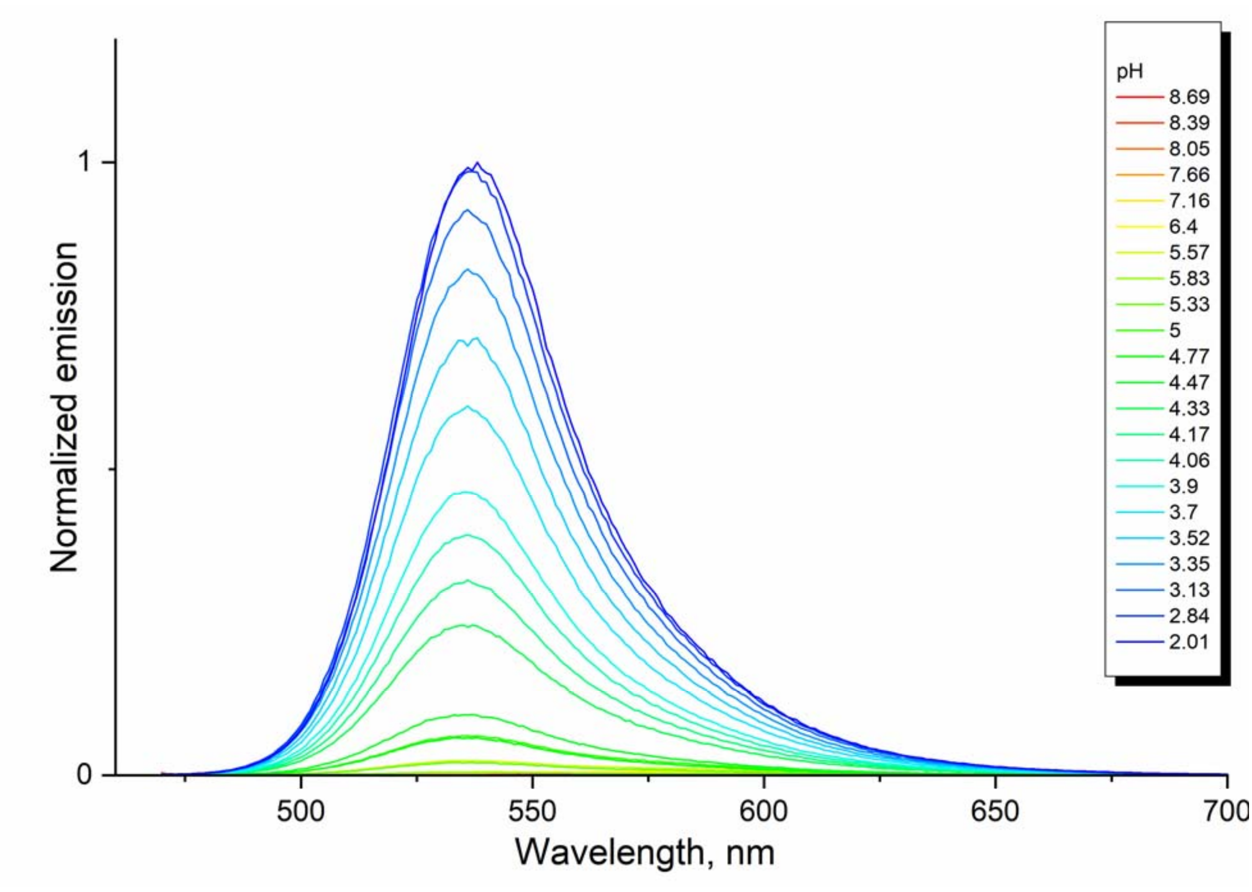

Figure S2.13. Normalized emission spectra of compound **3** at various pH, excitation at 460 nm

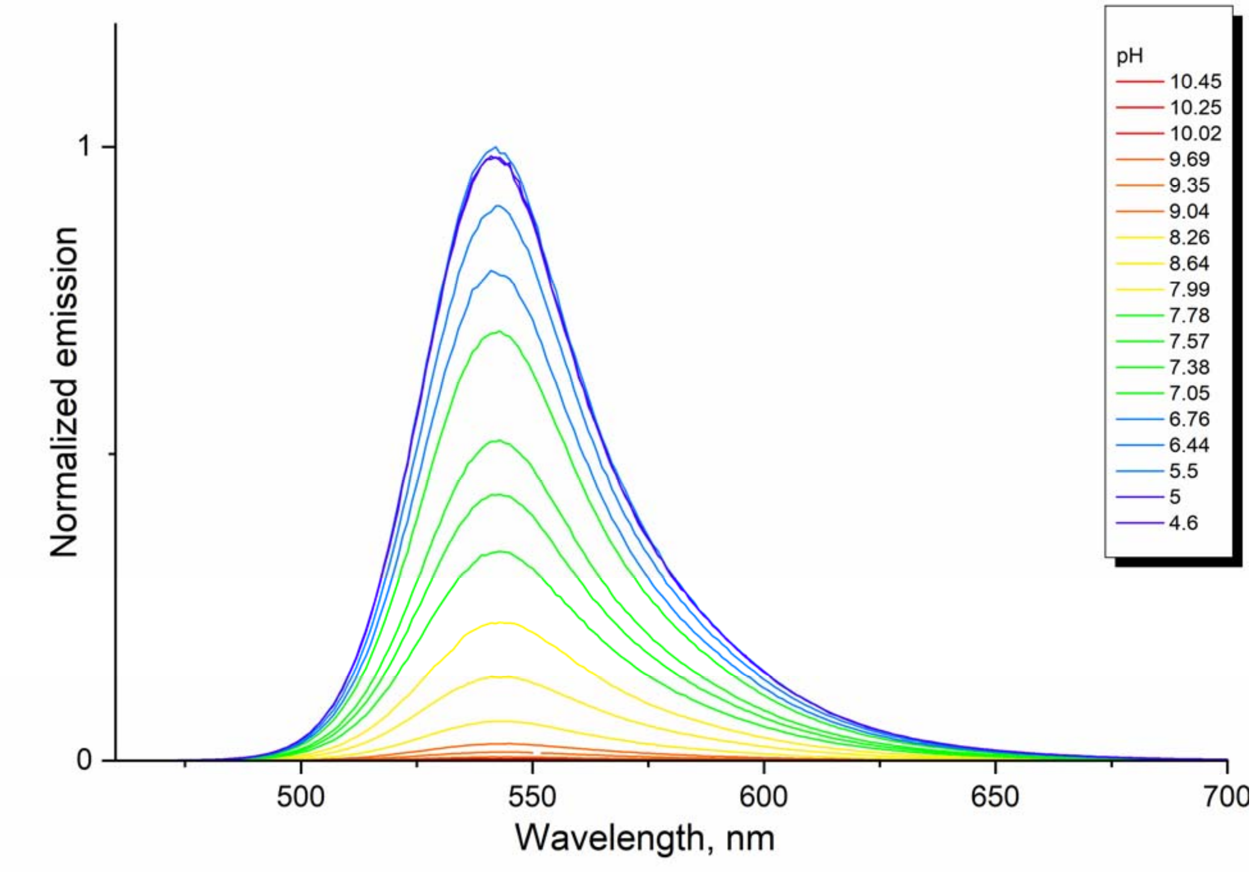

Figure S2.14. Normalized emission spectra of compound **4** at various pH, excitation at 460 nm

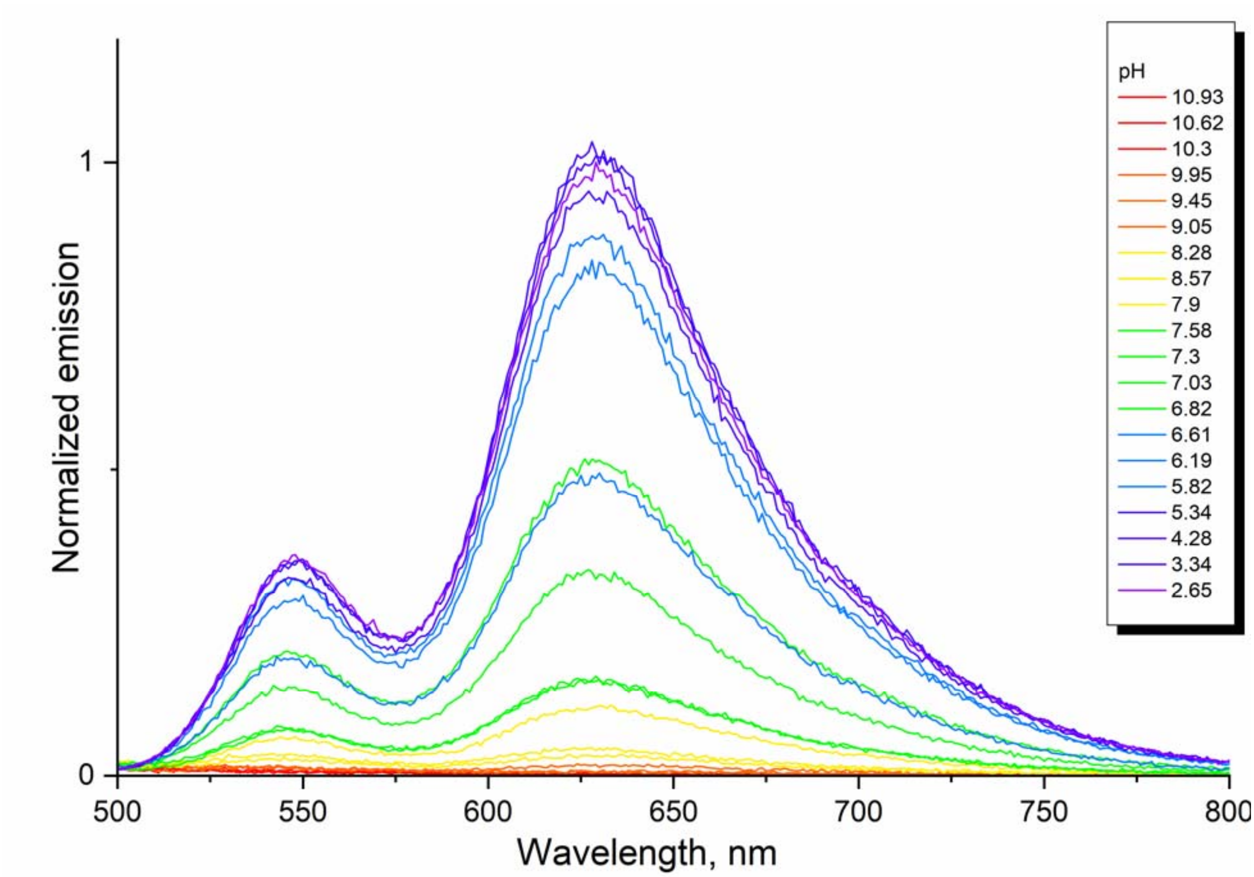

Figure S2.15. Normalized emission spectra of compound **7a** at various pH, excitation at 480 nm

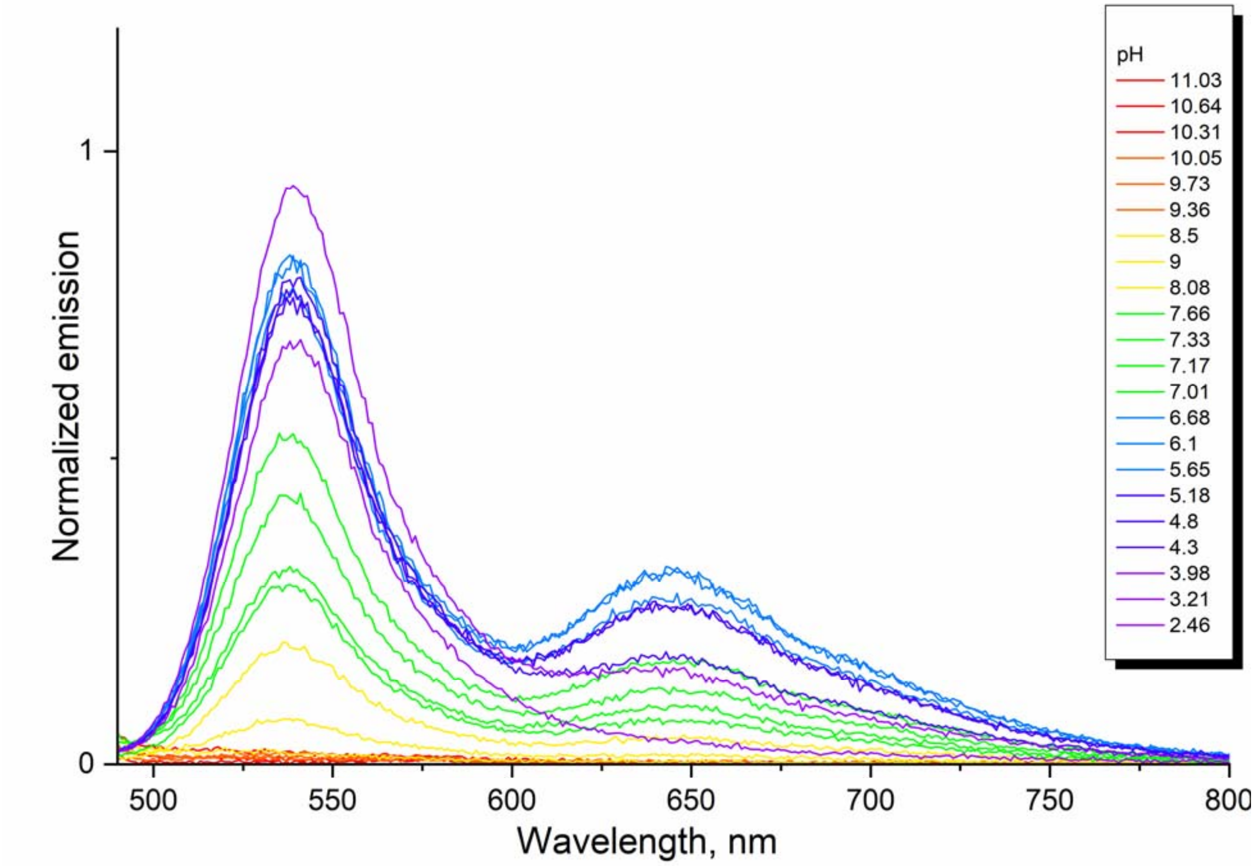

Figure S2.16. Normalized emission spectra of compound **7b** at various pH, excitation at 480 nm

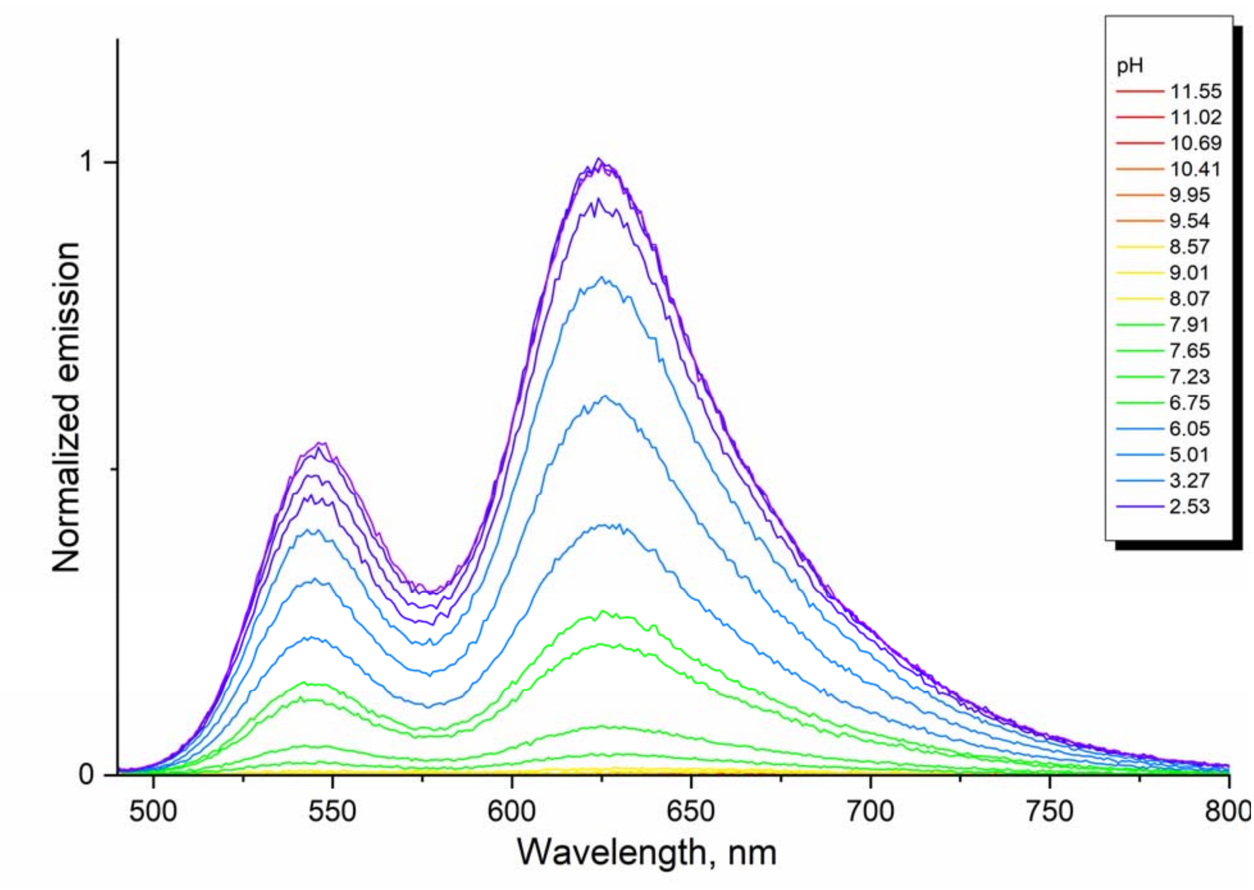

Figure S2.17. Normalized emission spectra of compound **7c** at various pH, excitation at 490 nm

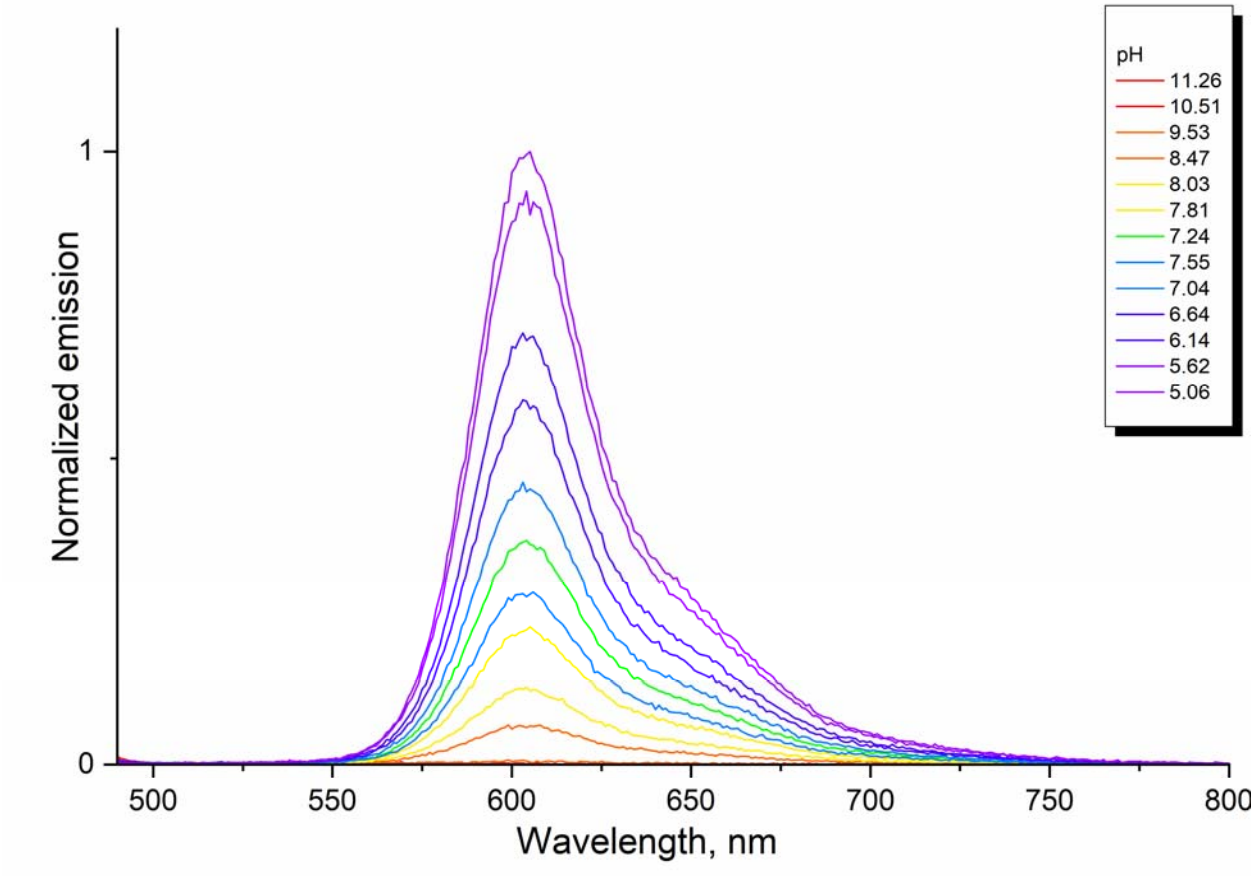

Figure S2.18. Normalized emission spectra of compound **9** at various pH, excitation at 480 nm

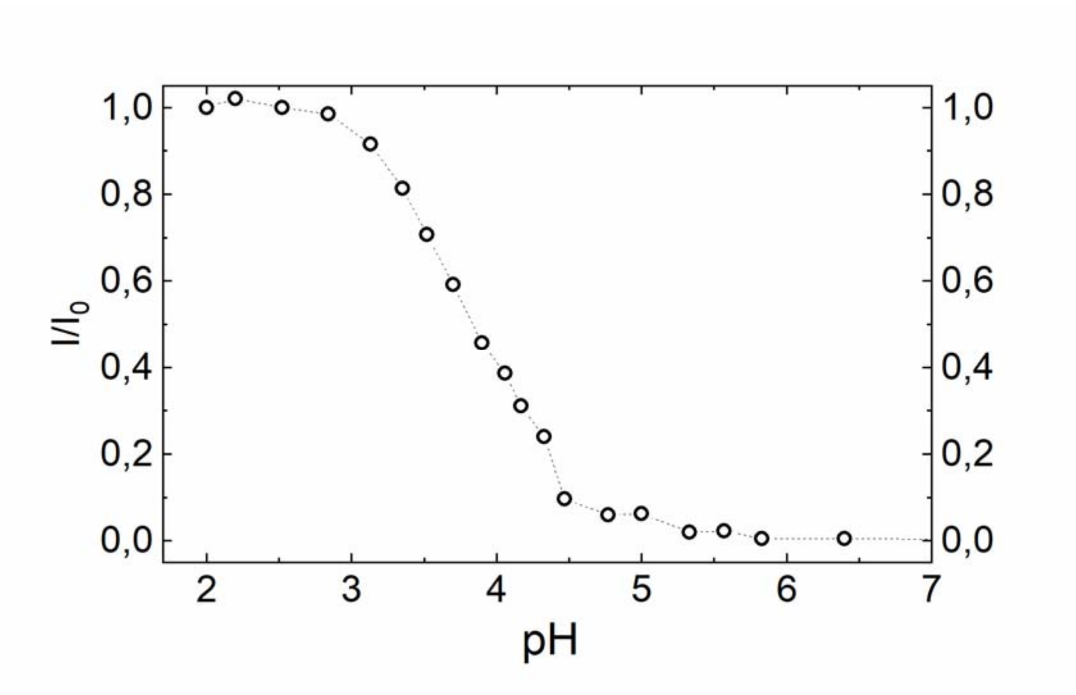

Figure S2.19. Titration curve based on emission spectra of compound **3** at 560 nm

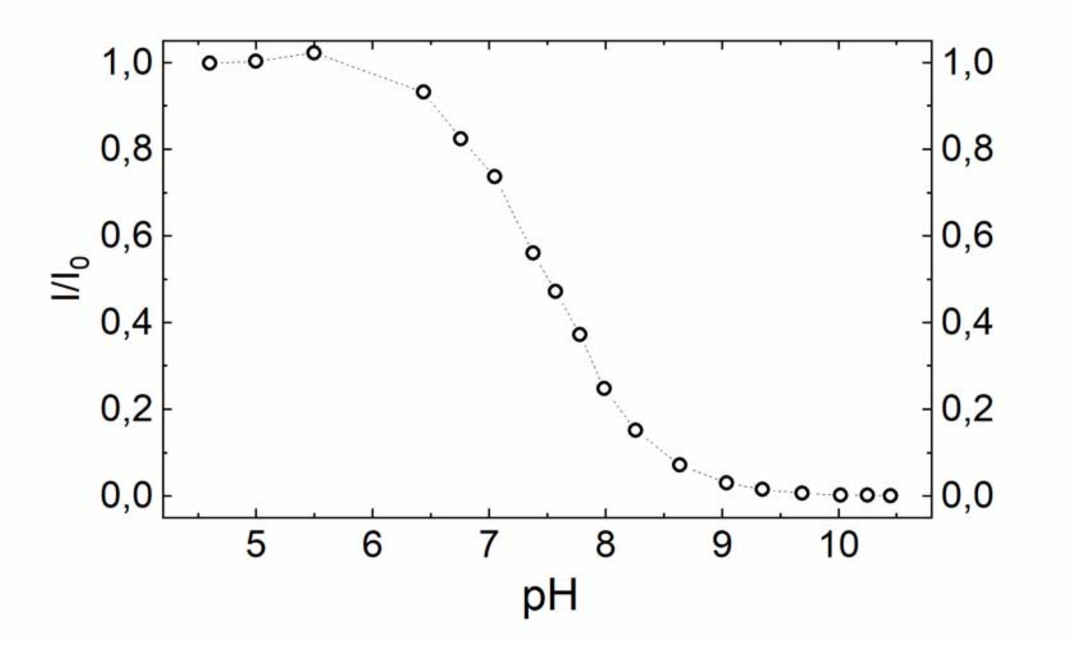

Figure S2.20. Titration curve based on emission spectra of compound **4** at 560 nm

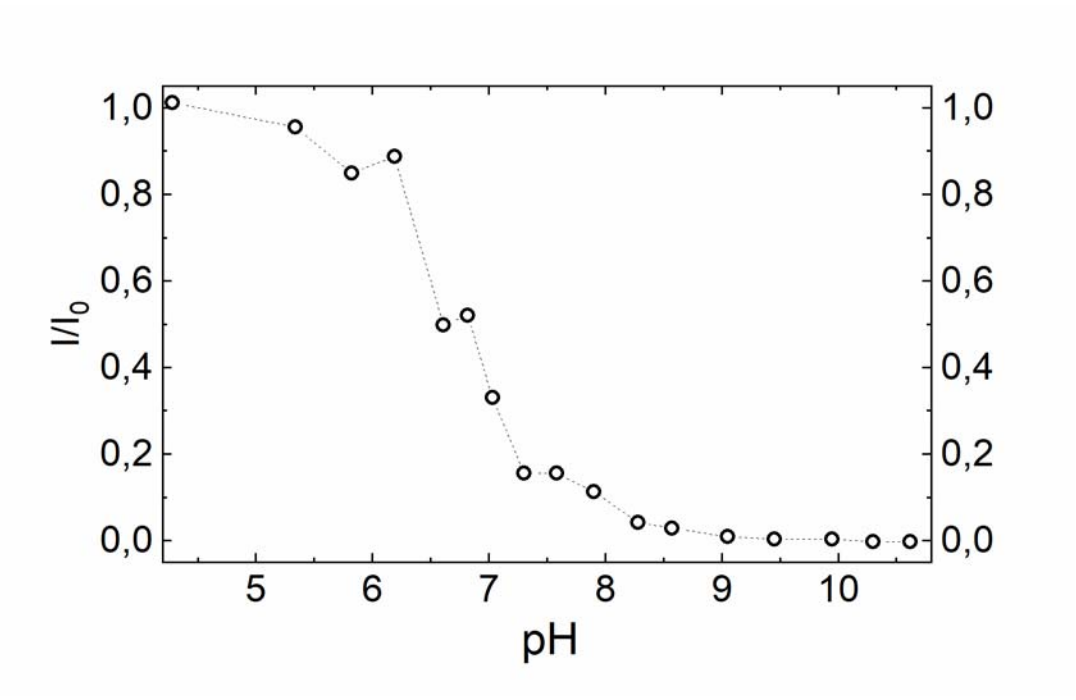

Figure S2.21. Titration curve based on emission spectra of compound **7a** at 630 nm

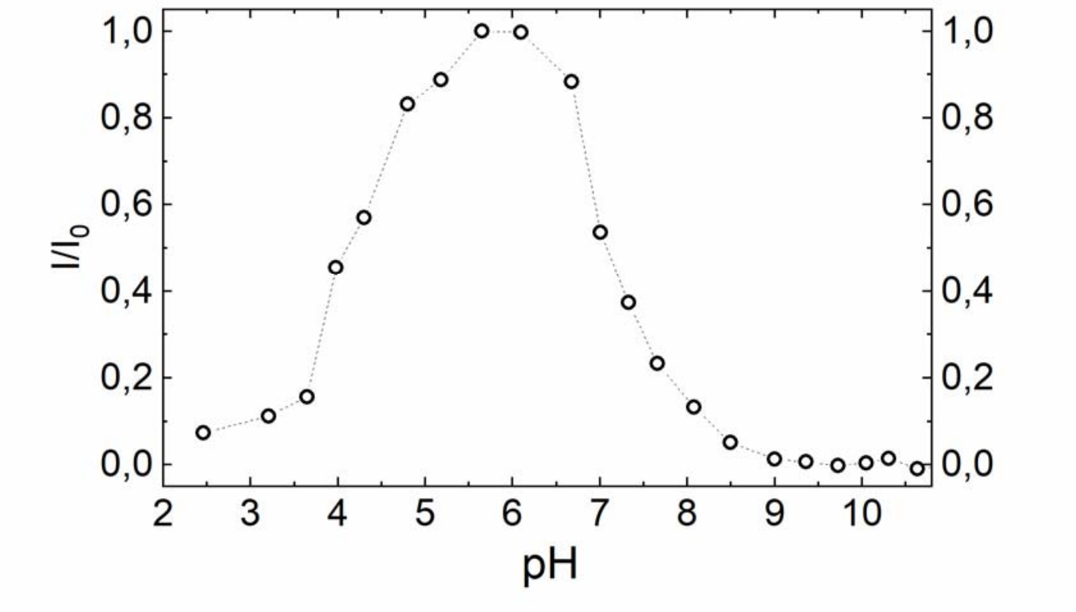

Figure S2.22. Titration curve based on emission spectra of compound **7b** at 660 nm

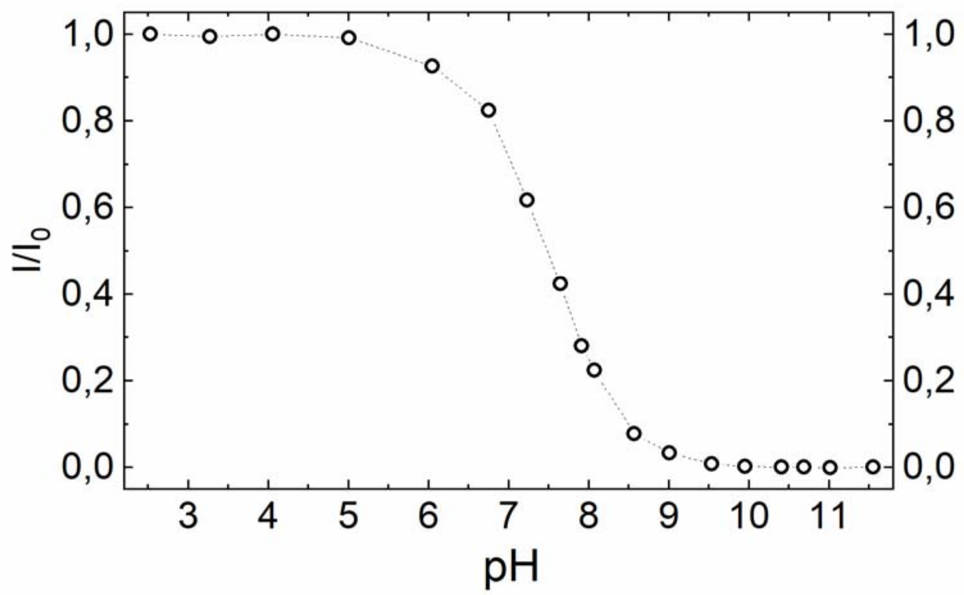

Figure S2.23. Titration curve based on emission spectra of compound **7c** at 640 nm

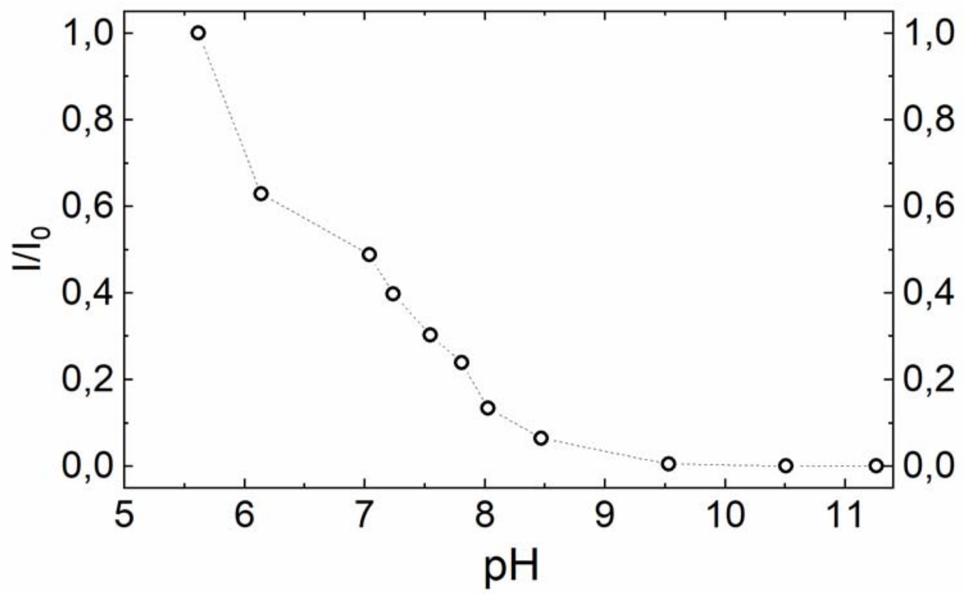

Figure S2.24. Titration curve based on emission spectra of compound **9** at 600 nm

3. Fluorescent microscopy

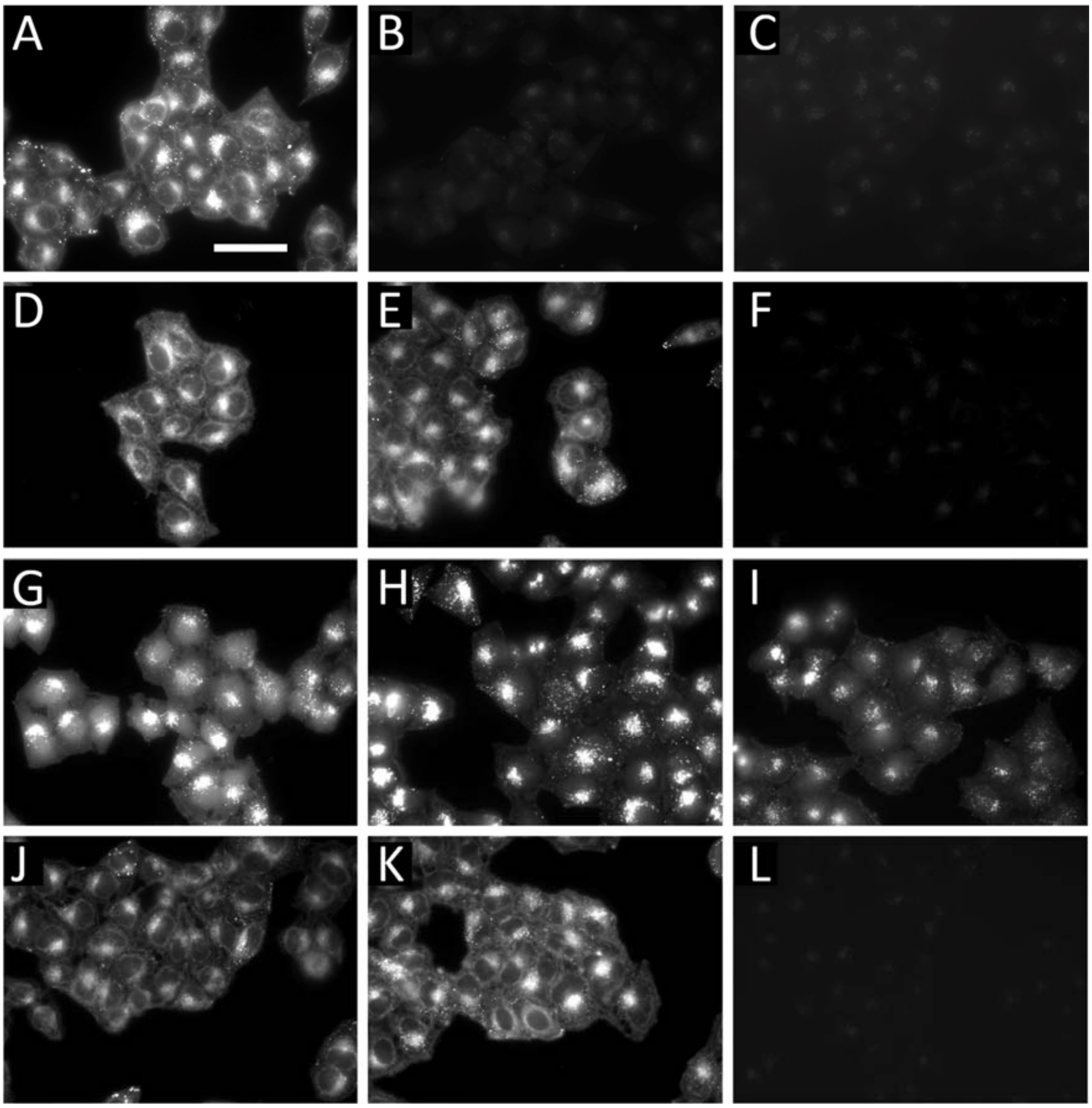

Figure S3.1. Testing of fluorogenic dyes in live-cell imaging of HeLa Kyoto cells using widefield microscopy. Scale bar is 50 μm. (A) **2**, GFP filter set, 5 μM. (B) **3**, GFP filter set, 10 μM. (C) **4**, GFP filter set, 10 μM. (D) **5b**, TxRed filter set, 5 μM. (E) **5a**, TRITC filter set, 1 μM. (F) **6a**, TxRed filter set, 10 μM. G) **7b**, TRITC filter set, 1 μM. (H) **7a**, TxRed filter set, 5 μM. (I) **7c**, TxRed filter set, 5 μM. (J) **6c**, TxRed filter set, 10 μM. (K) **8**, TRITC filter set, 5 μM. (L) **9**, TxRed filter set, 5 μM.

4. Photobleaching

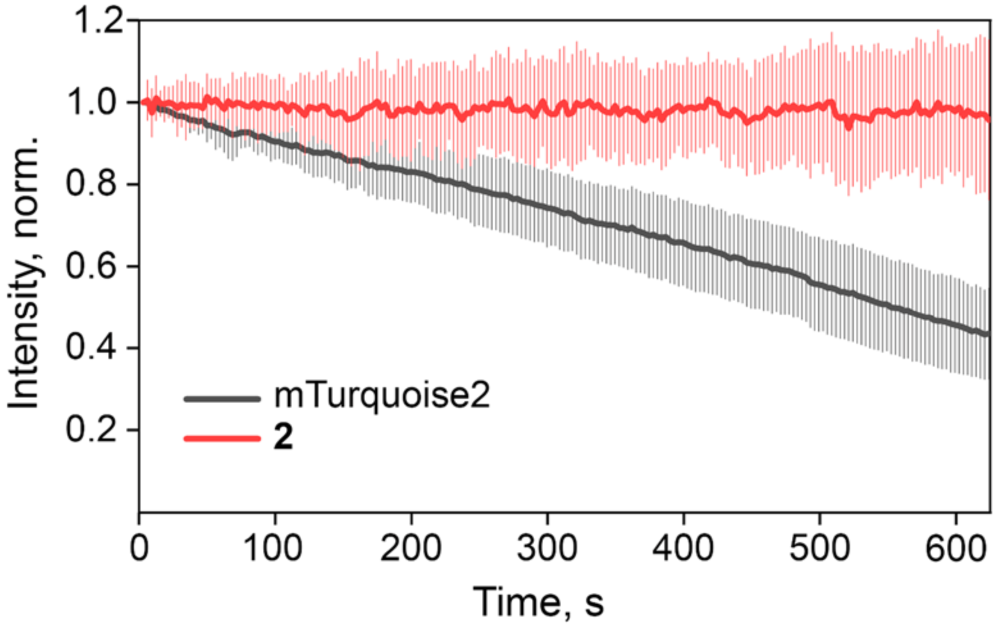

Figure S4.1 Photostability analysis in HeLa Kyoto cells stained with 5 µM of **2** dye in comparison with ER-localized fluorescent protein (mTurquoise2). The time-lapse regime with 6,1 µW of the 458 nm laser line was used. Data are shown as mean ± SD.

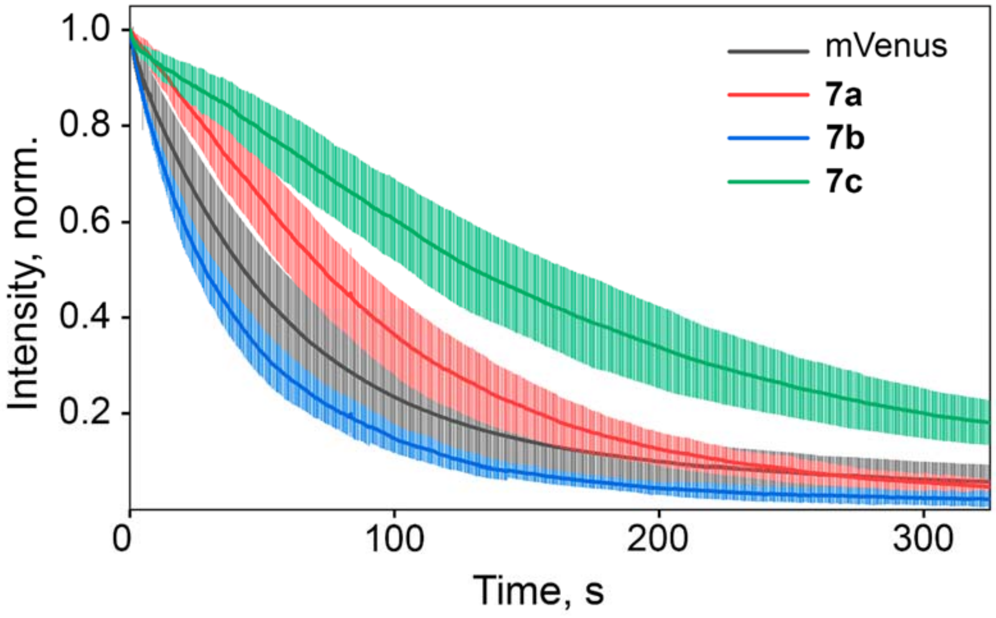

Figure S4.2 Photostability analysis in HeLa Kyoto cells stained with 5 µM of **7a**, **7c** or 1 µM of **7b** dyes in comparison with mVenus. The time-lapse regime with 8,7 µW of the 514 nm laser line was used. Data are shown as mean ± SD.

5. Relative fluorescence quantum yields in water-glycerol mixtures

Table 5.1. Relative fluorescence quantum yields of compounds **7a-c** in water with glycerol addition.

Compound	Water-Glycerol (v/v)			
	1:0	2:1	1:1	1:2
**7a** (pH=4)	1	1.67	2.27	2.78
**7b** (pH=2)	1	1.43	1.71	1.48
**7b** (pH=6)	1	1.38	1.70	2.23
**7c** (pH=4)	1	1.28	1.62	2.05

6. Fluorescence lifetime measurements in vitro

Table S6.1. Fluorescence lifetimes (τ) of **7a** in water solution with different pH values. τ is the fluorescence lifetime of the corresponding exponential component; A is a relative contribution of the exponential decay component; τ_m is an amplitude weighted lifetime; χ2 is the Pearson criterion. SD is standard deviation.

pH value	$\tau_1 \pm$ SD, ns	A_1, %	$\tau_2 \pm$ SD, ns	A_2, %	τ_m, ns	χ^2
3	0.115 ± 0.005	59	0.655 ± 0.012	41	0.336	1.192
4	0.186 ± 0.007	51	0.651 ± 0.018	49	0.414	1.269
5	0.113 ± 0.007	52	0.711 ± 0.090	48	0.400	1.272
6	0.136 ± 0.006	38	0.776 ± 0.075	62	0.533	1.307
7	0.100 ± 0.007	29	0.768 ± 0.057	71	0.574	1.373
8	0.070 ± 0.006	26	0.760 ± 0.005	74	0.581	1.342
9	0.090 ± 0.007	28	0.774 ± 0.005	72	0.582	1.364

Table S6.2. Fluorescence lifetimes (τ) of **7b** in water solution with different pH values. τ is the fluorescence lifetime of the corresponding exponential component; A is a relative contribution of the exponential decay component; τ_m is an amplitude weighted lifetime; χ2 is the Pearson criterion. SD is standard deviation.

pH value	$\tau_1 \pm$ SD, ns	A_1, %	$\tau_2 \pm$ SD, ns	A_2, %	τ_m, ns	χ^2
3	0.894 ± 0.003	50	3.244 ± 0.019	50	2.069	1.293
4	0.105 ± 0.002	75	3.358 ± 0.038	26	0.918	1.264
5	0.113 ± 0.020	83	3.405 ± 0.006	17	0.673	1.339
6	0.121 ± 0.003	92	3.000 ± 0.089	8	0.351	1.224
7	0.116 ± 0.003	92	2.363 ± 0.078	8	0.296	1.348
8	0.116 ± 0.002	87	2.842 ± 0.059	13	0.470	1.234
9	0.108 ± 0.003	85	2.856 ± 0.054	15	0.520	1.210

Table S6.3. Fluorescence lifetimes (τ) of **7c** in water solution with different pH values. τ is the fluorescence lifetime of the corresponding exponential component; A is a relative contribution of the exponential decay component; τ_m is an amplitude weighted lifetime; χ2 is the Pearson criterion. SD is standard deviation.

pH value	$\tau_1 \pm$ SD, ns	A_1, %	$\tau_2 \pm$ SD, ns	A_2, %	τ_m, ns	χ^2
3	0.134 ± 0.007	42	0.737 ± 0.008	58	0.484	1.325
4	0.124 ± 0.006	40	0.739 ± 0.007	60	0.493	1.355
5	0.104 ± 0.005	36	0.793 ± 0.007	64	0.545	1.302
6	0.098 ± 0.006	26	0.899 ± 0.005	74	0.691	1.424
7	0.079 ± 0.008	21	0.931 ± 0.004	79	0.752	1.342
8	0.087 ± 0.006	21	0.933 ± 0.005	79	0.755	1.378
9	0.063 ± 0.005	22	0.936 ± 0.004	78	0.744	1.415

7. Synthesis results

1,4-Diethyl-1,2,3,4-tetrahydroquinoxaline[2]

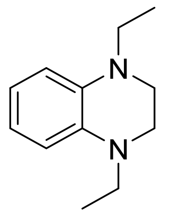

Yield 11.31 g (74%), brown oil.

^{1}H NMR (300 MHz, $CDCl_3$) δ ppm 6.54 - 6.70 (m, 4H), 3.27 - 3.43 (m, 8H), 1.17 (t, *J*=7.0 Hz, 6H). ^{1}H spectrum corresponds to literature data[2].

1,4-Diethyl-1,2,3,4-tetrahydroquinoxaline-6-carbaldehyde (1)[3]

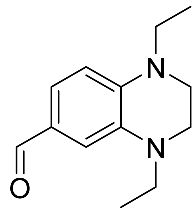

Yield 6.36 g (53%), bright yellow oil.

^{1}H NMR (300 MHz, $CDCl_3$) δ ppm 9.65 (s, 1H), 7.16 (dd, *J*=8.3, 1.5 Hz, 1H), 7.07 (s, 1H), 6.56 (d, *J*=8.2 Hz, 1H), 3.48 - 3.53 (m, 2H), 3.41 (m, *J*=11.2, 7.1 Hz, 4H), 3.26 - 3.31 (m, 2H), 1.16 - 1.24 (m, 6H). ^{1}H spectrum corresponds to literature data[3].

(Z)-5-((1,4-diethyl-1,2,3,4-tetrahydroquinoxalin-6-yl)methylene)-2,3-dimethyl-3,5-dihydro-4*H*-imidazol-4-one (2)

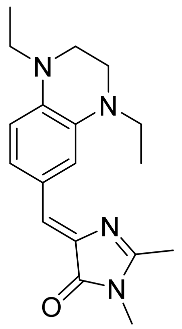

Yield 5.37 g (72 %), orange solid m.p. 175-176 °C.

^{1}H NMR (800 MHz, DMSO-d_6) δ ppm 7.62 (s, 1H), 7.35 (d, *J*=8.2 Hz, 1H), 6.79 (s, 1H), 6.52 (d, *J*=8.4 Hz, 1H), 3.41 - 3.43 (m, 2H), 3.37 (q, *J*=7.0 Hz, 2H), 3.30 - 3.33 (m, 2H), 3.22 - 3.24 (m, 2H), 3.07 (s, 3H), 2.29 (s, 3H), 1.12 (t, *J*=7.0 Hz, 3H), 1.09 (t, *J*=7.1 Hz, 3H).

^{13}C NMR (201 MHz, DMSO-d_6) δ ppm 169.6, 159.3, 137.7, 134.1, 134.0, 127.3, 124.9, 122.4, 113.5, 109.2, 46.4, 44.9, 44.7, 44.5, 26.0, 15.3, 10.3, 9.7.

HRMS found, m/z: 313.2028 $[M+H]^+$. Calculated for $C_{26}H_{30}BF_2N_4O_2^+$, m/z: 313.2023.

(Z)-5-((1,4-Diethyl-1,2,3,4-tetrahydroquinoxalin-6-yl)methylene)-3-methyl-2-((E)-styryl)-3,5-dihydro-4*H*-imidazol-4-one (5a)

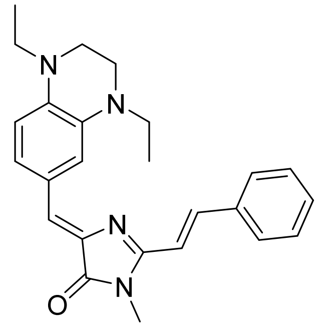

Yield 42 mg (21 %), dark blue solid m.p. 208-210 °C.

^{1}H NMR (700 MHz, DMSO-*d*$_6$) δ ppm 7.98 (br. s., 1H), 7.85 (d, *J*=15.8 Hz, 1H), 7.75 (d, *J*=7.6 Hz, 2H), 7.47 (t, *J*=7.5 Hz, 2H), 7.39 - 7.44 (m, 1H), 7.29 (br. s., 1H), 7.21 (d, *J*=15.8 Hz, 1H), 6.87 (s, 1H), 6.56 (d, *J*=8.4 Hz, 1H), 3.47 (t, *J*=4.9 Hz, 2H), 3.39 - 3.45 (m, 4H), 3.24 - 3.29 (m, 5H), 1.24 (t, *J*=7.1 Hz, 4H), 1.11 (t, *J*=7.1 Hz, 3H).

^{13}C NMR (176 MHz, CDCl3) δ ppm 170.7, 155.4, 138.5, 138.4, 135.7, 135.2, 134.4, 129.9, 129.5, 128.9, 127.5, 126.5, 123.8, 114.1, 113.4, 109.5, 47.3, 45.6 (s, 2 C), 45.4, 26.5, 10.7, 10.4.

HRMS found, m/z: 401.2323 [M+H]$^+$. Calculated for $C_{25}H_{29}N_4O^+$, m/z: 401.2336.

(Z)-5-((1,4-diethyl-1,2,3,4-tetrahydroquinoxalin-6-yl)methylene)-3-methyl-2-((E)-2-(pyridin-4-yl)vinyl)-3,5-dihydro-4*H*-imidazol-4-one (5b)

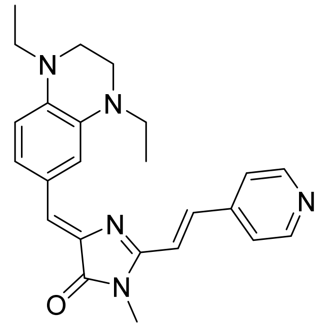

Yield 120 mg (60 %), dark blue solid m.p. 207-208 °C.

^{1}H NMR (700 MHz, DMSO-*d*$_6$) δ ppm 8.65 (d, *J*=6.0 Hz, 2H), 7.92 (br. s., 1H), 7.76 (d, *J*=15.8 Hz, 1H), 7.70 (d, *J*=6.0 Hz, 2H), 7.47 (d, *J*=15.8 Hz, 1H), 7.36 (br. s., 1H), 6.94 (s, 1H), 6.57 (d, *J*=8.4 Hz, 1H), 3.46 - 3.50 (m, 2H), 3.39 - 3.44 (m, 4H), 3.25 - 3.29 (m, 5H), 1.22 (t, *J*=7.1 Hz, 3H), 1.12 (t, *J*=7.1 Hz, 3H).

^{13}C NMR (201 MHz, DMSO-*d*$_6$) δ ppm 169.4, 154.9, 150.3, 142.3, 138.7, 134.4, 134.2, 134.1, 129.1, 126.6, 122.9, 121.6, 119.1, 113.2, 109.2, 46.6, 44.9, 44.8, 44.6, 26.2, 10.5, 9.8.

HRMS found, m/z: 402.2288 [M+H]$^+$. Calculated for $C_{24}H_{28}N_5O^+$, m/z: 402.2288.

(Z)-5-((7-(difluoroboraneyl)-1,4-diethyl-1,2,3,4-tetrahydroquinoxalin-6-yl)methylene)-2,3-dimethyl-3,5-dihydro-4*H*-imidazol-4-one (3)

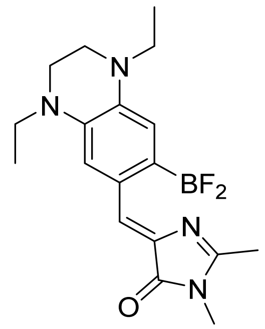

Yield 510 mg (15 %), dark blue solid m.p. 239-241 °C with decomp.

^{1}H NMR (700 MHz, DMSO-d_6) δ ppm 7.40 (s, 1H), 6.73 (s, 1H), 6.69 (s, 1H), 3.48 - 3.50 (m, 2H), 3.44 (q, *J*=7.1 Hz, 2H), 3.27 - 3.29 (m, 2H), 3.17 - 3.21 (m, 5H), 2.67 (s, 3H), 1.13 (t, *J*=7.1 Hz, 3H), 1.09 (t, *J*=7.0 Hz, 3H).

^{13}C NMR (201 MHz, DMSO-d_6) δ ppm 161.7, 160.4, 140.1, 133.3, 130.6, 122.4, 121.1, 113.5, 112.3, 46.9, 44.7, 44.3 (s, 2 C), 26.1, 12.4, 10.7, 9.6.

HRMS found, m/z: 361.2014 [M+H]$^+$. Calculated for $C_{26}H_{30}BF_2N_4O_2^+$, m/z: 361.2006.

(Z)-5-((5-(difluoroboraneyl)-1,4-diethyl-1,2,3,4-tetrahydroquinoxalin-6-yl)methylene)-2,3-dimethyl-3,5-dihydro-4*H*-imidazol-4-one (4)

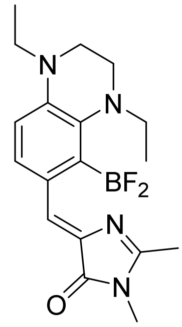

Yield 623 mg (18 %), dark red solid m.p. 216-219 °C.

^{1}H NMR (800 MHz, DMSO-d_6) δ ppm 7.38 (s, 1H), 7.22 (d, *J*=8.5 Hz, 1H), 6.71 (d, *J*=8.6 Hz, 1H), 3.46 (q, *J*=7.0 Hz, 2H), 3.35 (t, *J*=5.0 Hz, 2H), 3.20 (s, 3H), 2.94 (t, *J*=5.1 Hz, 2H), 2.87 (q, *J*=6.9 Hz, 2H), 2.68 (s, 3H), 1.12 (q, *J*=6.8 Hz, 6H).

^{13}C NMR (201 MHz, DMSO-d_6) δ ppm 161.9, 159.6, 144.6, 140.4, 132.1, 130.9, 122.8, 120.6, 109.6, 49.3, 44.8, 41.7, 40.3, 26.2, 13.4, 12.4, 10.8.

HRMS found, m/z: 361.2016 [M+H]$^+$. Calculated for $C_{26}H_{30}BF_2N_4O_2^+$, m/z: 361.2006.

(Z)-5-((7-(difluoroboraneyl)-1,4-diethyl-1,2,3,4-tetrahydroquinoxalin-6-yl)methylene)-3-methyl-2-((E)-styryl)-3,5-dihydro-4*H*-imidazol-4-one (6a)

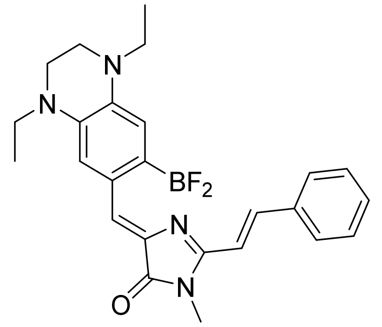

Yield 19 mg (21%), dark green solid m.p. 287-290 °C with decomp.

^{1}H NMR (800 MHz, DMSO-d_6) δ ppm 8.06 (d, *J*=16.8 Hz, 1H), 7.75 (d, *J*=8.2 Hz, 2H), 7.51 - 7.55 (m, 3H), 7.45 (s, 1H), 7.42 (d, *J*=16.9 Hz, 1H), 6.76 (s, 1H), 6.75 (s, 1H), 3.51 - 3.55 (m, 5H), 3.48 (q, *J*=7.1 Hz, 2H), 3.31 - 3.33 (m, 2H), 3.20 (t, *J*=4.9 Hz, 2H), 1.15 (t, *J*=7.1 Hz, 3H), 1.10 (t, *J*=7.1 Hz, 3H).

^{13}C NMR – poor solubility in $CDCl_3$/DMSO.

HRMS found, m/z: 449.2323 $[M+H]^+$. Calculated for $C_{25}H_{28}BF_2N_4O^+$, m/z:. 449.2319

(Z)-5-((7-(difluoroboraneyl)-1,4-diethyl-1,2,3,4-tetrahydroquinoxalin-6-yl)methylene)-2-((E)-4-methoxystyryl)-3-methyl-3,5-dihydro-4*H*-imidazol-4-one (6c)

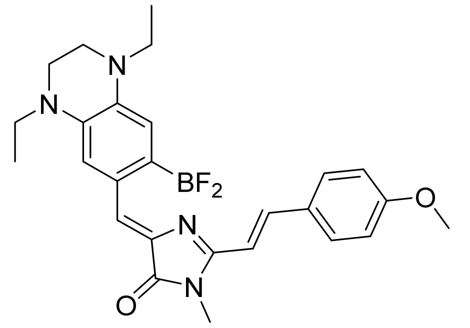

Yield 16 mg (17%), dark green solid m.p. 295-297 °C with decomp.

^{1}H NMR (800 MHz, DMSO-d_6) δ ppm 8.06 (d, *J*=16.8 Hz, 1H), 7.72 (m, *J*=8.7 Hz, 2H), 7.40 (s, 1H), 7.27 (d, *J*=16.7 Hz, 1H), 7.10 (m, *J*=8.8 Hz, 2H), 6.74 (s, 2H), 3.85 (s, 3H), 3.50 - 3.54 (m, 5H), 3.48 (q, *J*=7.0 Hz, 2H), 3.30 - 3.32 (m, 2H), 3.20 (t, *J*=4.9 Hz, 2H), 1.15 (t, *J*=7.1 Hz, 3H), 1.10 (t, *J*=7.0 Hz, 3H).

^{13}C NMR – poor solubility in $CDCl_3$/DMSO.

HRMS found, m/z: 479.2425 $[M+H]^+$. Calculated for $C_{26}H_{30}BF_2N_4O_2^+$, m/z: 479.2424.

(Z)-5-((5-(difluoroboraneyl)-1,4-diethyl-1,2,3,4-tetrahydroquinoxalin-6-yl)methylene)-3-methyl-2-((E)-styryl)-3,5-dihydro-4*H*-imidazol-4-one (7a)

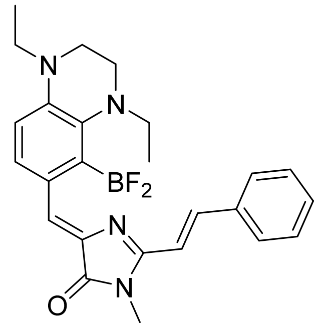

Yield 74 mg (83 %), dark blue solid m.p. 240-242 °C.

^{1}H NMR (800 MHz, DMSO-*d*$_6$) δ ppm 8.01 (d, *J*=16.9 Hz, 1H), 7.74 (d, *J*=7.3 Hz, 2H), 7.54 (t, *J*=7.3 Hz, 2H), 7.50 - 7.52 (m, 1H), 7.48 (d, *J*=16.9 Hz, 1H), 7.44 (s, 1H), 7.28 (d, *J*=8.6 Hz, 1H), 6.76 (d, *J*=8.7 Hz, 1H), 3.53 (s, 3H), 3.49 (q, *J*=7.1 Hz, 2H), 3.39 (t, *J*=5.2 Hz, 2H), 2.98 (t, *J*=5.2 Hz, 2H), 2.90 (q, *J*=6.9 Hz, 2H), 1.15 (m, 6H).

^{13}C NMR (201 MHz, DMSO-*d*$_6$) δ ppm 162.0, 152.5, 145.2, 144.1, 140.6, 135.0, 132.8, 131.3, 130.8, 129.2, 128.0, 123.4, 121.3, 111.7, 109.9, 49.4, 45.0, 41.7, 40.5, 28.4, 13.5, 10.9.

HRMS found, m/z: 449.2325 [M+H]$^+$. Calculated for $C_{25}H_{28}BF_2N_4O^+$, m/z: 449.2319.

(Z)-5-((5-(difluoroboraneyl)-1,4-diethyl-1,2,3,4-tetrahydroquinoxalin-6-yl)methylene)-3-methyl-2-((E)-2-(pyridin-4-yl)vinyl)-3,5-dihydro-4*H*-imidazol-4-one (7b)

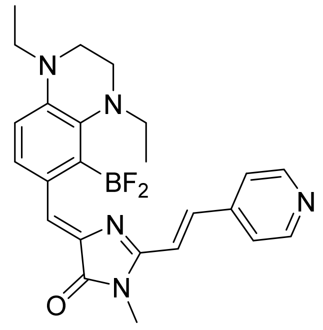

Yield 34 mg (38 %), dark blue solid m.p. 235°C with decomp.

^{1}H NMR (800 MHz, DMSO-*d*$_6$) δ ppm 8.71 - 8.74 (m, 2H), 7.94 (d, *J*=16.9 Hz, 1H), 7.62 - 7.66 (m, 3H), 7.50 (s, 1H), 7.31 (d, *J*=8.6 Hz, 1H), 6.77 (d, *J*=8.7 Hz, 1H), 3.48 - 3.53 (m, 5H), 3.40 (t, *J*=5.3 Hz, 2H), 2.98 (t, *J*=5.2 Hz, 2H), 2.90 (q, *J*=6.9 Hz, 2H), 1.15 (m, 6H).

^{13}C NMR (201 MHz, DMSO-*d*$_6$) δ ppm 161.8, 151.3, 150.6, 150.6, 145.7, 142.0, 140.8, 133.4, 132.3, 123.4, 121.5, 121.1, 115.8, 110.1, 49.4, 45.1, 41.7, 40.6, 28.4, 13.4, 11.0.

HRMS found, m/z: 450.2279 [M+H]$^+$. Calculated for $C_{24}H_{27}BF_2N_5O^+$, m/z: 450.2271.

(Z)-5-((5-(difluoroboraneyl)-1,4-diethyl-1,2,3,4-tetrahydroquinoxalin-6-yl)methylene)-2-((E)-4-methoxystyryl)-3-methyl-3,5-dihydro-4*H*-imidazol-4-one (7c)

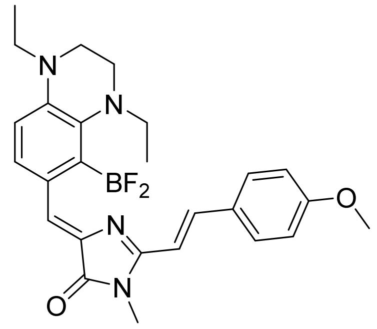

Yield 70 mg (73 %), dark blue solid m.p. 266-268 °C.

^{1}H NMR (800 MHz, DMSO-d_6) δ ppm 8.01 (d, *J*=16.8 Hz, 1H), 7.71 (m, *J*=8.6 Hz, 2H), 7.39 (s, 1H), 7.34 (d, *J*=16.8 Hz, 1H), 7.25 (d, *J*=8.5 Hz, 1H), 7.11 (m, *J*=8.6 Hz, 2H), 6.74 (d, *J*=8.6 Hz, 1H), 3.85 (s, 3H), 3.52 (s, 3H), 3.48 (q, *J*=7.0 Hz, 2H), 3.38 (t, *J*=5.0 Hz, 2H), 2.97 (t, *J*=5.0 Hz, 2H), 2.90 (q, *J*=6.8 Hz, 2H), 1.15 (m, 6H).

^{13}C NMR (201 MHz, DMSO-d_6) δ ppm 162.1, 161.7, 153.0, 145.0, 144.3, 140.6, 132.4, 130.5, 130.0, 127.7, 123.4, 121.5, 114.8, 109.9, 108.9, 55.4, 49.4, 44.9, 41.8, 40.5, 28.5, 13.5, 10.9.

HRMS found, m/z: 479.2435 [M+H]$^+$. Calculated for $C_{26}H_{30}BF_2N_4O_2^+$, m/z: 479.2424.

(Z)-2-((1,4-diethyl-1,2,3,4-tetrahydroquinoxalin-6-yl)methylene)imidazo[1,2-a]pyridin-3(2*H*)-one (8)

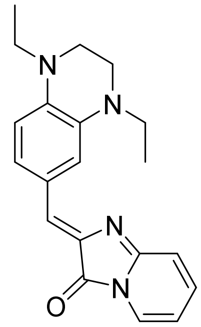

Yield 616 mg (39%), dark blue solid m.p. 77-78 °C.

^{1}H NMR (700 MHz, DMSO-d_6) δ ppm 7.70 (d, *J*=7.1 Hz, 2H), 7.56 (br. s., 1H), 7.18 (ddd, *J*=9.3, 6.4, 1.0 Hz, 1H), 7.12 (s, 1H), 6.92 (d, *J*=9.3 Hz, 1H), 6.60 (d, *J*=8.6 Hz, 1H), 6.34 (t, *J*=6.6 Hz, 1H), 3.46 - 3.50 (m, 2H), 3.42 (q, *J*=7.1 Hz, 2H), 3.34 (q, *J*=7.1 Hz, 2H), 3.21 - 3.25 (m, 2H), 1.15 (t, *J*=7.1 Hz, 3H), 1.11 (t, *J*=7.1 Hz, 3H).

^{13}C NMR (176 MHz, $CDCl_3$) δ ppm 166.5, 152.3, 139.2, 134.4, 134.2, 133.8, 132.3, 127.2, 125.4, 124.0, 119.2, 114.6, 109.7, 108.4, 47.4, 45.4, 45.3, 45.1, 10.8, 10.2.

HRMS found, m/z: 335.1863 [M+H]$^+$. Calculated for $C_{20}H_{23}N_4O^+$, m/z 335.1866.

(Z)-2-((5-(difluoroboraneyl)-1,4-diethyl-1,2,3,4-tetrahydroquinoxalin-6-yl)methylene)imidazo[1,2-a]pyridin-3(2*H*)-one (9)

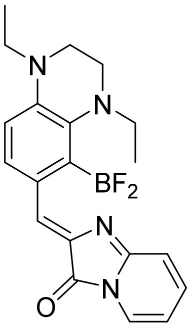

Yield 79 mg (28%), dark solid m.p. 270 °C with decomp.

^{1}H NMR (800 MHz, $CDCl_3$) δ ppm 8.09 (d, *J*=6.8 Hz, 1H), 7.84 (d, *J*=9.2 Hz, 1H), 7.60 (s, 1H), 7.58 (ddd, *J*=9.1, 6.7, 1.1 Hz, 1H), 7.22 (d, *J*=8.7 Hz, 1H), 6.78 (t, *J*=6.7 Hz, 1H), 6.71 (d, *J*=8.6 Hz, 1H), 3.52 (q, *J*=7.2 Hz, 2H), 3.45 (t, *J*=5.2 Hz, 2H), 3.26 (t, *J*=5.2 Hz, 2H), 3.09 (q, *J*=7.0 Hz, 2H), 1.32 (t, *J*=7.0 Hz, 3H), 1.26 (t, *J*=7.0 Hz, 3H).

^{13}C NMR – poor solubility in $CDCl_3$/DMSO.

HRMS found, m/z: 383.1858 $[M+H]^+$. Calculated for $C_{26}H_{30}BF_2N_4O_2^+$, m/z: 383.1849.

8. References

[1] Würth C, Grabolle M, Pauli J, Spieles M, Resch-Genger U. Relative and absolute determination of fluorescence quantum yields of transparent samples. Nat Protoc 2013;8:1535–50. https://doi.org/10.1038/nprot.2013.087.

[2] Chandrasekaran Y, Dutta GK, Kanth RB, Patil S. Tetrahydroquinoxaline based squaraines: Synthesis and photophysical properties. Dyes and Pigments 2009;83:162–7. https://doi.org/https://doi.org/10.1016/j.dyepig.2009.04.003.

[3] Tian Z, Liu Y, Tian B, Zhang J. Synthesis and proton-induced fluorescence “OFF–ON” switching of a new D-π-A type pyran dye. Research on Chemical Intermediates 2015;41:525–33. https://doi.org/10.1007/s11164-013-1206-0.

[4] Meyers AI, Lawson JP, Walker DG, Linderman RJ. Synthetic studies on the streptogramin antibiotics. Enantioselective synthesis of the oxazole dienyl amine moiety. J Org Chem 1986;51:5111–23. https://doi.org/10.1021/jo00376a011.

9. Copies of ^{1}H and ^{13}C NMR spectra

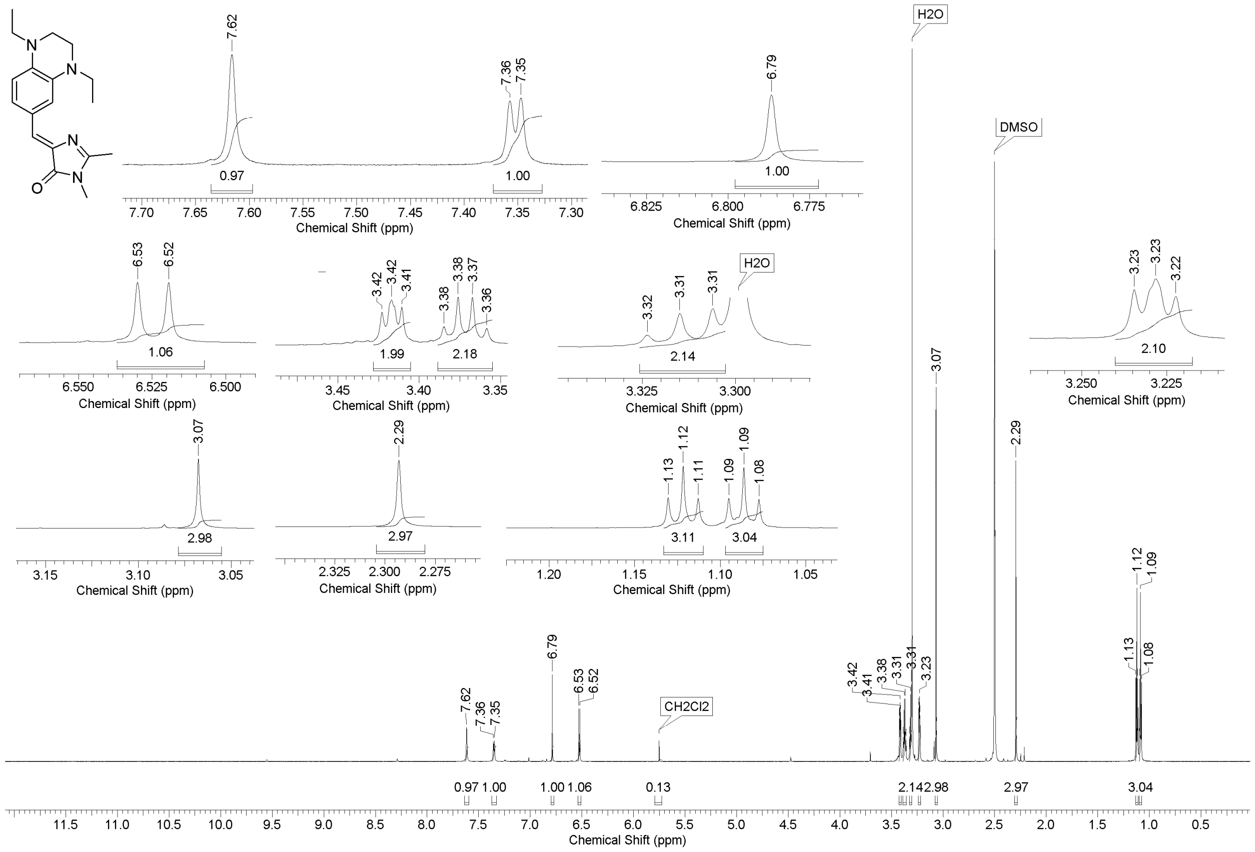

Figure S7.1. ^{1}H NMR spectrum of compound **2**

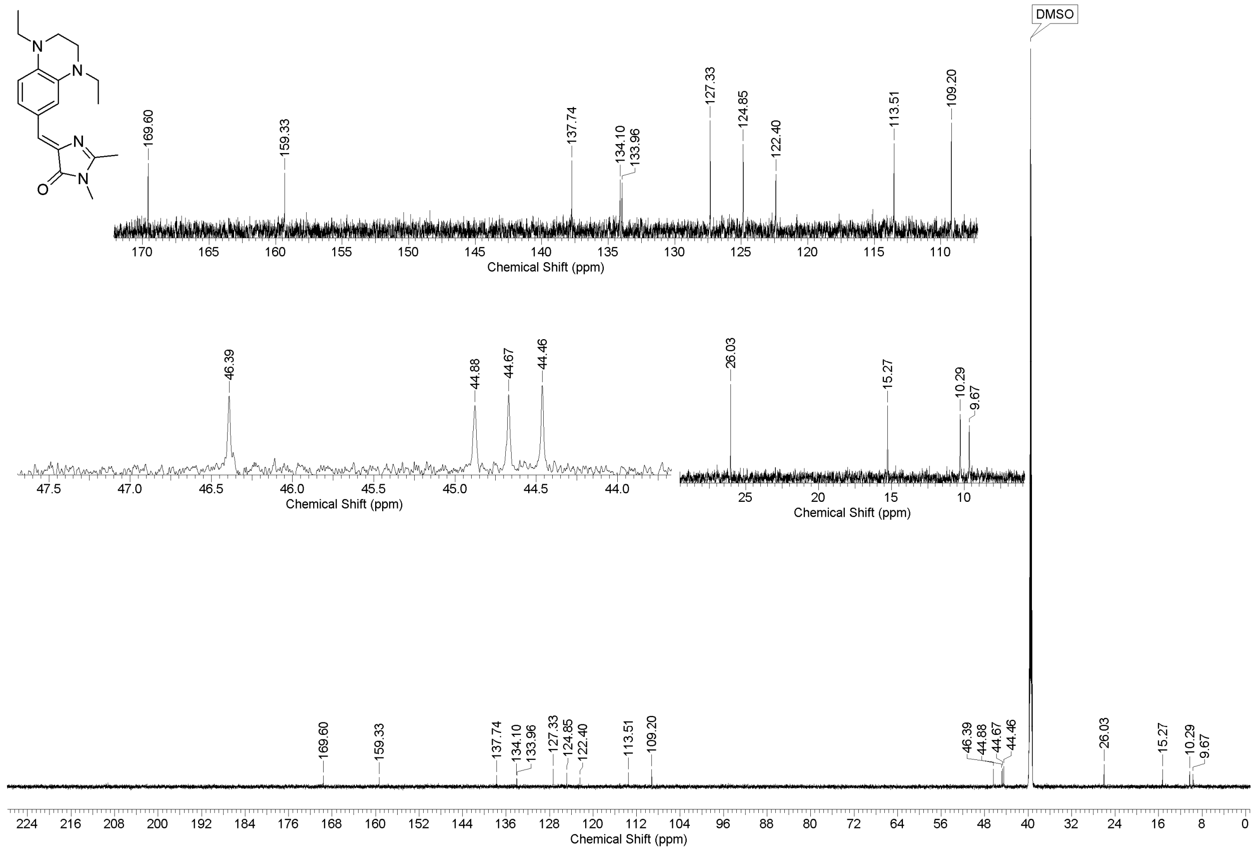

Figure S7.2. ^{13}C NMR spectrum of compound **2**

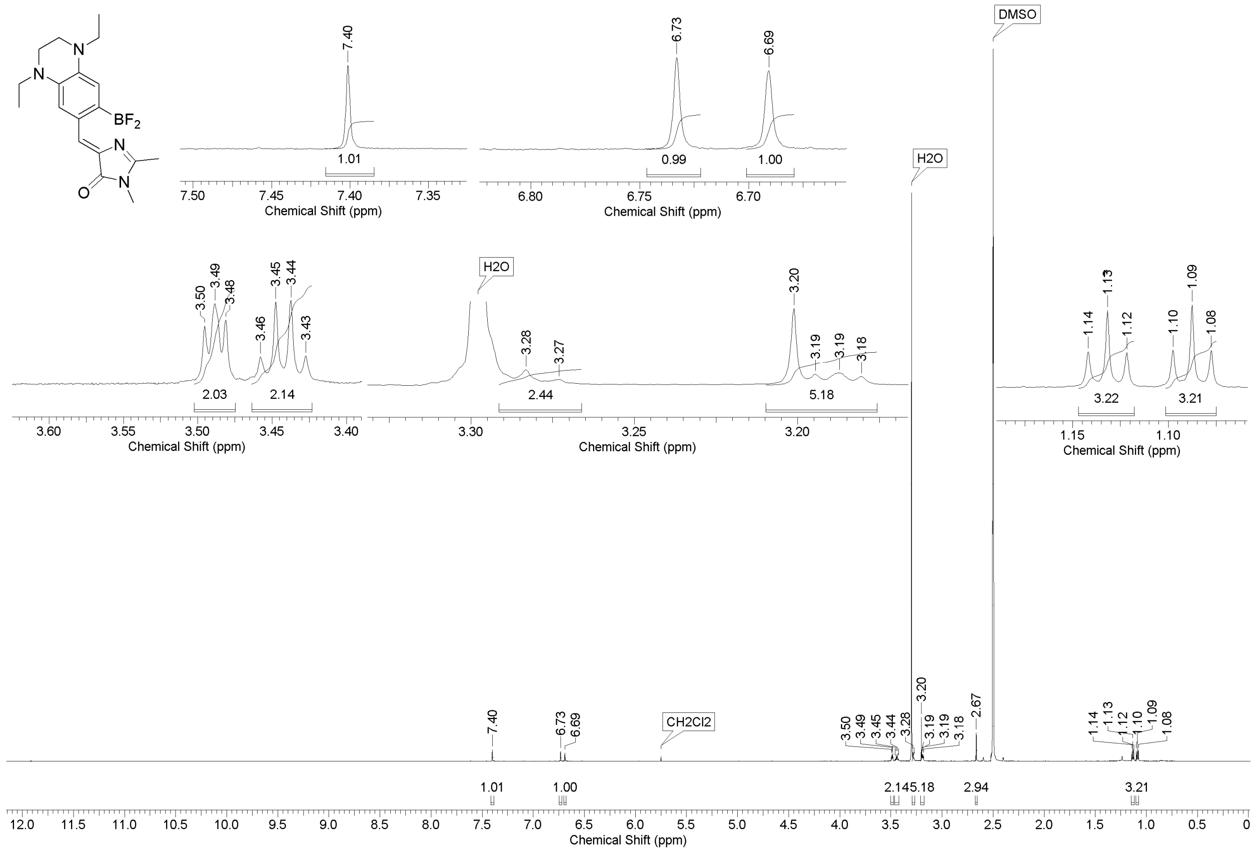

Figure S7.3. ^{1}H NMR spectrum of compound **3**

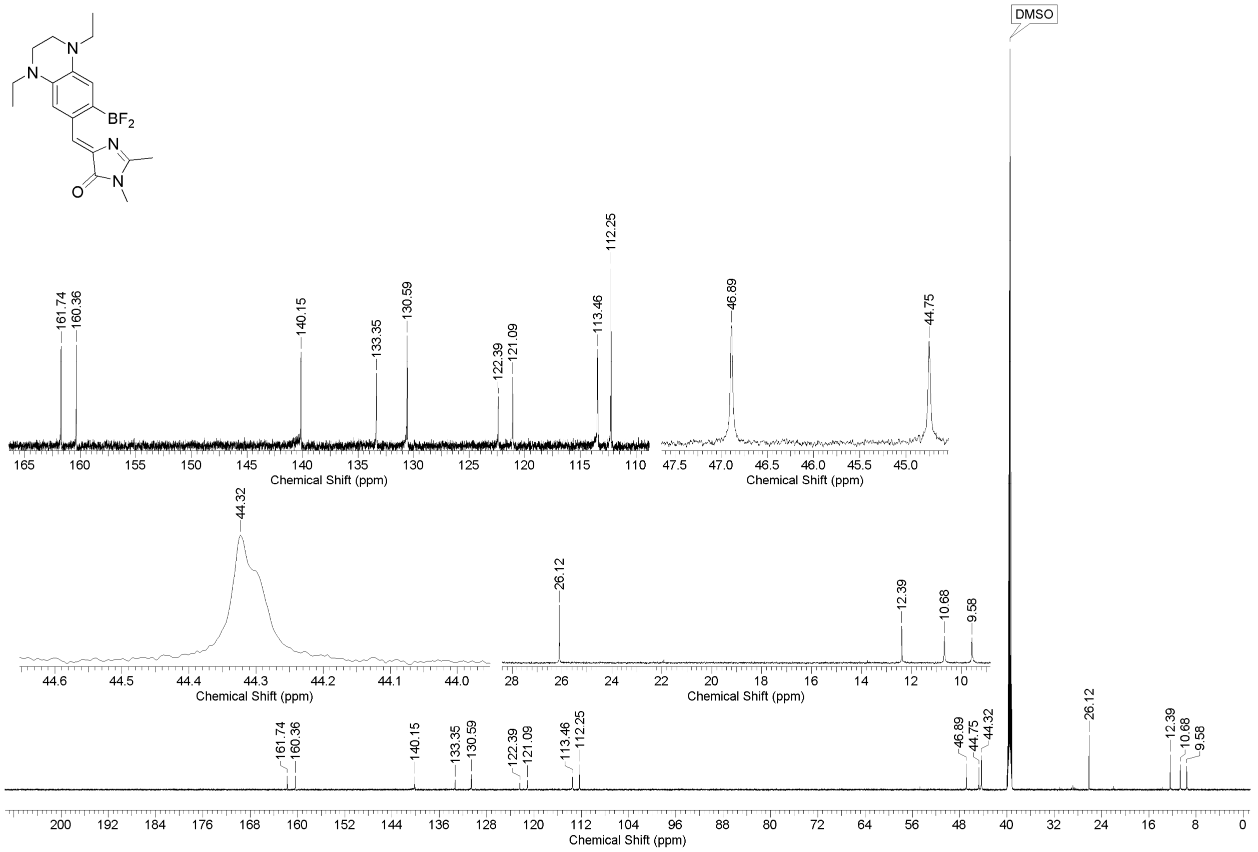

Figure S7.4. ^{13}C NMR spectrum of compound **3**

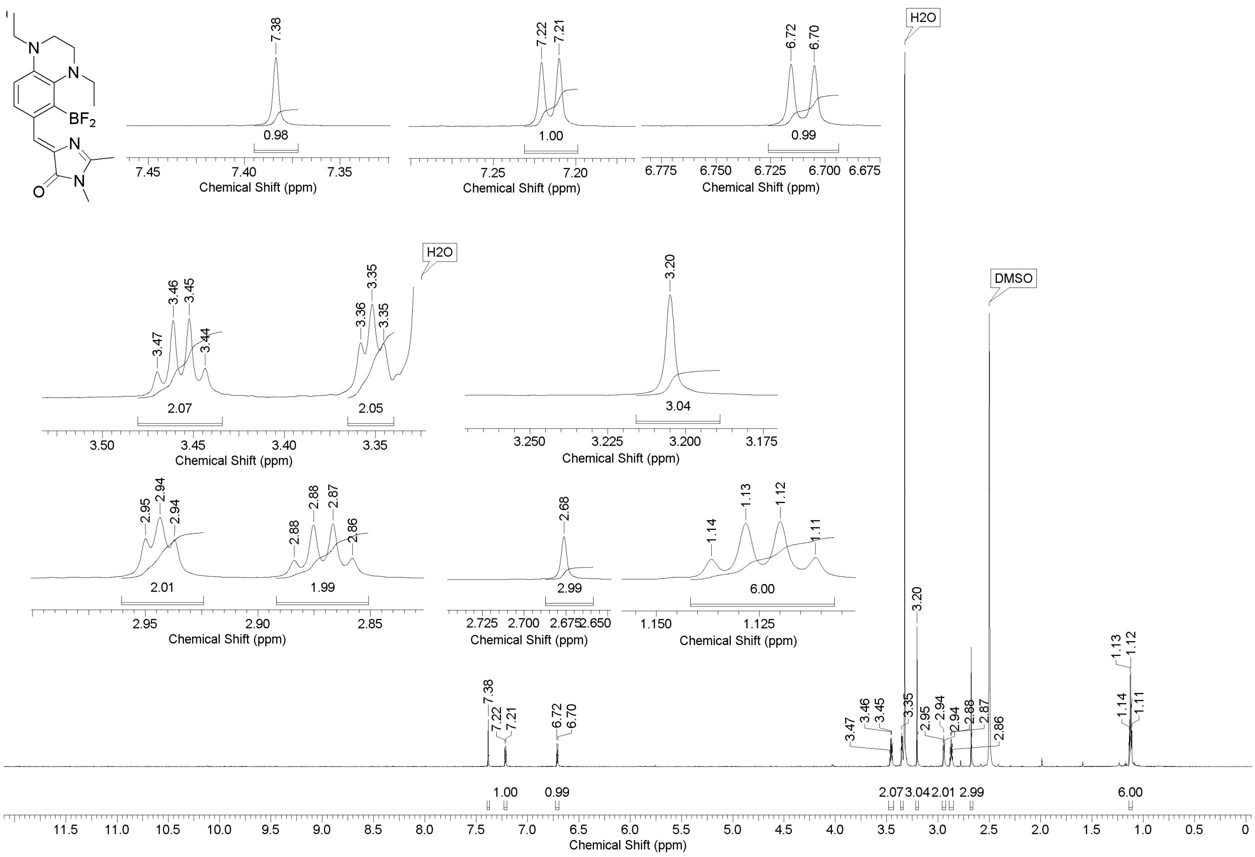

Figure S7.5. ^{1}H NMR spectrum of compound **4**

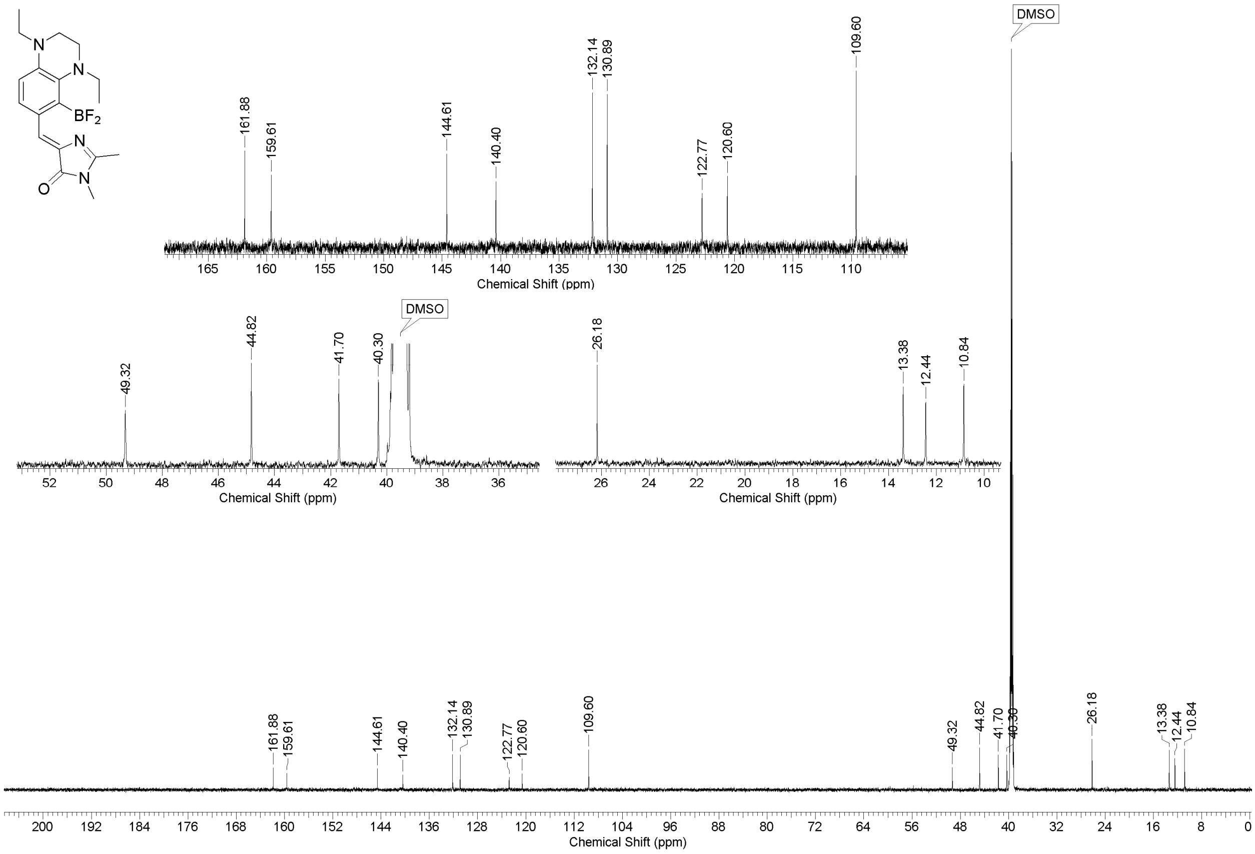

Figure S7.6. ^{13}C NMR spectrum of compound **4**

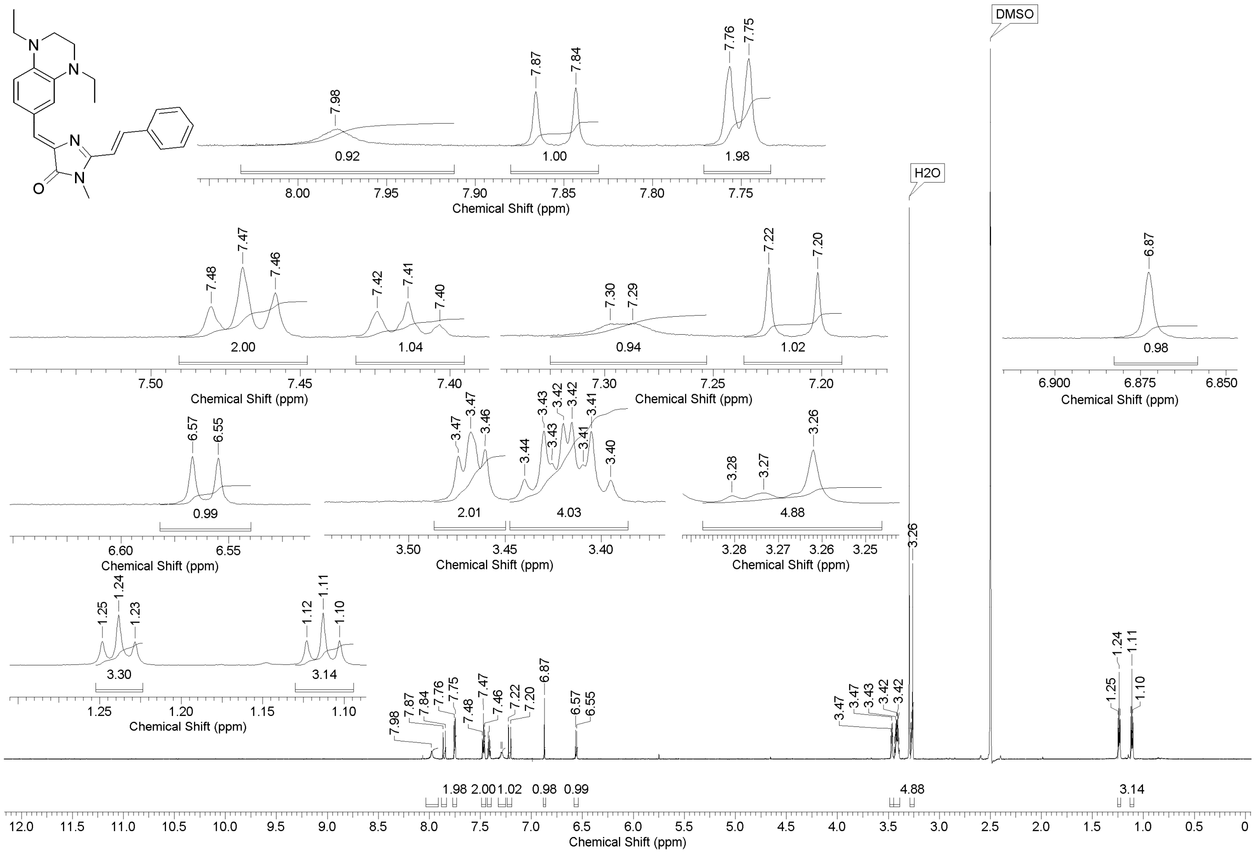

Figure S7.7. ^{1}H NMR spectrum of compound **5a**

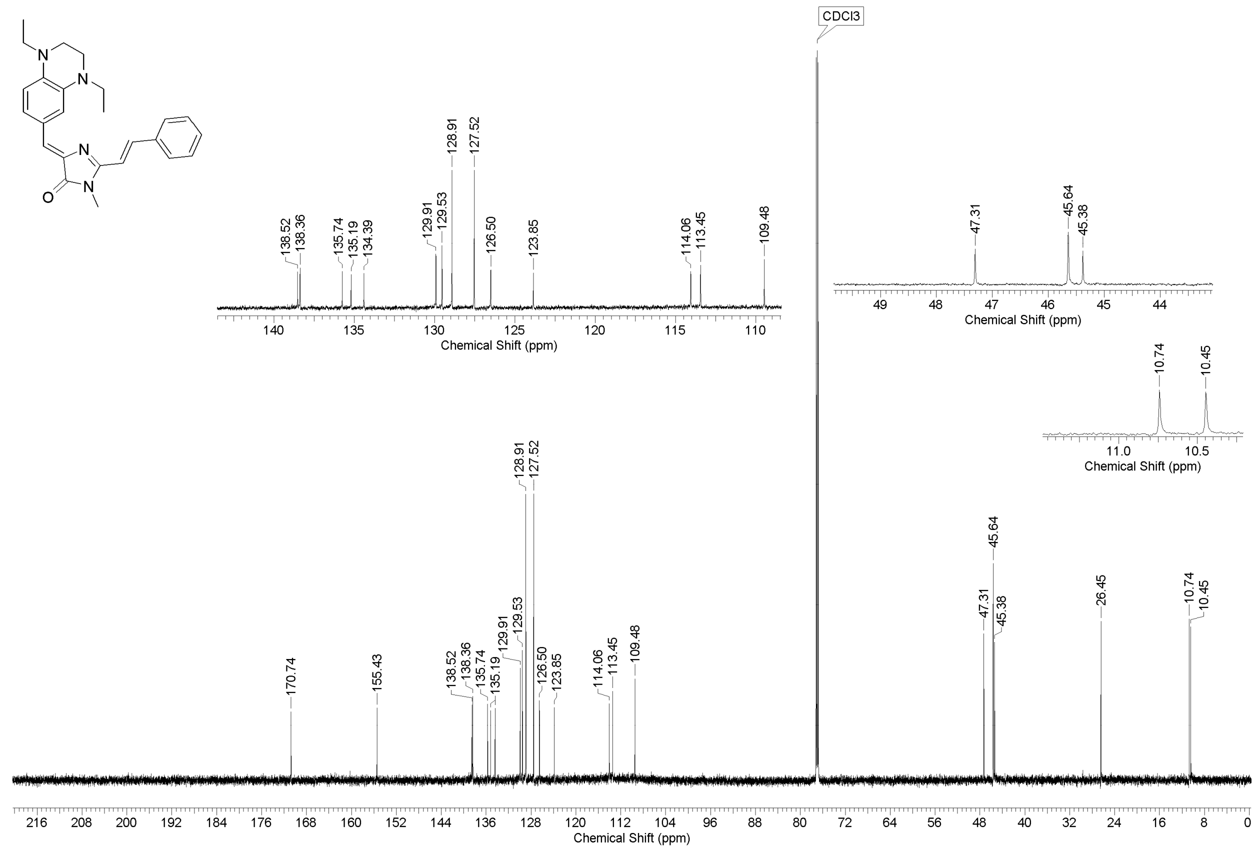

Figure S7.8. ^{13}C NMR spectrum of compound **5a**

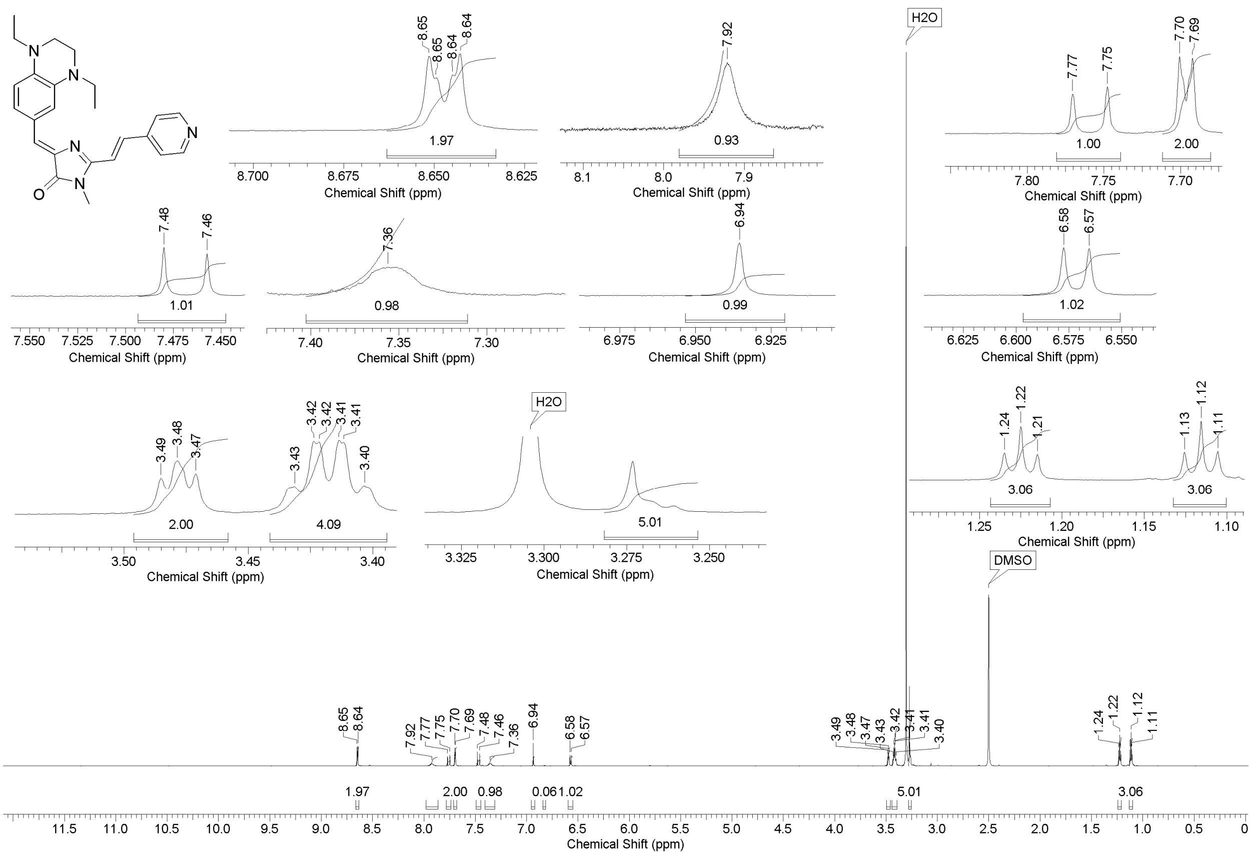

Figure S7.9. ^{1}H NMR spectrum of compound **5b**

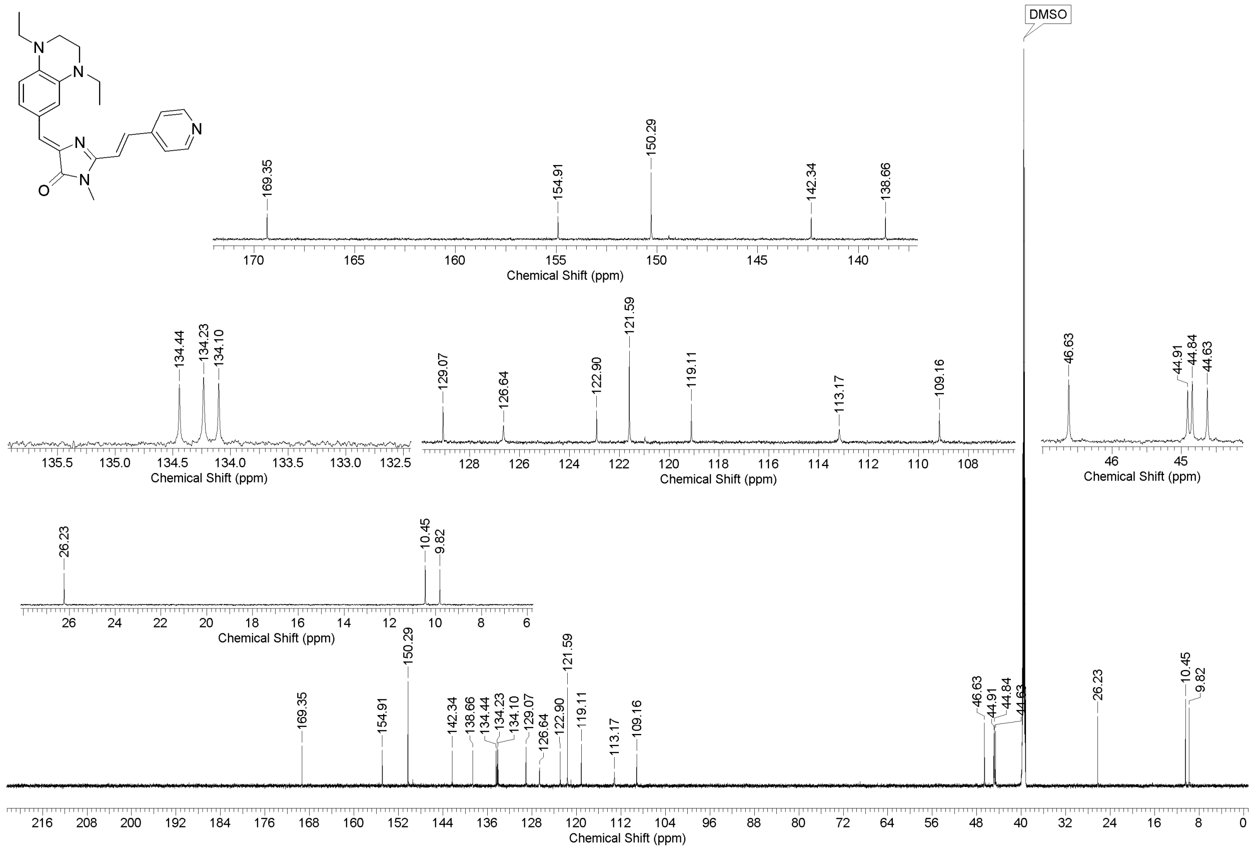

Figure S7.10. ^{13}C NMR spectrum of compound **5b**

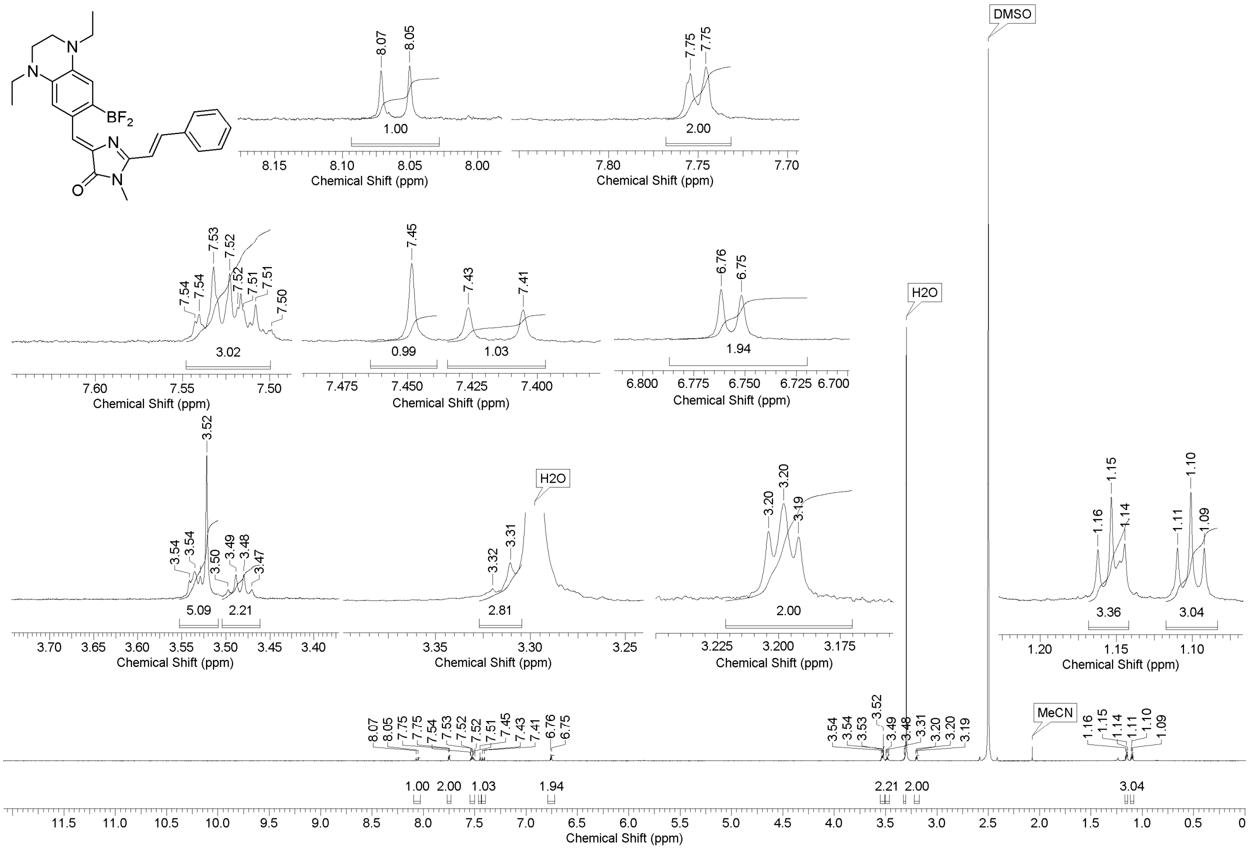

Figure S7.11. ^{1}H NMR spectrum of compound **6a**

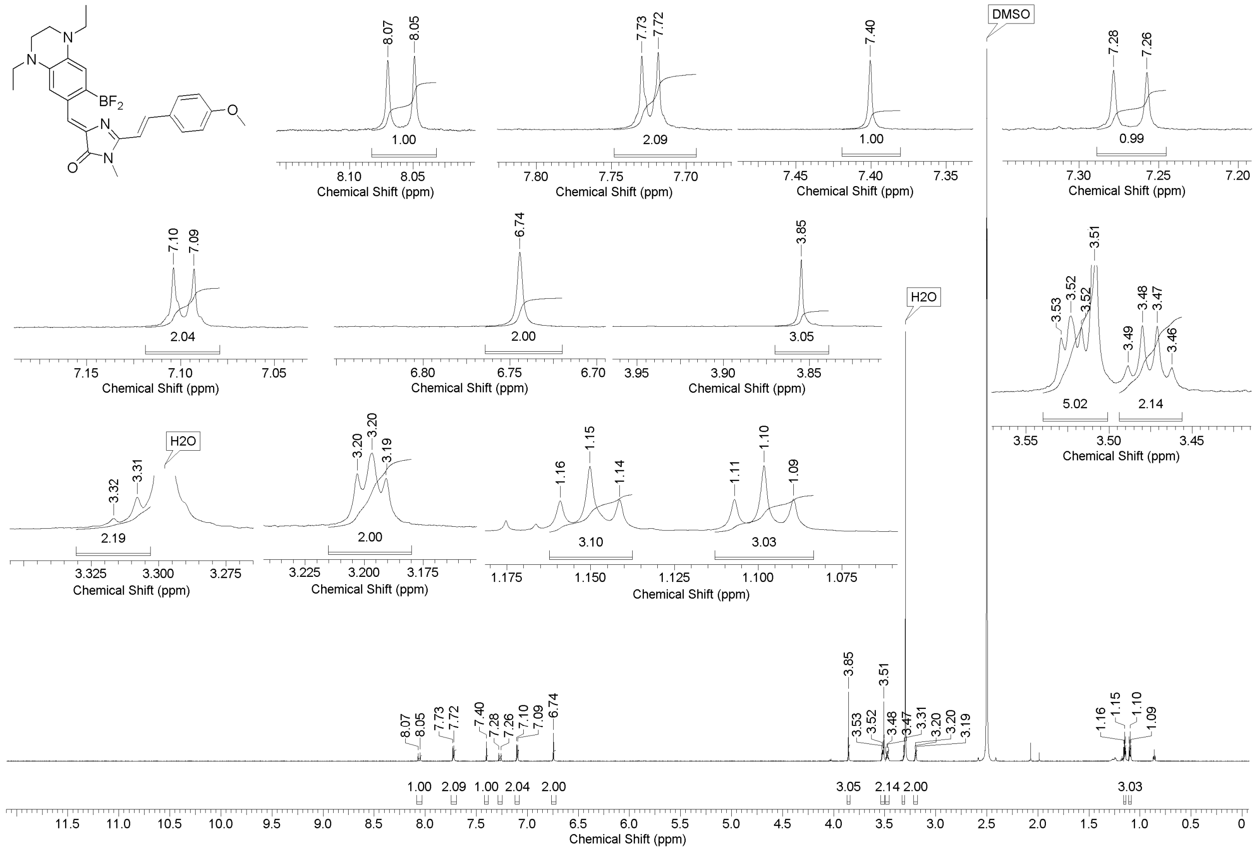

Figure S7.12. ^{1}H NMR spectrum of compound **6c**

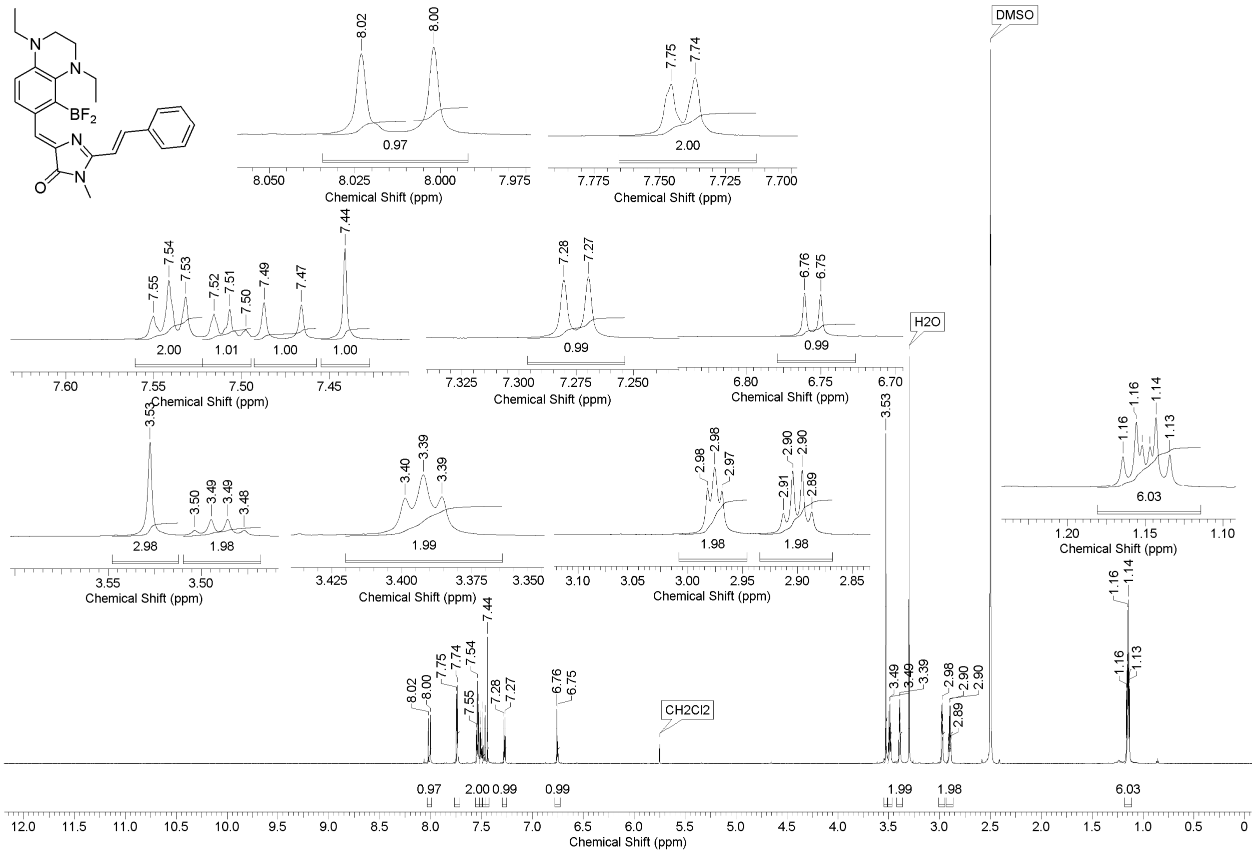

Figure S7.13. ^{1}H NMR spectrum of compound **7a**

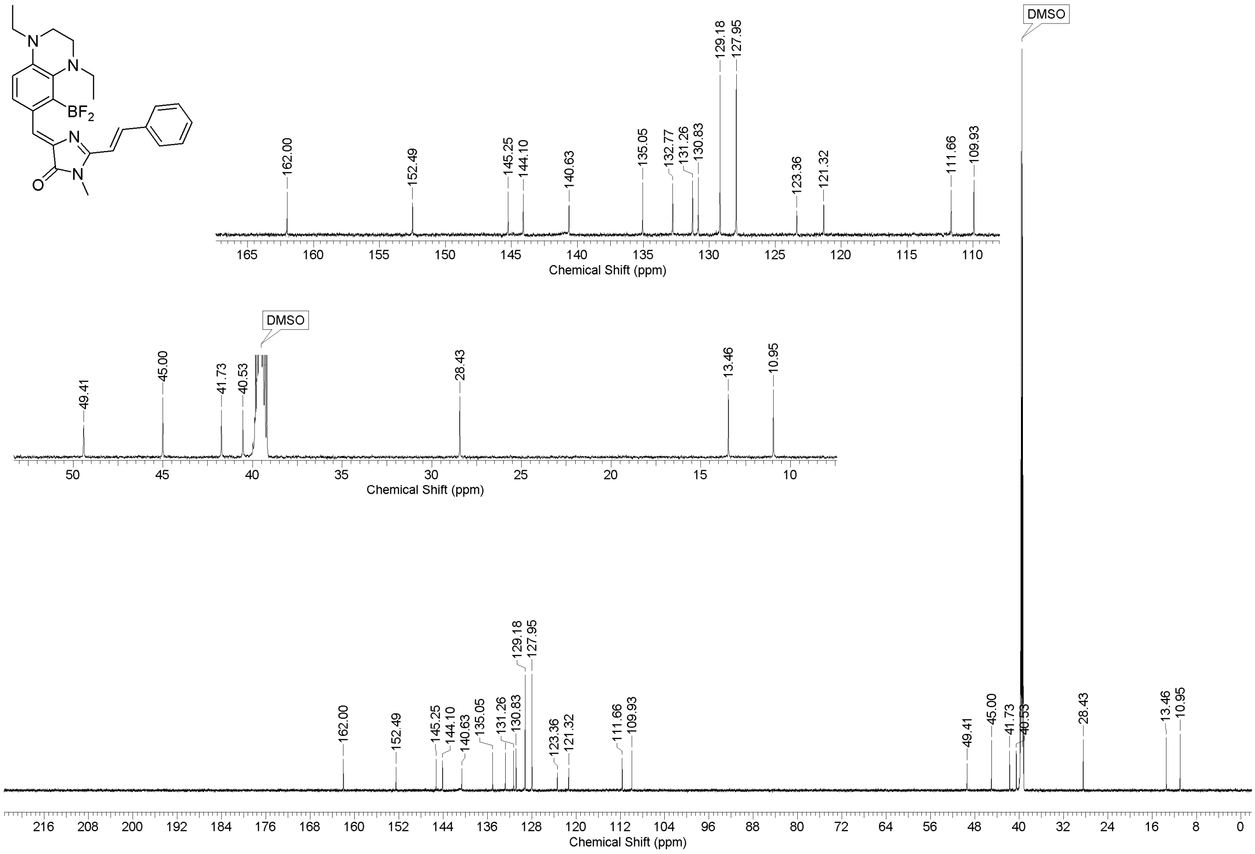

Figure S7.14. ^{13}C NMR spectrum of compound **7a**

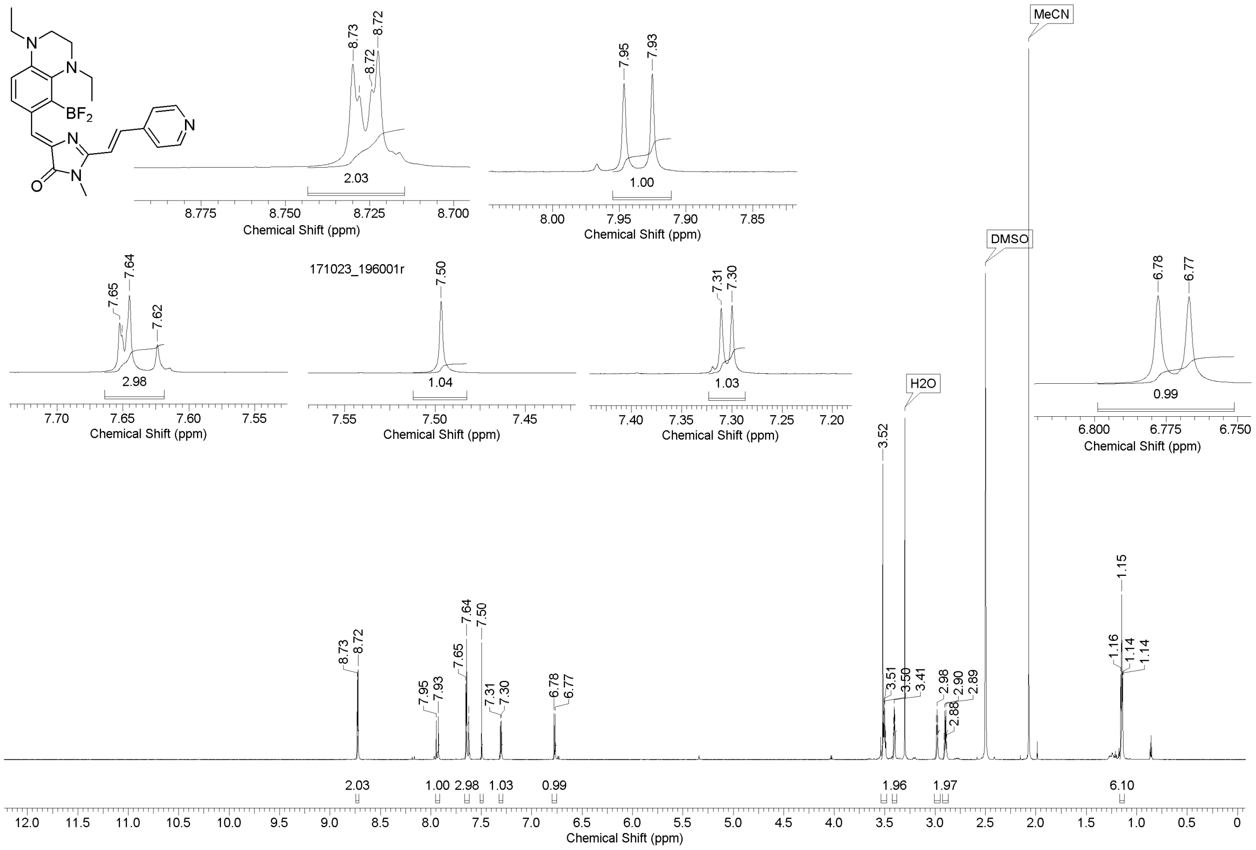

Figure S7.15. ^{1}H NMR spectrum of compound **7b**

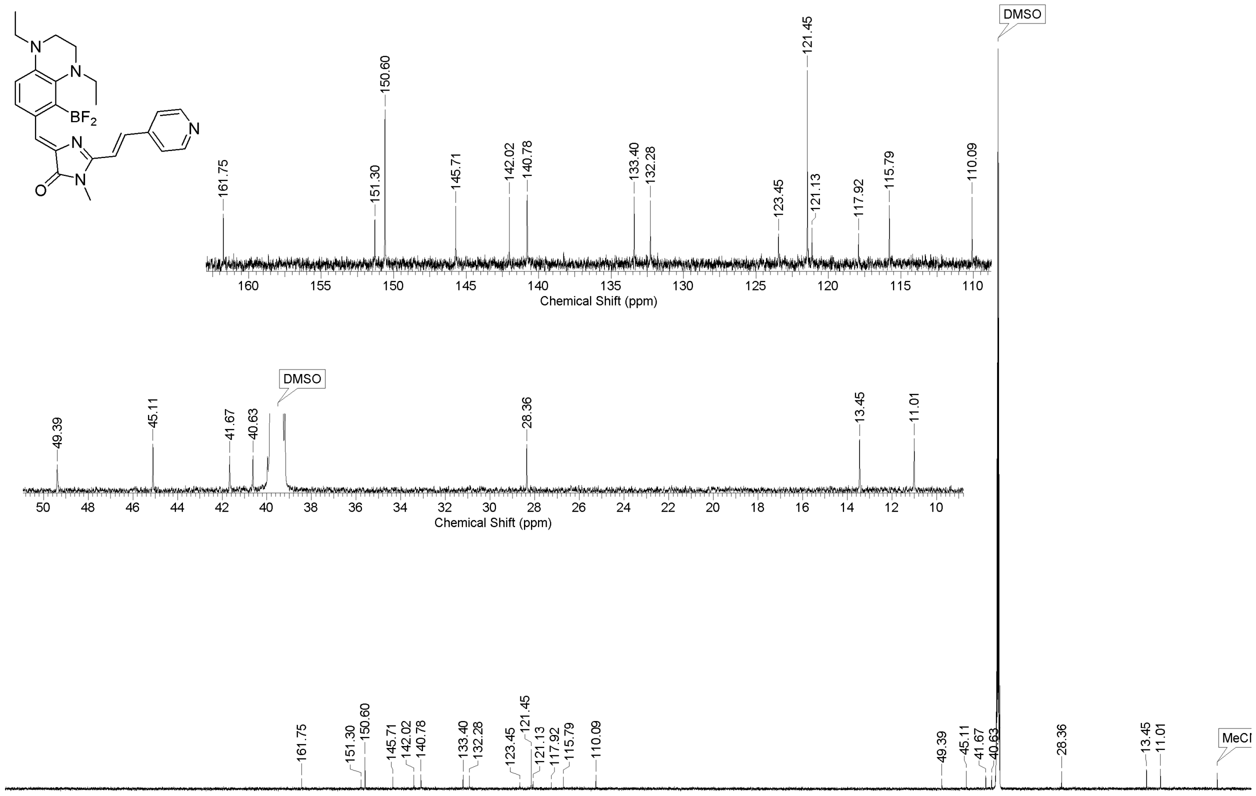

Figure S7.16. ^{13}C NMR spectrum of compound **7b**

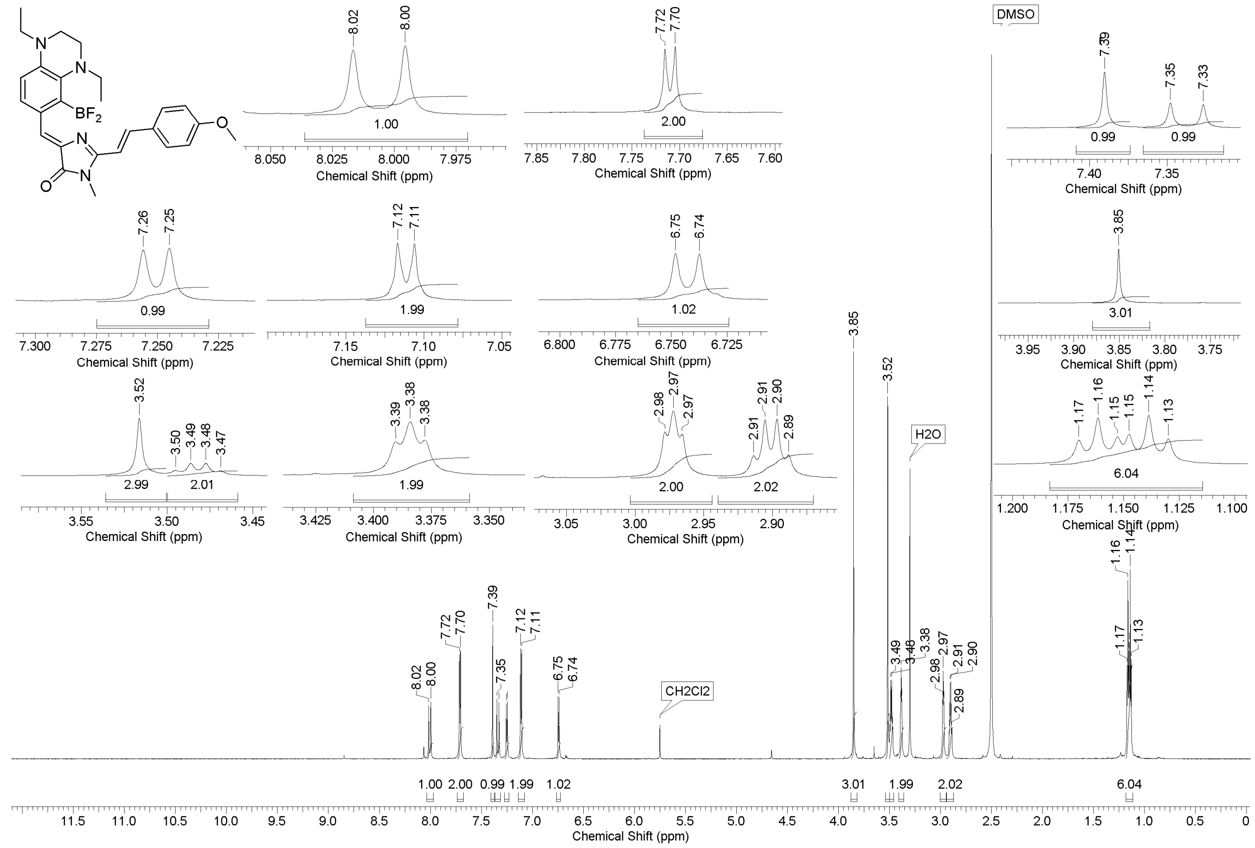

Figure S7.17. ^{1}H NMR spectrum of compound **7c**

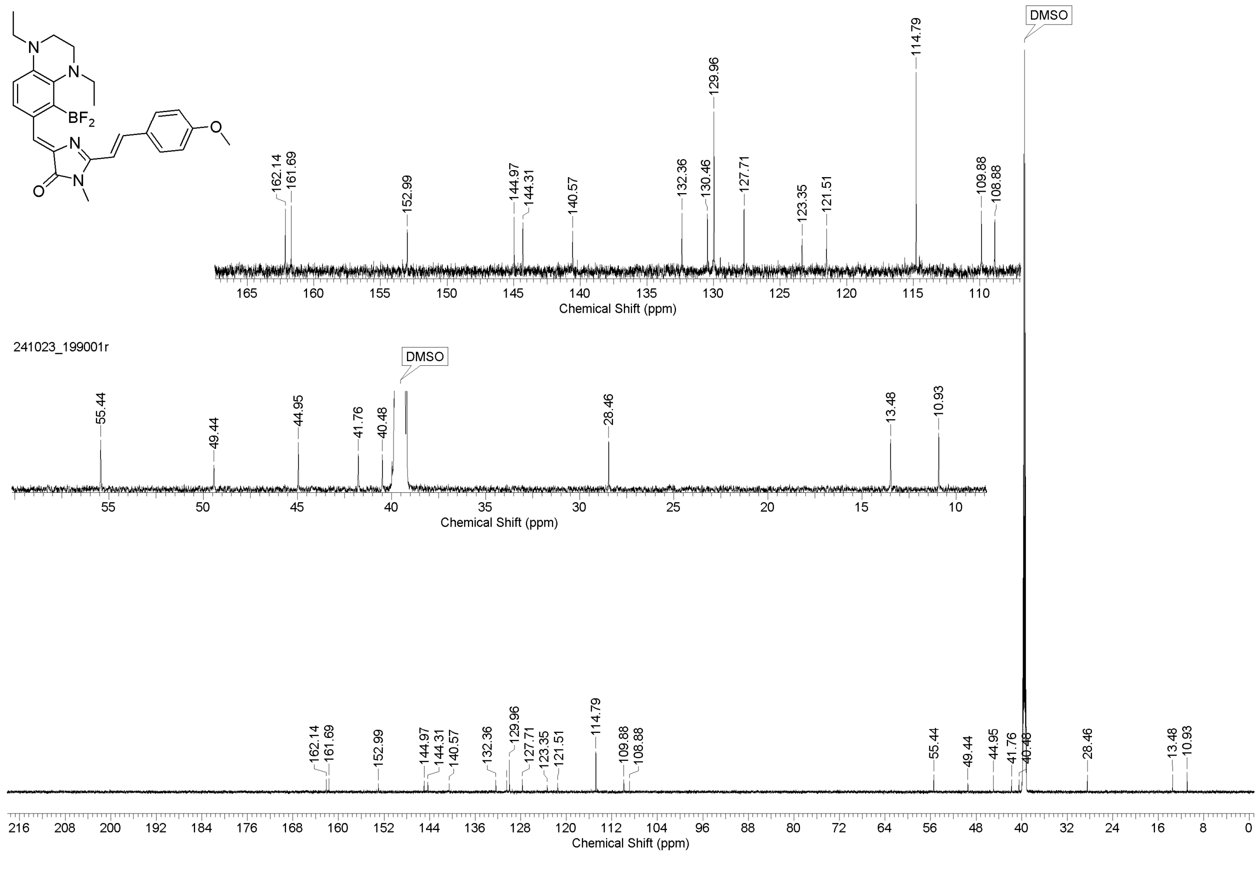

Figure S7.18. ^{13}C NMR spectrum of compound **7c**

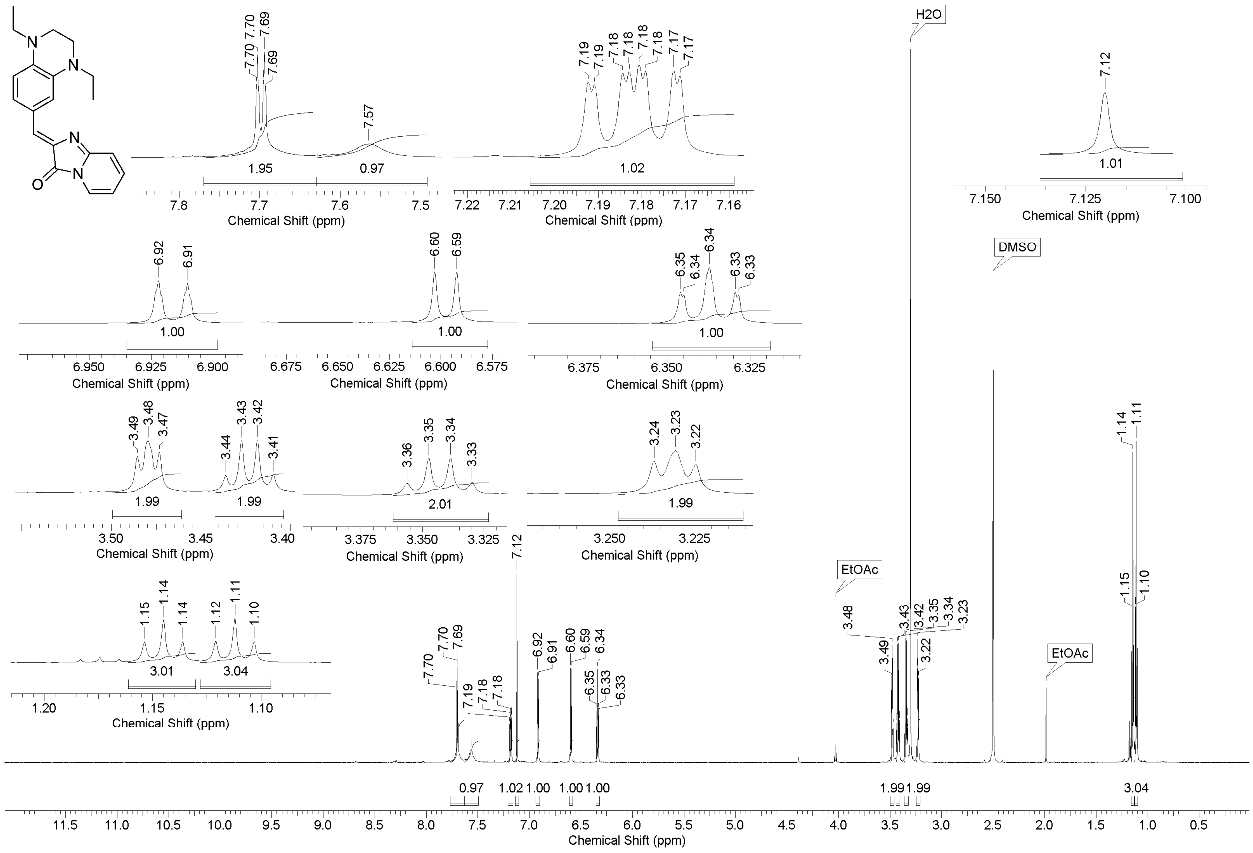

Figure S7.19. ^{1}H NMR spectrum of compound **8**

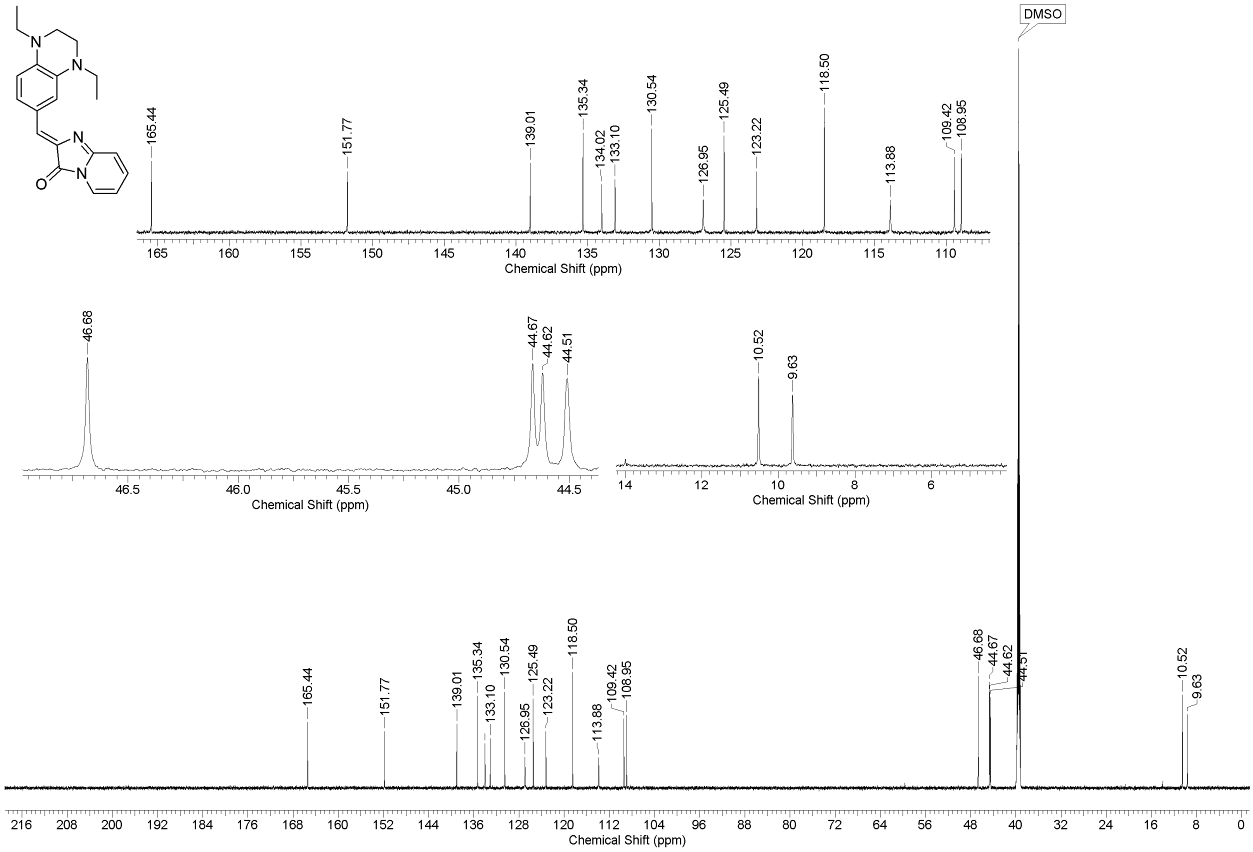

Figure S7.20. ^{13}C NMR spectrum of compound **8**

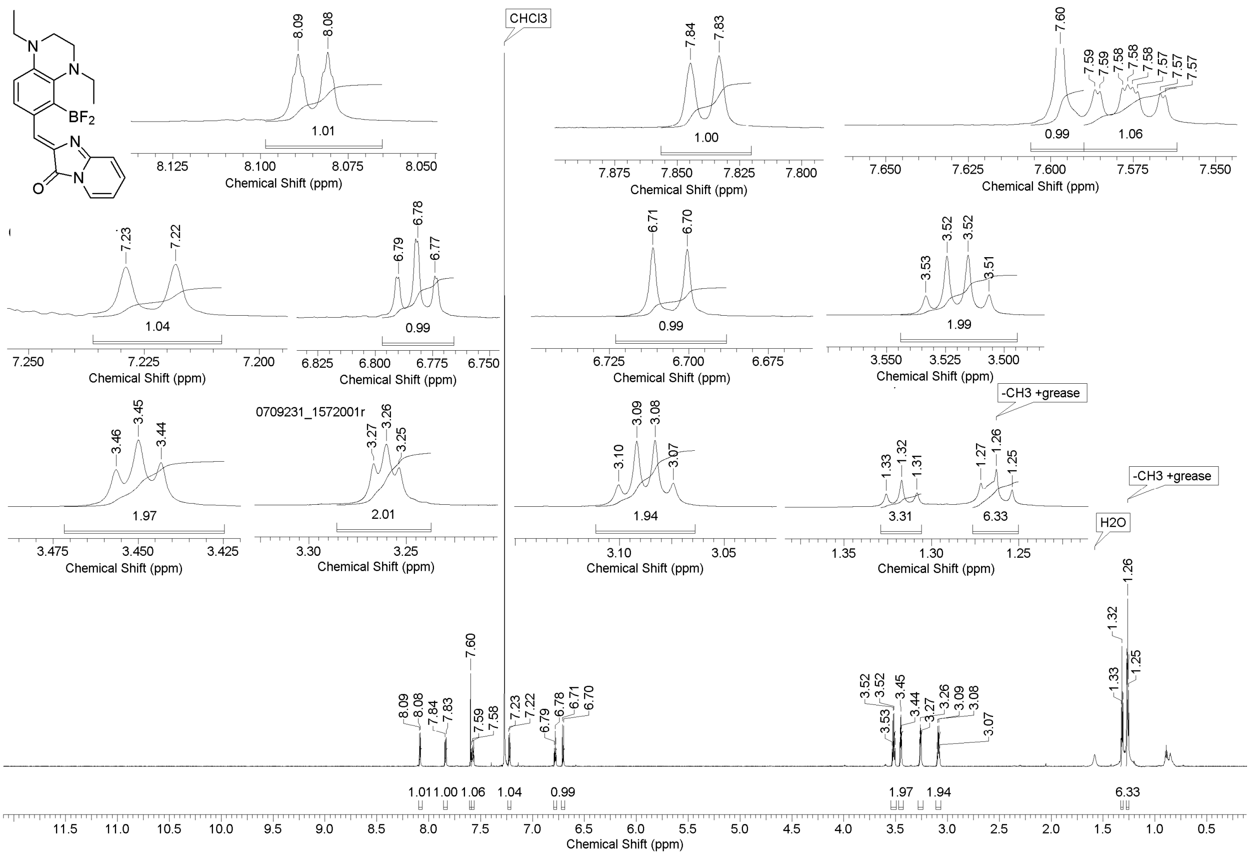

Figure S7.21. ^{1}H NMR spectrum of compound **9**
